# Phylogenomic analysis uncovers a 9-year variation of Uganda influenza type-A strains from the WHO-recommended vaccines and other Africa strains

**DOI:** 10.1038/s41598-023-30667-z

**Published:** 2023-04-04

**Authors:** Grace Nabakooza, D. Collins Owuor, Zaydah R. de Laurent, Ronald Galiwango, Nicholas Owor, John T. Kayiwa, Daudi Jjingo, Charles N. Agoti, D. James Nokes, David P. Kateete, John M. Kitayimbwa, Simon D. W. Frost, Julius J. Lutwama

**Affiliations:** 1 Department of Immunology and Molecular Biology, Makerere University, Kampala, Uganda; 2 Makerere University/UVRI Centre of Excellence in Infection and Immunity Research and Training (MUII-Plus), Uganda Virus Research Institute (UVRI), Entebbe, Uganda; 3 Centre for Computational Biology, Uganda Christian University, Mukono, Uganda; 4 Epidemiology and Demography Department, KEMRI-Wellcome Trust Research Programme, Kilifi, Kenya; 5 The African Center of Excellence in Bioinformatics and Data Intensive Sciences (ACE), Infectious Diseases Institute, Makerere University, Kampala, Uganda; 6 Department of Arbovirology Emerging and Re-Emerging Infectious Diseases, Uganda Virus Research Institute (UVRI), Entebbe, Uganda; 7 Department of Computer Science, College of Computing, Makerere University, Kampala, Uganda; 8 School of Life Sciences and Zeeman Institute for Systems Biology and Infectious Disease Epidemiology Research (SBIDER), University of Warwick, Coventry, United Kingdom; 9 Microsoft Research, Redmond, Washington 98052 United States; 10 London School of Hygiene and Tropical Medicine (LSHTM), Keppel St, Bloomsbury, London, United Kingdom; 11 Present Address: Oak Ridge Institute for Science and Education, Bioinformatics Research Fellow to the Division of Viral Diseases, Centers for Disease Control and Prevention, Atlanta, Georgia United States

**Keywords:** Evolution, Molecular evolution, Phylogenetics

## Abstract

Genetic characterisation of circulating influenza viruses directs annual vaccine strain selection and mitigation of infection spread. We used next-generation sequencing to locally generate whole genomes from 116 A(H1N1)pdm09 and 118 A(H3N2) positive patient swabs collected across Uganda between 2010 and 2018. We recovered sequences from 92% (215/234) of the swabs, 90% (193/215) of which were whole genomes. The newly-generated sequences were genetically and phylogenetically compared to the WHO-recommended vaccines and other Africa strains sampled since 1994. Uganda strain hemagglutinin (n = 206), neuraminidase (n = 207), and matrix protein (MP, n = 213) sequences had 95.23–99.65%, 95.31–99.79%, and 95.46–100% amino acid similarity to the 2010–2020 season vaccines, respectively, with several mutated hemagglutinin antigenic, receptor binding, and N-linked glycosylation sites. Uganda influenza type-A virus strains sequenced before 2016 clustered uniquely while later strains mixed with other Africa and global strains. We are the first to report novel A(H1N1)pdm09 subclades 6B.1A.3, 6B.1A.5(a,b), and 6B.1A.6 (± T120A) that circulated in Eastern, Western, and Southern Africa in 2017–2019. Africa forms part of the global influenza ecology with high viral genetic diversity, progressive antigenic drift, and local transmissions. For a continent with inadequate health resources and where social distancing is unsustainable, vaccination is the best option. Hence, African stakeholders should prioritise routine genome sequencing and analysis to direct vaccine selection and virus control.

## Introduction

Novel influenza type-A viruses (IAVs) cause human respiratory infections that lead to social lockdowns, economic losses, and millions of deaths^1^. Genomic sequencing and characterisation of circulating IAVs are important to differentiate them from other viruses causing similar clinical symptoms for effective viral control and prevention. Seasonal influenza-related illnesses kill 290,000–650,000 people globally per year, mostly in sub-Saharan Africa^2^. Influenza accounts for 21.7% and 10.1% of the influenza-like illnesses (ILI) and severe acute respiratory illnesses (SARI) in Africa, respectively, and circulates all-year-round with discernible influenza peaks in North and South Africa^3^. Uganda's annual epidemics have two major peaks between May and November and usually constitute multiple IAV types and subtypes responsible for 13% and 6% of the ILI and SARI cases, respectively^4^.

Vaccination and antiviral treatment are the best ways to prevent and control viral transmission^5^. However, the multi-segmented IAVs continuously mutate, especially in the antigenic surface genes, hemagglutinin (HA) and neuraminidase (NA), giving rise to vaccine-escape and drug-resistant viruses^6^. Vaccine formulations for the Northern (NH) and Southern hemispheres (SH) are updated annually to match circulating viruses. Countries select appropriately licensed vaccines based on the genetic relatedness of their circulating viruses to the vaccines^5^.

Well-sampled and resource-rich countries have deep sequenced circulating IAVs using next-generation sequencing (NGS) and utilised advanced bioinformatics and phylogenetic analysis to collect sufficient data on IAV evolution patterns, drug sensitivity^6^, and emerging and circulating viral genetic clades^7^ for virus control and vaccine selection^5^. Influenza surveillance in Africa has improved substantially since the mid-2000s^3,8,9^. However, the limitation in financial resources and advanced phylogenomic analysis capacity restricts the genomic characterisation of IAVs in Africa. We systematically searched 7 databases: African Journals Online, Embase, Global Health, Google Scholar, PubMed, Scopus, and Web of Science for studies on genomic analysis of Africa IAVs. As of 30th July 2021, we found 16% (11/71) of the eligible studies sequenced and/or analyzed whole genomes (WGs) of Africa IAVs, and only 8% (5/62) of the studies that generated new viral sequences used NGS^10^. Only 3 Ugandan studies have sequenced WGs from 59 A(H3N2)^11,12^, and 19 A(H1N1)pdm09^13^ IAV strains sampled in 2008–2009 and 2009–2011, respectively, with sequencing conducted in the United States.

We aimed to explore the feasibility of using next-generation sequencing (NGS) in a resource-limited setting to generate WGs of Uganda IAVs sampled in 2010–2018, and compare the newly-generated sequences with vaccine strains and public Africa IAV sequences sampled in 1994–2019. We analysed the HA carrying antigenic sites which trigger host immune responses and the antiviral targets NA and matrix protein (MP). Further analysis of our newly-generated viruses for which we successfully assembled whole genomes showed evidence of intra-subtype reassortment events and reassortant A(H1N1)pmd09 and A(H3N2) strains circulating in Uganda^14^.

This work birthed an East African network of influenza molecular epidemiologists, which we hope to expand across Africa.

## Results

### Demographic characteristics of sampled patients

**Figure 1:**
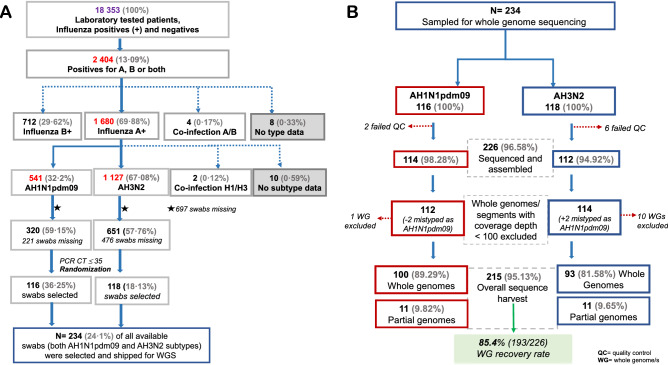
Workflow of swab selection and whole genome recovery. (**A**) Shows how swabs were selected for influenza whole-genome sequencing (WGS). Patients diagnosed with either influenza subtypes A(H1N1)pdm09 or A(H3N2) and whose swab had a PCR CT ≤ 35 had their laboratory codes randomised based on the subtype and year of collection using the R software v3.6.3 (https://www.r-project.org). All available swabs were retrieved for years with less than fifteen swabs. The 697 swabs missing include some shipped to the Centers for Disease Control and Prevention (CDC) for routine surveillance and some lost due to an accidental failure of a freezer. The numbers are based on the UVRI-NIC laboratory dataset only, as of 9th May 2018. (**B**) Shows how viral samples were excluded before and after sequencing and the rate of whole genome recovery. Eight viruses [2 A(H1N1)pdm09 and 6 A(H3N2)] failed quality control (QC) before sequencing.

The Uganda Virus Research Institute National Influenza Centre (UVRI-NIC) laboratory tested 18,353 patients between 22nd October 2010 and 9th May 2018. Thirteen-percent (2404/18,353) were positive for influenza, 69.88% (1680/2404), 29.62% (712/2404), and 0.17% (4/2404) had influenza A, B, and A/B co-infection, respectively (Fig. 1A). IAV positives included 67.08% (1127/1680) A(H3N2), 32.2% (541/1680) A(H1N1)pdm09, and 0.12% (2/1680) AH1/H3 co-infections.

The mean number of swabs sampled per subtype per year was 13 (1–18), excluding 2012, 2016, and 2018 with 2, 1 A(H1N1)pdm09, and no A(H3N2) swab available, respectively (Supplementary Table 1). Three A(H1N1)pdm09 and one A(H3N2)] sampled patients’ swabs lacked demographic data. Of the 230 swabs with data, 65.22% (150/230) and 34.78% (80/230) were from ILI and SARI cases, respectively. The number of sequenced and un-sequenced swabs were not significantly different per case, gender, age group, and geographical region per year, except Central had more swabs sequenced than other regions in 2014 and 2016 (Table 1). The mean age was not significantly different between our study and the UVRI-NIC patients (Supplementary Fig. 1).

**Table 1:** Comparison of demographic characteristics of influenza A positive patients sampled by the general UVRI-NIC surveillance programme whose viral swabs were successfully or not sequenced.

Variable	Subgroup	Total [N]	Successfully Sequenced [*n*%]	Un-sequenced [n%]	P-value
Subtype	H1N1pdm09	**588**	106 [*18.03*]	482 [*81.97*]	< 0.0001*
	H3N2	**1190**	105 [*8.82*]	1085 [*91.18*]	
	H1N1	**29**	0 [*0*]	29 [*100*]	
Case	ILI	**1196**	139 [*11.62*]	1057 [*88.38*]	0.9809
	SARI	**611**	72 [*11.78*]	539 [*88.22*]	
Gender	Male	**951**	120 [12.62]	831 [*87.38*]	0.2149
	Female	**856**	91 [*10.63*]	765 [*89.37*]	
Age [years]	1 month to < 2	**589**	66 [*11.21*]	523 [*88.79*]	0.5851
	2 to < 5	**635**	84 [*13.23*]	551 [*86.77*]	
	5 to < 15	**338**	39 [*11.54*]	299 [*88.46*]	
	15 to < 50	**222**	20 [*9.01*]	202 [*90.99*]	
	50 to < 65	**15**	2 [*13.33*]	13 [*86.67]*	
	≥ 65	**7**	0 [*0*]	7 [*100*]	
	*No age data*	**1**			
Sample origin, by year					
2010	Central	**81**	20 [*24.69*]	61 [*75.31*]	0.5986
	Northwest	**5**	2 [40]	3 [*60*]	
2011	Central	**106**	29 [*27.36*]	77 [*72.64*]	0.5451
	Eastern	**1**	0 [*0*]	1 [*100*]	
	Northwest	**9**	4 [*44.44*]	5 [*55.56*]	
	Western	**8**	1 [*12.5*]	7 [*87.5*]	
2012	Central	**193**	14 [*7.25*]	179 [*92.75*]	0.5272
	Eastern	**18**	0 [0]	18 [*100*]	
	Northwest	**21**	2 [*9.52*]	19 [*90.48*]	
	Western	**10**	1 [10]	9 [*90*]	
2013	Central	**67**	14 [*20.9*]	53 [*79.1*]	0.9346
	Eastern	**3**	1 [*33.33*]	2 [*66.67*]	
	Northwest	**10**	2 [20]	8 [*80*]	
	Western	**4**	1 [25]	3 [*75*]	
2014	Central	**218**	18 [*8.26*]	200 [*91.74*]	0.004*
	Eastern	**46**	0 [*0*]	46 [*100*]	
	Northwest	**35**	1 [*2.86*]	34 [*97.14*]	
	Western	**61**	11 [*18.03*]	50 [*81.97*]	
2015	Central	**192**	18 [*9.38*]	174 [*90.62*]	0.6795
	Eastern	**42**	6 [*14.29*]	36 [*85.71*]	
	Northwest	**19**	2 [*10.53*]	17 [*89.47*]	
	Western	**39**	5 [*12.82*]	34 [*87.18*]	
2016	Central	**139**	13 [*9.25*]	126 [*90.65*]	0.0085*
	Eastern	**6**	3 [*50*]	3 [*50*]	
	Northwest	**15**	0 [*0*]	15 [*100*]	
	Western	**21**	0 [*0*]	21 [*100*]	
2017	Central	**271**	28 [*10.33*]	243 [*89.67*]	0.3299
	Eastern	**22**	0 [*0*]	22 [*100*]	
	Northwest	**11**	0 [0]	11 [*100*]	
	Western	**35**	4 [*11.43*]	31 [*88.57*]	
2018	Central	**33**	11 [*33.33*]	22 [*99.67*]	0.0555

Initially, there were 1800 positives for a single influenza A subtype (H1, H1N1pdm09 and H3N2) in the UVRI-NIC dataset. After excluding one duplicated H1N1pdm09 viral sample (ARU0583), there remained 1799 positive A samples. We included 8 successfully sequenced samples that tested and were reported as positive in the laboratory dataset (used for sample randomization) but were reported as negative in the UVRI-NIC dataset. Therefore, the demographic characteristics presented in this table were for 1807 patients recorded in the UVRI-NIC dataset. 234 of the 1807 patient’s viral swabs were sampled for whole genome sequencing, and 215 of the 234 were successfully sequenced. However, four sequenced viruses (ARU0587, EBB2521, KIB0497, and KSW0631) lacked complete demographic data so we present data for only the 211 viruses that were successfully sequenced with complete data.

Percentage number of sequenced or un-sequenced swabs are in [italics]. P-values showing significant differences between compared groups (< 0.05) are indicated in bold with a star (*).

### Sequencing efficiency

All 234 sampled swabs were analysed, and their mean read counts (ranges) are reported below.

The MiSeq generated 569,435 (806–1,644,430) paired reads per sample (data not shown). Following quality control (QC), 266,020 (70,229–909,340) clean reads per sample were processed using the Iterative Refinement Meta-Assembler (IRMA), 265,868 (70,150–908,946) passed IRMA’s QC, 213,809 (1381–908,234) matched flu references, and 113,164 (777–461,172) paired reads were assembled (Supplementary Fig. 2A).

The number of assembled reads decreased with an increase in gene size. The shortest, MP and non-structural protein (NS), had 25,101 (28–91,585) and 19,341 (41–79,167) assembled reads, respectively. The NA, HA, and nucleoprotein (NP) had 18,653 (74–71,377), 14,466 (35–74,550), and 14,151 (69–74,415) reads assembled, respectively. The polymerase subunits: PA, PB2, and PB1 had 10,465 (31–56,243), 7885 (15–47,444), and 4497 (8–31,354) reads assembled, respectively (Supplementary Fig. 2B).

We successfully sequenced and assembled viral genes from 96.58% (226/234) of the swabs (Fig. 1B). Eleven viral WGs with a depth of coverage < 100 were excluded, leaving 215 viruses. 89.77% (193/215) of these were WGs, spanning 100% and > 96.7% nucleotides in the coding sequences (CDS) and complete genome of A/California/7/2009(H1N1) and A/Perth/16/2009(H3N2) vaccine viruses. Our overall WG recovery rate was 85.4% (193/226). The remaining 10.23% (22/215) viruses had complete CDS for 2–7 genes. Two viruses sampled as A(H1N1)pdm09 matched IRMA’s A(H3N2) references and were included in the A(H3N2) analysis. All newly-generated 215 virus sequences were submitted in a publicly accessible database, GISAID EpiFlu™ (https://www.gisaid.org/), under accessions EPIISL498819–EPIISL498931 [A(H1N1pdm09)], and EPIISL498934–EPIISL499037 [A(H3N2)].

### Antigenic drift among Uganda IAVs

Uganda IAV HA1 proteins continuously drifted away from the 2010–2020 vaccines (Supplementary Table 2). For seasons when formulations differed, Uganda A(H3N2) strains had 1–2 extra unique amino acid (aa) substitutions when compared to the Southern (SH) than the Northern hemisphere (NH) vaccine strains. Since Uganda’s largest part lies north of the equator, the substitutions described below are relative to NH and SNH vaccines (shared by NH and SH) for the sampled 2010–2018 [A(H1N1)pdm09] and 2010–2017 [A(H3N2)] seasons.

We observed 18 unique aa substitutions across the five antigenic sites^15,16^ amongst the 107 A(H1N1)pdm09 strains (Supplementary Table 2A). Ranking from the most variable, the main antigenic sites Ca_2_, Sa, Sb, Ca_1_, and Cb had 6, 5, 4, 2, and 1 unique aa substitutions, respectively. Substitution S164T, S185T, S203T, and H138R and S74R were the most frequent at site Sa, Sb, Ca_1_, and Ca_2_, respectively. All 2010–2016 viruses had S203T, and 90% (27/30) of the 2017–2018 viruses had S164T and S74R.

There were 92 unique aa substitutions across the five antigenic sites^16,17^ amongst the 99 A(H3N2) strains (Supplementary Table 2B). The antigenic sites B, A, D, C, and E had 24, 22, 17, 16, and 13 unique aa substitutions, respectively. Substitution K144N, P194L, H311Q, S96N, and K62E was the most frequent at site A, B, C, D, and E, respectively. Forty-seven percent (47/99) and 41.41% (41/99) of the 2010–2017 strains had V186G and N145S, respectively.

Uganda A(H1N1)pdm09 strains had mutated receptor binding sites (RBS, H138Q/R, S190V, and D222E) and S164T that alter the glycosylation motif at sites 162–164^7^. Uganda A(H3N2) strains had more aa substitutions affecting the RBS [130-loop (T135K, A/S138S/A, I/R140K, R140I), 150-loop (Q/H156H/Q), 190-helix (I192V, P194L, A196T, Q197H/R, A/S/A/P/S198S/A/P/S/P), 220-loop (N/D225D/N, F/Y219S)], and those creating (S45N, A128T, K160T) and removing [N45S, N122S/D, N144K/S, T/N128A, T135K] potential N-linked glycosylation sites (Supplementary Table 2B).

Subgroup analysis showed differences of 1–4 and 1–32 unique aa substitutions at antigenic sites of A(H1N1)pdm09 and A(H3N2) strains, respectively, sampled from different cases, gender, age groups, and geographical regions relative to each subtype vaccines (Supplementary Tables 3, 4).

### Amino acid similarity of complete HA, NA, and MP protein sequences of Uganda IAVs to vaccine strains

The complete HA (H1), NA (N1), and MP protein sequences of Uganda A(H1N1)pdm09 strains had 94, 81, and 21 unique aa substitutions and a mean amino acid similarity of 98.09 (96.99–99.65%), 98.2 (96.8–99.79%), and 99.17 (97.73–100%), respectively, compared to A/California/7/2009(H1N1), A/Michigan/45/2015(H1N1), and A/Brisbane/02/2018(H1N1) vaccines (Supplementary Table 5). All N1 proteins lacked the neuraminidase inhibitors (NAIs) resistance substitution H275Y. However, 7.55% (8/106) had T362I (n = 1), I117M (n = 2), Y155H (n = 2), and V234I (n = 3) associated with reduced susceptibility to NAIs in vitro^18^.

The complete HA (H3), NA (N2), and MP protein sequences of Uganda A(H3N2) strains had 160, 118, and 31 unique aa substitutions, and mean amino acid similarity of 97.47 (95.23–99.29%), 97.64 (95.31–99.79%), and 99 (95.46–100%), respectively, compared to A/Perth/16/2009(H3N2), A/Victoria/361/2011(H3N2), A/Texas/50/2012(H3N2), A/Switzerland/9715293/2013(H3N2), A/Hong Kong/4801/2014(H3N2), A/Singapore/INFIMH-16-0019/2016(H3N2), A/Switzerland/8060/2017(H3N2), A/South Australia/34/2019(H3N2), and A/Kansas/14/2017(H3N2) vaccines (Supplementary Table 6). N2 proteins lacked the NAI-resistance H274Y (N2 numbering), but 18.8% (19/101) carried Y155F (n = 3), and E/D221D/K/E (n = 16) that reduce susceptibility to NAIs^18^.

All A(H1N1)pdm09 and A(H3N2) M2 proteins had the primary adamantine-resistance marker (S31N), and 7.7% (8/104) of A(H3N2) had secondary V27A relative to adamantine-susceptible A/New York/392/2004(H3N2) strain (Supplementary Fig. 3). All Uganda IAVs M2 proteins had aa substitutions (L3I, L4F, T5F, E6K, V7I, E8C, and T9R) fixed in their extracellular N-terminal, a region that supports M2-antibody interactions^19^.

The influenza surveillance webtool (FluSurver) identified aa substitutions in complete HA and NA proteins reported to alter host specificity and cause mild/strong drug resistance, respectively, and aa substitutions in both proteins that could potentially alter viral virulence, antigenic drift, glycosylation, and sites of interactions (Supplementary Tables 5, 6).

### Temporal and spatial divergence of Uganda IAVs

**Figure 2:**
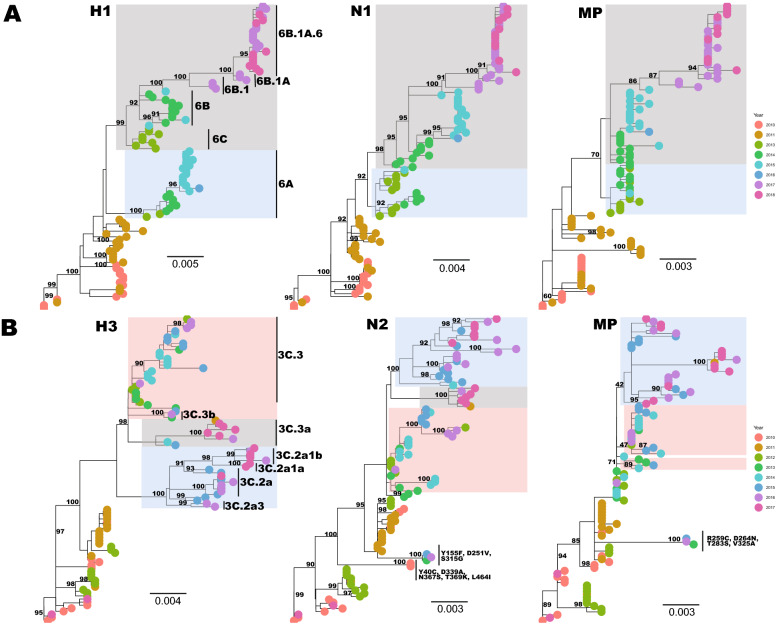
Phylogenies showing the temporal divergence of the HA, NA and MP genes of Uganda A(H1N1)pdm09 (**A**) and A(H3N2) (**B**) influenza viruses sampled from 2010 to 2018. Trees were rooted using the oldest sequence in the dataset. Shaded clusters are the two and three major co-circulating lineages observed since 2013 and 2012 for A(H1N1)pdm09 and A(H3N2) viruses, respectively. The third A(H3N2) lineage (with one 2011 and 2016–2017 viruses) disappeared in the MP phylogeny.

Uganda IAV strains phylogenetically clustered according to their year of sampling, with multiple lineages circulating annually (Fig. 2). Two major H1 lineages co-circulated; lineage 1 (shaded blue) with strains belonging to clade 6A and lineage 2 (shaded grey) with clade 6C, 6B, 6B.1, 6B.1A, 6B.1A.6 strains circulated in 2013–2016 and 2013–2018, respectively (Fig. 2A). The N1 and MP phylogenies showed similar lineages 1 and 2 emerged in 2013 and 2014, respectively, and lineage 2 dominated in 2016–2018.

The dominant H3 lineage 1 (pink) contained clade 3C.3b and 3C.3 strains that circulated from 2013 to 2016. Lineages 2 (grey) had clade 3C.3a and lineage 3 (blue) had clade 3C.2a, 3C.2a3, and 3C.2a1(a, b) strains that emerged in 2014 and 2015, respectively, and co-circulated through 2017. We observed two long-branched clusters (bootstrap = 100%); cluster 1 (KSW0659 and KSW0643, sampled in 2010) and cluster 2 (TOR0492, TOR1664, and NSY0304, sampled in 2013–2016) in the N2 and MP phylogeny, with 3–5 unique amino acid (9–13 nucleotides) substitutions absent in other Uganda strains (Fig. 2B).

Virus strains sampled from different geographical sites mixed in all phylogenies (Supplementary Fig. 4).

### Viral clades circulating in Uganda

**Figure 3:**
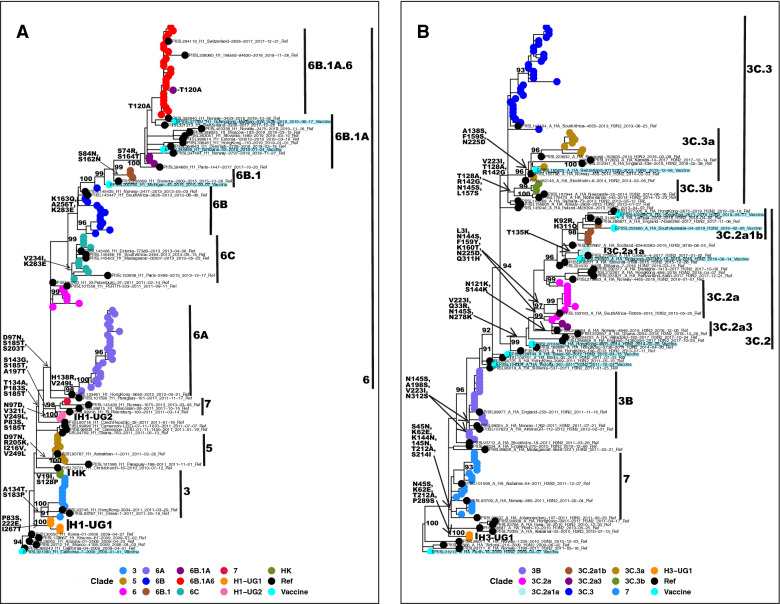
Genetic clades of influenza A viruses that previously circulated in Uganda during the 2010–2018 seasons. All labelled clades (indicated by black bars) were inferred based on the signature amino acid substitutions in the HA1 protein indicated on the tree trunk in bold. (**A**) Shows clades for 2010–2018 A(H1N1)pdm09 viruses. Novel clades H1-UG1 and H1-UG2 are indicated. Genetic clade 6 diverged into 6A, 6B, and 6C. All clade 3, 5, and 7 viruses were collected from Entebbe and Kampala (Central Uganda) and circulated in 2010–2011. (**B**) Shows clades for 2010–2017 A(H3N2) viruses. A novel clade H3-UG1 is indicated. Clade 3 persisted in all 9 years. A similar figure with the full sequence names is provided in Supplementary Fig. 5.

Uganda A(H1N1)pdm09 strains belonged to five global clades (A/Hong Kong/2212/2010(H1N1)-HK, 3, 5, 6, and 7) (Fig. 3A and Supplementary Fig. 5). The HK clade circulated in 2010 and had aa substitutions V19I, N97D, and S128P. Two novel clades H1-UG1 with P83S, D222E, and I267T, and H1-UG2 with T134A, P183S, and S185T, circulated in 2010 and 2011, respectively. Clade 3 had 2010–2011 strains with A134T and S183P. Clade 5 had 2011 strains with D97N, R205K, I216V, and V249L. One 2011 strain from Kisenyi clustered with A/St.Petersburgh/100/2011(H1N1) in clade 7 with A197T, S143G, and K163I. Clade 6 viruses with D97N, S185T, and S203T dominated since 2012 and diverged into 6A, 6B, and 6C. A novel subclade 6B.1A.6 with T120A emerged in November 2017 and dominated through 2018.

Uganda A(H3N2) strains belonged to two global clades 3 and 7 (Fig. 3B) and co-circulated in 2010–2012. A novel clade H3-UG1 with L183H, T212A, S214I, and P289S circulated in 2010. The major clade 3 had strains with N145S and V223I and diverged into 3B, 3C.2, and 3C.3. New subclades 3C.2a1a with T135K and 3C.2a1b with K92R and H311Q dominated in May–June and May–November 2017, respectively.

Based on our dataset, similar clades circulated in Uganda and other African countries, except for A(H1N1)pdm09 clade 7, HK, and H1-UG2 (Supplementary Table 7). However, our recent systematic review showed that clade 7-like strains also circulated in Kenya, Tanzania, and South Africa between 2010 and 2012^10^.

### Phylogenetic relatedness of Africa IAVs

We define a group as a highly-supported phylogenetic cluster (bootstrap ≥ 90%) with at least three Uganda IAV nucleotide sequences.

Uganda A(H1N1)pdm09 strains collected before 2016 clustered uniquely towards the root, while the 2017–2018 strains mixed with Eastern, Central, Western, and Southern Africa strains (Supplementary Figs. 6–8). The H1, N1, and MP phylogeny contained 6, 5, and 1 group, with 50% (3/6), 60% (3/5), and 100% of the groups unique to Uganda, respectively (Supplementary Table 8A).

Uganda A(H3N2) strains collected in 2008–2016 and some before April 2017 clustered uniquely closest to the root (Supplementary Figs. 9–11). Notably, the 2008–2009 Makerere Walter Reed project (MWRP)^11^ and our newly-generated strain sequences clustered separately. The H3, N2, and MP phylogeny had 8, 10, and 2 groups, respectively, and 50% (4/8) H3 (circulated in different years) and 40% (4/10) N2 groups had only Uganda strains (Supplementary Table 8B).

If not clustered alone, Uganda strains grouped with strains from neighboring Kenya, Tanzania, Madagascar, and Congo. Interestingly, four unique H3 lineages (bootstrap ≥ 90%); Kenya (n = 1), Congo (n = 1), and West Africa (n = 2) co-circulated in 2019 (not shown). Virus group details are provided in Supplementary Table 8.

## Discussion

Our study demonstrated the feasibility of NGS whole-genome sequencing of IAVs in a resource-limited setting. Analysis of the newly-generated HA, NA, and MP sequences highlighted a continuous antigenic drift, multiple introductions, and local transmission of A(H1N1)pdm09 and A(H3N2) viruses in Uganda. All Uganda strains lacked the neuraminidase inhibitors resistance marker (H275Y) but had the adamantine-resistance marker (S31N) in their MP proteins. Although we analyzed only the HA, NA, and MP, additional amino acid substitutions such as I38T/F/M in the endonuclease domain of the Polymerase PA protein have also been shown to give rise to antiviral resistance^20–22^. Africa IAVs were genetically similar, but unique viral lineages (bootstrap ≥ 90%) circulated and persisted in Uganda and other countries for 1–3 years.

We successfully recovered 96.58% (226/234) genomes and 85.4% (193/226) WGs directly from frozen human swabs. Our WG recovery rate is comparable to the 82–88% reported in developed countries^23,24^ and better than in Scotland (47.3%)^25^ and Mexico (66%)^26^. 10–20% of frozen swabs fail NGS due to RNA degradation during storage and pre-sequencing analysis. The shortest genes, MP and NS, were sequenced at a greater depth and the least for the polymerase segment 2 (PB1) as previously reported in Mexico and France^23,26^.

Sequence analysis showed most amino acid substitutions affected the antigenic sites Sa and Ca_2_ of A(H1N1)pdm09 and sites A and B of A(H3N2) viruses. Substitutions S164T (Sa) and S74R (Ca_2_) observed in 90% of Uganda A(H1N1)pdm09 strains also dominated in Kenya in 2017–2018^27^. The globally frequent S203T at site Ca_1_ has no known function^28^. Five-percent (4/77) of the 2010–2016 Uganda A(H1N1)pdm09 strains had the RBS D222E, speculated to increase infection severity^28^. Amino acid substitutions in the H1 (P100S, S220T, I338V) and N1 (V106I, N248D) detected earlier in 2009–2011 among Uganda A(H1N1)pdm09 strains relative to A/California/7/2009(H1N1)^13^ persisted through 2017. All 2010–2013 Uganda A(H3N2) strains had mutated antigenic sites also observed in Kenya^29^. It would be interesting to do antigenic and phenotypic analysis to assess the effect of the observed aa substitutions on vaccine responses and viral characteristics (virulence, pathogenicity, and transmissibility), respectively.

Uganda IAV strains lacked the NA H274Y or H275Y substitutions confirming previous reports of 98% of global IAVs being sensitive to NAIs^6^. However, some N1 proteins carried permissive aa substitutions V234I, N369K, and V241I known to counteract the effect of H275Y. These aa substitutions enhance the NA surface expression and enzymatic activity, hence increasing viral fitness. Uganda IAVs NA active and catalytic sites^30^ were highly conserved indicating a lack of pharmacological selection pressure, which confirms the low NAIs intake in Uganda due to their privatization.

Uganda A(H1N1)pdm09 strains formed steeper ladder-like phylogenies than A(H3N2), which could result from sampling effects and/or directional selection due to immune escape^31^. Reassortment (gene exchange) within influenza viruses of the same subtype results in genetically unique strains which also affect the history and structure of the phylogenies^32–34^. Our recent whole-genome analysis using a Bayesian coalescent reassortant constant population model^35^ confirmed reassortment among Uganda A(H1N1)pmd09 (0.1237–0.4255) and A(H3N2) strains (0.00912–0.0355 events/lineage/year)^14^. Further whole-genome analysis to identify additional evolutionary processes driving the observed genetic variations is ongoing.

Phylogenetic analysis revealed the circulation of multiple viral lineages and clades in Uganda as observed worldwide^7,11,36^. New viral lineages and clades were observed in-between seasons indicating multiple viral introductions into Uganda per year. Novel subclades 6B.1A.6 [A(H1N1)pdm09] and 3C.2a1a and 3C. 2a1b [A(H3N2)] emerged in Uganda in 2017. Our detailed review of previous studies showed that clade 3C.3a emerged outside Africa in 2013, and later circulated in Burkina Faso, Ghana, Senegal, South Africa, Ethiopia, Tanzania, Madagascar, Cameroon, and Nigeria in 2014^10^. Here, we show evidence that a clade 3C.3a-like strain (KSY0906_A_HA-H3_Uganda_UVRI_Kisenyi_003_2011-10-19) was first sampled in 2011 and similar strains circulated in 2014 through 2017 in Uganda. While its subclade 3C.3a1 continued to circulate outside Africa through 2021, making it the longest circulating H3 subclade^10,37^. Strikingly, we observed A(H1N1)pdm09 strains belonging to novel subclades 6B.1A.3, 6B.1A.5a, 6B.1A.5b, and 6B.1A.6 (± T120A) sampled during 2018, 2019, 2017–2018, and 2017–2018 seasons, respectively, in Eastern, Western, and Southern Africa (Supplementary Table 7), but were never reported before^10^. Uganda IAV strains from all geographical regions mixed, showing widespread local viral transmissions.

Uganda A(H1N1)pdm09 and A(H3N2) strains collected before 2015 and 2016, respectively, phylogenetically clustered distinctively from most Africa strains which could be due to insufficient viral sequencing in earlier years across Africa. Strains collected later were genetically similar to those from Eastern, Central, Southern, and Western Africa. Notably, all 2010–2018 Uganda strains clustered with global strains highlighting Uganda as part of the global influenza ecology.

The coronavirus disease 2019 (COVID-19) pandemic revealed virus exchange between Uganda and other countries through air and border travel. Currently, we are doing phylogeographic analysis on our newly-generated and public global IAV genomes to identify origins of viruses migrating to Uganda and Africa, and the continent's contribution to the global influenza migration network. 

This is the first and largest study to sequence Uganda A(H1N1)pdm09 and A(H3N2) virus whole genomes (WGs) locally, and extensively describe the genetic diversity and evolution patterns of Africa IAVs since 1994. Our work expands on previous influenza sequencing and characterisation efforts of African researchers^10^ and highlights the feasibility of next-generation whole-genome sequencing for real-time disease surveillance and detection of emerging strains in Africa. Our newly-generated sequences add significantly to public Africa IAV genomes. However, we sequenced pre-collected swabs with a biased geographical sampling. Therefore, we did not consider geographical location in the swab randomisation.

Due to financial constraints, we sampled only 24.1% (234/971) of available swabs, 8.12% (19/234) of which failed quality control and NGS. Although we generated WGs, we analysed only the HA, NA, and MP genes from a few strains sequenced per case, gender, age group, and geographical region per year. Therefore, our results do not give a comprehensive description of the genetic diversity of Uganda and Africa IAVs.

Although NGS technologies can improve influenza surveillance in Africa, their implementation presents several challenges in analysing, reporting, and management of their big data. Fortunately, African researchers can leverage freely available automated pipelines^38–40^ for NGS data quality control, assembly, and variant calling for both segmented and non-segmented viruses. However, these pipelines are validated using sequences sampled from outside Africa. As shown here, genomic differences among strains sampled from different geographical regions and times cannot be ignored, hence African researchers will need to strengthen their existing Bioinformatics capacity to customize and/or develop region-specific tools.

Our study provides a platform for larger studies and highlights the potential of molecular surveillance to improve viral detection and disease management. Existing African surveillance programmes should prioritize routine sequencing and genome analysis to monitor circulating IAVs. Our findings will inform Uganda’s public health, use of NAIs prophylactic treatment, and decision to design vaccination programs, especially for high-risk groups like children, pregnant mothers, and the elderly.

## Methods

### Influenza surveillance design and source of swabs

The Uganda Virus Research Institute National Influenza Centre (UVRI-NIC) in Entebbe implements a clinic and hospital-based surveillance at thirteen peri-urban and densely populated sites in seven districts across Northwest, Western, Central, and Eastern Uganda (Supplementary Fig. 12)^4^.

Nasal and oropharyngeal swabs and demographic data were collected from ILI and SARI patients in 2010–2018^4^. The swabs were tested, typed for influenza A and B, and the IAV positives subtyped for seasonal [A(H1N1) and A(H3N2)] and pandemic A(H1N1)pdm09 influenza using the Centers for Disease Control and Prevention’s (CDC) real-time reverse-transcription polymerase chain reaction (rRT-PCR) protocols and primers (Atlanta, Georgia)^41^.

### Swab selection for whole-genome sequencing

We aimed to sequence 100 whole genomes (WGs) per subtype [A(H1N1)pdm09 and A(H3N2)] due to financial constraints. Available swabs from 971 IAV patients with real-time PCR cycle threshold (CT ≤ 35) were stratified by subtype and year of collection, randomised, and then selected every first fifteen swabs per strata. Twenty-four percent (234/971) of the swabs [116 A(H1N1)pdm09 and 118 A(H3N2)] were sequenced at a KEMRI-Wellcome Trust Programme collaborating laboratory in Kilifi, Kenya.

### Viral RNA isolation and amplification

Viral ribonucleic acid (RNA) was extracted from 140 μL swab sample using the QIAamp Viral RNA Mini extraction kit and manufacturer’s protocol (Qiagen, Hilden, Germany). The RNA was reverse transcribed and the whole genome amplified using the multi-segment real-time polymerase chain reaction (M-RTPCR)^24^ and universal IAV Uni/Inf primers at standardised thermocycling conditions (Supplementary methods)^36^.

### Next-generation sequencing

The M-RTPCR amplicon libraries were prepared using the Nextera XT DNA library preparation kit and protocol (Illumina, San Diego, California, USA), cleaned using the 0.8 × AMPure XP beads, quantified on the Qubit 3.0 fluorometer using the dsDNA High sensitivity kit (Invitrogen, Carlsbad, California, USA). Library size distributions were assessed using the Agilent Technology 2100 Bioanalyzer and the High Sensitivity DNA kit (Agilent Technologies, Santa Clara, California, USA). Samples with a broad fragment size spectrum (> 250 bp) were normalized manually to 2 nM. 5 μL per sample library were pooled, denatured using Sodium Hydroxide (NaOH), and diluted to 12.5 pmol. Diluted libraries were spiked with 5% Phi-X control (Illumina, San Diego, CA, USA) and sequenced using the Illumina MiSeq (Illumina Inc., San Diego, California, USA) generating 2 × 250 bp paired reads per sample.

We assessed sequencing efficiency based on the depth of coverage and number of gene segments recovered per swab.

### Sequence quality control and assembly

Raw reads were de-duplicated and decontaminated, and clean reads assembled using the reference-based FLU module of the Iterative Refinement Meta-Assembler (IRMA) v0.6.7^42^ at default settings (Supplementary methods). The A(H1N1)pdm09 and A(H3N2) virus assemblies were compared with A/California/7/2009(H1N1) and A/Perth/16/2009(H3N2) vaccine viruses, respectively.

The newly-generated sequences were deposited in the Global Initiative on Sharing All Influenza Data database (GISAID EpiFlu™, https://www.gisaid.org/) under accessions EPIISL498819-EPIISL498931 [A(H1N1)pdm09] and EPIISL498934-EPIISL499037 [A(H3N2)].

### Genetic characterisation of surface and matrix proteins

Gene sequences for the Southern and Northern Hemispheres or both (SNH) vaccine and clade reference viruses per subtype were downloaded from the GISAID EpiFlu database^43^ (https://www.gisaid.org/; accessed on 26th February 2020). Uganda and vaccine virus sequences per subtype were aligned using MUSCLE v3.8.1551^44^.

Amino acid substitutions in the major antigenic sites (A, B, C, D, and E) of A(H1N1)pdm09^15^ and A(H3N2)^16^ virus HA1 proteins were identified manually, while those in the complete HA, NA, and MP proteins and their functions were identified using the influenza surveillance (FluSurver) webtool (http://flusurver.bii.a-star.edu.sg; accessed on 24th September 2021) (Supplementary methods).

### Phylogenetic analysis and clade classification

Uganda HA, NA, and MP gene sequences per subtype were aligned and maximum-likelihood trees reconstructed using IQtree v1.6.11^45^ with a GTR + G4 model and 1000 bootstraps. Trees were rooted using the oldest sequence in the dataset and visualized in ggtree v2.4.1^46^ and Figtree v1.4.4 (http://tree.bio.ed.ac.uk/software/figtree/).

Uganda, clade references, and vaccine virus sequences per subtype were aligned and maximum-likelihood trees reconstructed as above. Viral sequences were classified into genetic clades based on signature amino acid substitutions in their HA protein HA1 subunits^7^.

For Africa analysis, HA, NA, and MP gene sequences were downloaded from GISAID, accessed on 27th February 2020. Sequences with > 100 bps shorter or longer than the actual gene size and ambiguous “N” bases were excluded. The remaining A(H1N1)pdm09 (496 H1, 443 N1, and 278 MP) and A(H3N2) (718 H3, 675 N2, and 439 MP) sequences were aligned using a codon-aware aligner (https://github.com/veg/hyphy-analyses/tree/master/codon-msa) and maximum-likelihood trees reconstructed as above. Accessions for sequences analysed are provided in Supplementary Table 9.

### Statistical analysis

We used the Chi-Square or Fisher’s Exact test (as appropriate) and the Wilcoxon rank-sum test in R v3.6.3 (https://www.r-project.org), to compare differences in the categorical and continuous patient demographics between successfully sequenced and un-sequenced swabs, respectively.

### Ethics

This study was approved by the Makerere University School of Biomedical Sciences Research and Ethics Committee (SBS-REC) (ref: SBS-577) and the Uganda National Council of Science and Technology (UNCST) (ref: HS2519).

Our study was nested in the National Influenza Surveillance Programme at the Uganda Virus Research Institute (UVRI) where patients’ consent and assent were verbally obtained before sample collection. Since our study used archived patient clinical samples collected from the surveillance programme and presented “minimal risk” of harm to patients, we obtained a waiver of consent through the Makerere University SBS-REC. Permission to use archived samples was granted by Prof. Julius Lutwama, director of the Department of Arbovirology and Emerging and Re-Emerging Viral Infections at UVRI.

## Supplementary Information

Supplementary Information.

## Data Availability

All sequences generated in this study with their respective metadata were submitted to GISAID EpiFlu (https://www.gisaid.org/) under accessions EPIISL498819–EPIISL498931 [A(H1N1pdm09)], and EPIISL498934–EPIISL499037 [A(H3N2)].
