# Supplementary material for: Phylogenomic analysis uncovers a 9-year variation of Uganda influenza type-A strains from the WHO-recommended vaccines and other Africa strains
Source: Sci Rep. 2023 Apr 4;13:5516. doi: 10.1038/s41598-023-30667-z (PMC10072032; doi:10.1038/s41598-023-30667-z)
Supplement: Supplementary file 1 — Supplementary Information. [file 41598_2023_30667_MOESM1_ESM.pdf]

Table of Contents

Supplementary results 3

Supplementary Table 1: Number of influenza A positive patients' swabs that were available for sampling per subtype 3

Supplementary Fig. 1: Age comparison between the general UVRI-NIC surveillance programme, excluded, and sampled influenza A patients 4

Supplementary Fig. 2: Metrics for clean MiSeq reads used for sequence assembly 5

Supplementary Table 2: Antigenic drift among Uganda A(H1N1)pdm09 and A(H3N2) influenza viruses 6

Supplementary Table 3: Antigenic drift observed among Uganda A(H1N1)pdm09 viruses per case, gender, age group, and geographical region per year 8

Supplementary Table 4: Antigenic drift observed among Uganda A(H3N2) viruses per case, gender, age group, and geographical region per year 10

Supplementary Table 4 (continued): Antigenic drift observed among Uganda A(H3N2) viruses per case, gender, age group, and geographical region per year 13

Supplementary Table 5: Amino acid substitutions (AASs) in the complete HA, NA, and MP protein sequences of Uganda A(H1N1)pdm09 viruses compared to Southern and Northern Hemisphere vaccines viruses 18

Supplementary Table 6: Amino acid substitutions (AASs) in the complete HA, NA, and MP protein sequences of Uganda A(H3N2) viruses compared to Southern and Northern Hemisphere vaccines viruses 21

Supplementary Fig. 3: Multiple sequence alignment (MSA) of Uganda A(H3N2) M2 protein sequences with adamantine-susceptible A/New York/392/2004(H3N2) 27

Supplementary Fig. 4: Phylogenies showing the spatial divergence Uganda influenza A viruses 28

Supplementary Fig. 5: Genetic clades of influenza A viruses that previously circulated in 2010-2018 in Uganda 29

Supplementary Table 7: Details of influenza A viral genetic clades that circulated in Africa from 1994 to 2019 31

Supplementary Fig. 6: Phylogenetic clustering and clades of Uganda and other Africa A(H1N1)pdm09 viruses in the hemagglutinin gene 33

Supplementary Fig. 7: Phylogenetic clustering of Uganda and other Africa A(H1N1)pdm09 viruses in the neuraminidase gene 34

Supplementary Fig. 8: Phylogenetic clustering of Uganda and other Africa A(H1N1)pdm09 viruses in the matrix protein gene 35

Supplementary Fig. 9: Phylogenetic clustering and clades of Uganda and other Africa A(H3N2) viruses in the hemagglutinin gene 36

Supplementary Fig. 10: Phylogenetic clustering of Uganda and other Africa A(H3N2) viruses in the neuraminidase gene 37

Supplementary Fig. 11: Phylogenetic clustering of Uganda and other Africa A(H3N2) viruses in the matrix protein gene 38

Supplementary Table 8: Details of highly supported phylogenetic groups among the newly-generated Uganda viruses and other African viruses 39

Supplementary materials and methods 40
Source of swabs and sampling 40
Viral RNA isolation and amplification 40
Next-generation sequencing 41
Sequence quality control 41
Genome assembly using Iterative Refinement Meta-Assembler (IRMA) 41
Identification of amino acid substitutions and their biological relevance across the complete HA, NA, and MP proteins of Uganda IAVs using the influenza surveillance (FluSurver) webtool 42
Supplementary Fig. 12: Geographical distribution of sampled sentinel sites in the general UVRI-NIC influenza surveillance programme 44
Supplementary Table 9: Accession numbers for the Africa influenza, clade references, and vaccine viral sequences analysed per subtype 45
References 47

Supplementary results

Supplementary Table 1: Number of influenza A positive patients' swabs that were available for sampling per subtype

Year	Total number of swabs from A(H1N1)pdm09 positives	Number of swabs available for sampling (no PCR criteria)	Number of swabs available for sampling PCR flu a subtype (CT≤35)	Number of swabs randomly sampled	Total number of swabs from A(H3N2) positives	Number of swabs available for sampling (no PCR criteria)	Number of swabs available for sampling PCR flu a subtype (CT≤35)	Number of swabs randomly sampled
	**A(H1N1)pdm09**				**A(H3N2)**			
2010	18	14	14	14	19	13	13	12
2011	93	71	67	19	51	45	44	16
2012	4	3	3	2	223	76	72	15
2013	71	23	22	15	30	19	19	15
2014	145	25	25	18	211	16	16	12
2015	144	130	130	17	118	100	100	14
2016	1	1	1	1	191	174	174	17
2017	51	42	42	19	284	208	206	17
2018	14	11	11	11	0	0	0	0
**Total**	**541**	**320**	**315**	**116**	**1127**	**651**	**644**	**118**

Supplementary Table 1: The number of IAV positive patient swabs that were available and randomly selected for WGS per subtype per year (2010-2018). For years where the number of available swabs was less than 15, all available swabs were sampled. Following the first randomisation, 5 A(H1N1)pdm09 and 13 A(H3N2) swabs recorded as available could not be located in the storage freezers. A second randomisation (not based on year) was done excluding swabs from the first randomisation that had successfully been retrieved. The first 5 swabs for the A(H1N1)pdm09 and 13 swabs for the A(H3N2) were chosen bringing the total to 116 and 118, respectively. All numbers are based on the UVRI-NIC laboratory dataset sampled between 22nd October 2010 and 9th May 2018.

Supplementary Fig. 1: Age comparison between the general UVRI-NIC surveillance programme, excluded, and sampled influenza A patients

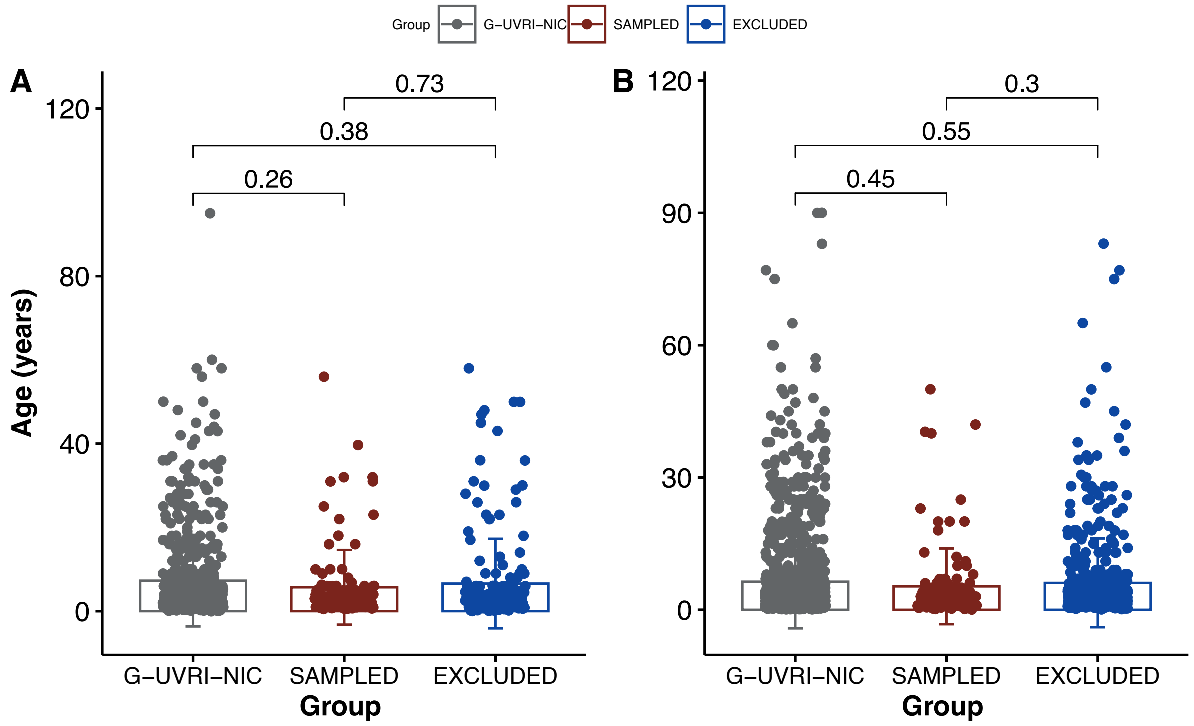

Supplementary Fig. 1: Age comparison between the general UVRI-NIC surveillance programme, excluded, and sampled influenza A patients. Group definitions: G-UVRI-NIC are all IAV patients recorded in general UVRI-NIC surveillance database (N=1800), SAMPLED are IAV patients whose swabs were sampled in this study, and EXCLUDED are IAV patients whose swabs were not sequenced due to financial constraints. Group comparisons were done using the Wilcoxon rank sum test at p-value threshold 0.05. **Panel A** shows ages for A(H1N1)pdm09 patients and **Panel B** shows ages for A(H3N2) patients. 20 excluded patients' swabs (9 H1N1pdm09 and 11 H3N2) had no demographic data.

Supplementary Fig. 2: Metrics for clean MiSeq reads used for sequence assembly

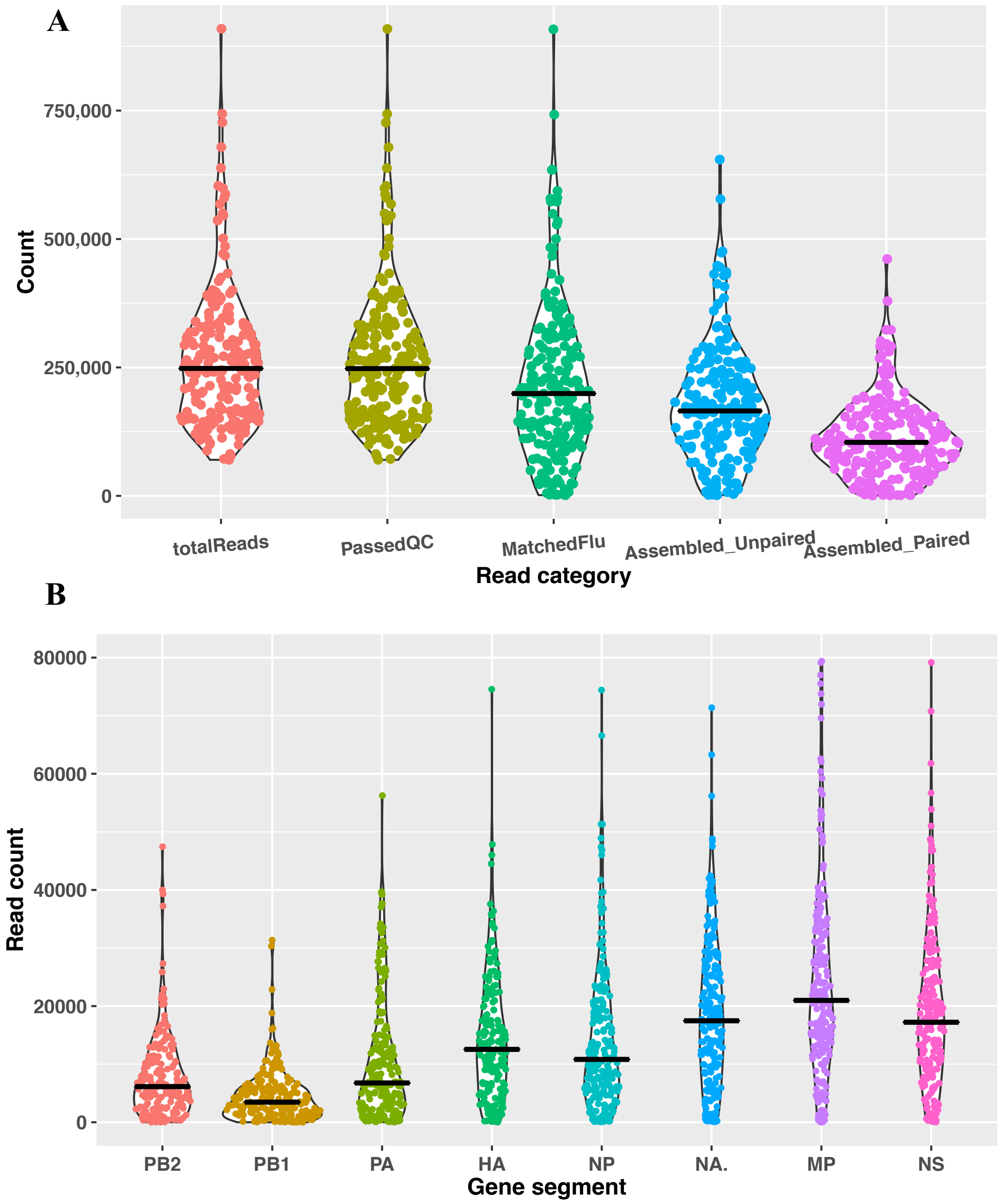

Supplementary Fig. 2: Metrics for clean MiSeq reads used for sequence assembly using IRMA Flu module. **Panel A shows** the number of reads in each read category obtained per viral swab sample. **totalReads** is the number of reads used as input for IRMA. PassedQC are the reads that passed IRMA internal quality control (QC). **MatchedFlu** are reads that matched IRMA's flu references. **Assembled_Paired** are the number of paired reads used for the final assemblies, and **Assembled_Unpaired** are the number of unpaired reads used for the final assemblies. **Panel B** shows the number of paired reads used in the final assembly for each of the eight gene segments: polymerase subunits (PB2, PB1 and PA), hemagglutinin (HA), nucleoprotein (NP), neuraminidase (NA), matrix protein (MP) and non-structural protein (NS). Read metrics are for the 215 A(H1N1)pdm09 and A(H3N2) viruses retained after excluding segments with low transverse coverage depth (<100).

Supplementary Table 2: Antigenic drift among Uganda A(H1N1)pdm09 and A(H3N2) influenza viruses

Season	Vaccine: Passage, Genetic clade (Hemisphere)	No. of Uganda viruses analysed *(year sampled)*	Amino acid substitutions	Season	Vaccine: Passage, Genetic clade (Hemisphere)	No. of Uganda viruses analysed *(year sampled)*	Amino acid substitutions
**A**	**A(H1N1)pdm09 viruses**						
2010 - 2016	**A/California/7/2009:** E2E6, Clade 1 (SNH)	77 *(2010 - 2016)*	N125D[a] (1), 156N[a] (77), K163I[a] (5), K163Q[a] (12), S185T[b] (54), A186T[b] (1), S190V[b] (23), S203T[c] (77), R205K[c] (9), H138Q[d] (1), H138R[d] (24), 222D[d] (73), D222E[d] (4), A73T[e] (1)	2020	**A/Brisbane/02/2018**: E3/E2, Clade 6B.1A.1 (SH)	11 *(2018)*	**-**
2017-2018	**A/Michigan/45/2015:** E1, Clade 6B.1 (SNH)	30 *(2017- 2018)*	S164T[a] (27), 191L[b] (30), S74R[d] (27), K142R[d] (1),		**A/Guangdong-Maonan/SWL1536/2019**: E1, clade 6B.1A.5a (NH)	11 *(2018)*	I185T[b] (11), A187D[b] (11), E189Q[b] (11)
2019	**A/Michigan/45/2015**: E1, Clade 6B.1 (SH)	11 *(2018)*	S164T[a] (11), 191L[b] (11), S74R[d] (11)				
	**A/Brisbane/02/2018**: E3/E2, Clade 6B.1A.1(NH)		**-**				
**B**	**A(H3N2) viruses**						
2010-2011	**A/Perth/16/2009**: E3, A/Perth/16 clade (SNH)	25 *(2010 - 2011)*	A138S[a] (1), I140K[a] (1), R142G[a] (1), K144N[a] (25), N145S[a] (16), T128A[b] (1), F159S[b] (1), I192V[b] (3), A196T[b] (1), A198S[b] (15), A198P[b] (1), S44N[c] (1), S45N[c] (6), T48I[c] (1), D53N[c] (1), G275S[c] (2), N278K[c] (1), N312S[c] (15), T212A[d] (25), S214I[d] (25), N225D[d] (1), I242V[d] (1), K62E[e] (25), K83E[e] (1)	2016- 2017	**A/Hong Kong/4801/2014**: E5/E3, clade **3C.2a** (SNH)	27 *(2016- 2017)*	N122D[a] (3), S124N[a] (1), A138S[a] (6), I140K[a] (9), R142G[a] (12), R142K[a] (5), S144N[a] (13), S144K[a] (2), S145N[a] (2), S146G[a] (1), T128A[b] (11), T135K[b] (2), H156Q[b] (1), L157S[b] (1), Y159F[b] (7), Y159S[b] (6), K160T[b] (13), P194L[b] (27), A196T[b] (2), Q197R[b] (5), S198A[b] (2), S198P[b] (7), I48T[c] (2), D53E[c] (2), D53N[c] (7), G275S[c] (1), K276R[c] (1), K278N[c] (2), H311Q[c] (18), S312N[c] (8), S96N[d] (26), N121K[d] (9), N171K[d] (7), 203T[d] (27), K207Q[d] (1), I214T[d] (3), D225N[d] (7), E62G[e] (1), E62K[e] (1), Q75H[e] (2), G78D[e] (2), K83E[e] (1), S91N[e] (1), K92R[e] (5), Y94H[e] (2), S262N[e] (1)
2012	**A/Perth/16/2009**: E3, A/Perth/16 clade (SH)	16 *(2012)*	S124N[a] (1), I140K[a] (1), R142G[a] (5), **K144N[a] (16),** N145S[a] (8), T128A[b] (5), **A198S[b] (8),** S45N[c] (13), T48I[c] (5), N278K[c] (5), N312S[c] (8), N121K[d] (1), **T212A[d] (16),** S214V[d] (3), **S214I[d] (13),** I242V[d] (1), **K62E[e] (16),** S262N[e] (3)	2018	**A/Singapore/INFIMH-16-0019/2016**: E5, Clade 3C.2a1 (SNH)	13 *(2017)*	N122D[a] (2), A138S[a] (5), G140R[a] (1), I140K[a] (6), G142R[a] (6), S144N[a] (7), S145N[a] (2), S146G[a] (1), T128A[b] (5), T135K[b] (2), H156Q[b] (1), Y159F[b] (2), Y159S[b] (5), K160T[b] (5), P194L[b] (13), A196T[b] (2), S198A[b] (2), Q197R[b] (1), S198P[b] (5), I48T[c] (2), D53E[c] (1), D53N[c] (2), G275S[c] (1), K276R[c] (1), K278N[c] (2), H311Q[c] (10), S312N[c] (7), K121N[d] (8), K171N[d] (8), I214T[d] (2), 225N[d] (2), 225D[d] (11), Q75H[e] (1), G78D[e] (2), K83E[e] (1), K92R[e] (3), Y94H[e] (2)

	**A/Victoria/361/2011**: E3/E3, Clade 3C.1 (NH)		S124N^{a} (1), I140K^{a} (1), R142G^{a} (5), N145S^{a} (8), T128A^{b} (5), **Q156H^{b} (16), V186G^{b} (16), S198A^{b} (8)**, N45S^{c} (3), I48T^{c} (11), N278K^{c} (5), S312N^{c} (8), N121K^{d} (1), I214V^{d} (3**), Y219S^{d} (16**), I242V^{d} (1), S262N^{e} (3)	2019	**A/Switzerland/8060/2017** : E5/E2, Clade 3C.2a2 (SH)	13 *(2017)*	N122D^{a} (2), **K131T^{a} (13),** A138S^{a} (5), I140K^{a} (5), **K142R^{a} (8), K142G^{a} (5), S144N^{a} (7),** S145N^{a} (2), S146G^{a} (1), **T128A^{b} (5),** T135K^{b} (2), H156Q^{b} (1), Y159F^{b} (2), **Y159S^{b} (5),** K160T^{b} (6), P194L^{b} (13), A196T^{b} (2), Q197R^{b} (1), S198A^{b} (2), S198P^{b} (5), I48T^{c} (2), D53E^{c} (1), D53N^{c} (2), G275S^{c} (1), K276R^{c} (1), K278N^{c} (2), 300I^{c} (13), **H311Q^{c} (10**), S312N^{c} (7), **S96N^{d} (13),** N121K^{d} (5), N171K^{d} (5), I214T^{d} (2), D225N^{d} (2), Q75H^{e} (1), G78D^{e} (2), K83E^{e} (1), K92R^{e} (3), Y94H^{e} (2), **Q261R^{e} (13)**
2013	**A/Victoria/361/2011**: E3/E3, Clade 3C.1 (SH)	6 *(2013)*	N122S^{a} (1), S124N^{a} (1), I140K^{a} (1), R142G^{a} (6), N145S^{a} (6), **T128A^{b} (6), Q156H^{b} (6),** L157S^{b} (1), V186G^{b} (6), Q197H^{b} (1), D53N^{c} (1), **N278K^{c} (6),** V297I^{c} (1), I214T^{d} (1), **Y219S^{d} (6),** S91G^{e} (1)		**A/Kansas/14/2017**: E5, Clade 3C.3a (NH)		N122D^{a} (2), S138A^{a} (8), I140K^{a} (5), **G142R^{a} (7), G142K^{a} (1), K144S^{a} (6), K144N^{a} (7),** S145N^{a} (2), S146G^{a} (1), **A128T^{b} (8),** T135K^{b} (2), H156Q^{b} (1), S159F^{b} (2), **S159Y^{b} (6),** K160T^{b} (6), **N190D^{b} (6), S193F^{b} (13),** A196T^{b} (2), Q197R^{b} (1), S198A^{b} (2), S198P^{b} (5), I48T^{c} (2), D53E^{c} (1), D53N^{c} (2), G275S^{c} (1), K276R^{c} (1), K278N^{c} (2), **Q311H^{c} (3),** S312N^{c} (7), N121K^{d} (5), N171K^{d} (5), I214T^{d} (2), D225N^{d} (2), **246N^{d} (13**), Q75H^{e} (1), G78D^{e} (2), K83E^{e} (1), **N91S^{e} (13),** K92R^{e} (3), Y94H^{e} (2)
	**A/Texas/50/2012**: E5, Clade 3C.1 (NH)		N122S^{a} (1), S124N^{a} (1), I140K^{a} (1), R142G^{a} (6), N145S^{a} (6), **N128A^{b} (6**), L157S^{b} (1), V186G^{b} (6), Q197H^{b} (1), **P198S^{b} (6),** D53N^{c} (1), V297I^{c} (1), I214T^{d} (1), **F219S^{d} (6),** S91G^{e} (1)	2020	**A/South Australia/34/2019**: E5, Clade 3C.2a1b (SH)	13 *(2017)*	N122D^{a} (2), **K131T^{a} (13),** A138S^{a} (5), I140K^{a} (5), G142R^{a} (7), G142K^{a} (1), S144N^{a} (7), S145N^{a} (2), S146G^{a} (1), **T128A^{b} (5**), **T135K^{b} (2),** H156Q^{b} (1), Y159F^{b} (2), Y159S^{b} (5), K160T^{b} (6), **I186G^{b} (13),** A196T^{b} (2), Q197R^{b} (1), S198A^{b} (2), S198P^{b} (5), I48T^{c} (2), D53E^{c} (1), D53N^{c} (2), G275S^{c} (1), K276R^{c} (1), K278N^{c} (2), Q311H^{c} (3), S312N^{c} (7), K121N^{d} (8), K171N^{d} (8), I214T^{d} (2), **F219S^{d} (13), D225N^{d} (2),** G62E^{e} (13), Q75H^{e} (1), G78D^{e} (2), K83E^{e} (1), R92K^{e} (10), Y94H^{e} (2)
2014	**A/Texas/50/2012**: E5, Clade 3C.1 (SNH)	11 *(2014)*	A138S^{a} (1), I140K^{a} (6), R142G^{a} (11), N145S^{a} (11), N128A^{b} (11), F159S^{b} (1), V186G^{b} (11), P198S^{b} (10), F219S^{d} (11), N121D^{d} (1), N225D^{d} (1), E62K^{e} (1)		**A/Hong Kong/2671/2019**: E7, Clade 3C.2a1b (NH)		N122D^{a} (2), **F137S^{a} (13),** S138A^{a} (8), I140K^{a} (5), G142R^{a} (7), G142K^{a} (1), S144N^{a} (7), S145N^{a} (2), S146G^{a} (1), **A128T^{b} (8**), **K135T^{b} (11),** H156Q^{b} (1), Y159F^{b} (2), Y159S^{b} (5), I160T^{b} (6), **I160K^{b} (7), V186G^{b} (13), S193F^{b} (13),** A196T^{b} (2), Q197R^{b} (1), S198A^{b} (2), S198P^{b} (5), I48T^{c} (2), D53E^{c} (1), D53N^{c} (2), G275S^{c} (1), K276R^{c} (1), K278N^{c} (2), Q311H^{c} (3), S312N^{c} (7), K121N^{d} (8), K171N^{d} (8), I214T^{d} (2), **N225D^{d} (11),** G62E^{e} (13), Q75H^{e} (1), G78D^{e} (2), K83E^{e} (1), R92K^{e} (10), Y94H^{e} (2)
2015	**A/Switzerland/9715293/2013**: E4/E2, clade 3C.3a (SNH)	14 *(2015)*	S124N^{a} (1), S124R^{a} (1), S138A^{a} (13), R140K^{a} (5), R140I^{a} (7), G142K^{a} (2), G142R^{a} (4), N144K^{a} (1), N144S^{a} (6), M168V^{a} (2), A128T^{b} (6), L157S^{b} (1), S159F^{b} (7), S159Y^{b} (6), K160T^{b} (6), V186G^{b} (14), Q197R^{b} (2), D53N^{c} (3), Q311H^{c} (6), N171H^{d} (1), 219S^{d} (14), D225N^{d} (7), V88I^{e} (1), S91N^{e} (1), S262N^{e} (1), S265G^{e} (1)				

SH= Southern hemisphere vaccine. NH= Northern hemisphere vaccine. SNH= vaccine shared for both Southern and Northern hemispheres. A(H1N1)pdm09 HA1 antigenic sites are Sa=a, Sb=b, Ca$_1$=c, Ca$_2$=d, Cb= e as described by Caton et al., 1982. A(H3N2) HA1 antigenic sites are A=a, B=b, C=c, D=d, and E=e as described by Wiley and Skehel, 1987. The frequency of an amino acid substitution observed at a given antigenic site in Uganda HA1 proteins is reported as (n). The substitutions in **BOLD** are unique relative to the SH and NH vaccines of that given year. Mutations against the NH vaccine strains are coloured brown for visualization purposes only. Recommended vaccines for each influenza season are adopted from the World Health Organization (WHO, https://www.who.int/teams/global-influenza-programme/vaccines/who-recommendations).

Supplementary Table 2: Amino acid substitutions in the HA1 antigenic sites of Uganda A(H1N1)pdm09 and A(H3N2) viruses compared to the Southern and Northern vaccine viruses. This table summarises data in the following Supplementary Tables 3-4.

Supplementary Table 3: Antigenic drift observed among Uganda A(H1N1)pdm09 viruses per case, gender, age group, and geographical region per year

Season		2010-2016 (n=77)	2017-2018 (n=30)	2019 (Only 2018 viruses analysed, n=11)		2020 (Only 2018 viruses analysed, n=11)	
**Vaccine**		**A/California/7/2009: E2E6, Clade 1 (SNH)**	**A/Michigan/45/2015: E1, Clade 6B.1 (SNH)**	**A/Michigan/45/2015: E1, Clade 6B.1 (SH)**	**A/Brisbane/02/2018: E3/E2, Clade 6B.1A.1(NH)**	**A/Brisbane/02/2018: E3/E2, Clade 6B.1A.1 (SH)**	**A/Guangdong-Maonan/SWL1536/2019: E1, clade 6B.1A.5a (NH)**
**Antigenic drift by case**							
Case	ILI (N=67)	156N[a] (46), K163I[a] (2), K163Q[a] (7), S185T[b] (29), **A186T[b] (1),** S190V[b] (7), S203T[c] (46), R205K[c] (7), **H138Q[d] (1),** H138R[d] (7), 222D[d] (46)	S164T[a] (21), 191L[b] (21), **K142R[d] (1),** S74R[d] (21)	S164T[a] (10), 191L[b] (10), S74R[d] (10)	-	-	I185T[b] (10), A187D[b] (10), E189Q[b] (10)
	SARI (N=37)	**N125D[a] (1),**156N[a] (29), K163I[a] (3), K163Q[a] (5), S185T[b] (25), S190V[b] (16), S203T[c] (29), R205K[c] (2), H138R[d] (17), 222D[d] (26), **D222E[d] (3), A73T[e] (1)**	S164T[a] (5), 191L[b] (8), S74R[d] (5)	S164T[a] (1), 191L[b] (1), S74R[d] (1)	-	-	I185T[b] (1), A187D[b] (1), E189Q[b] (1)
	No data (N=3)	156N[a] (2), S203T[c] (2), 222D[d] (1), D222E[d] (1)	S164T[a] (1), 191L[b] (1), S74R[d] (1)	-	-	-	-
	HA not recovered (N=9)	-	-	-	-	-	-
**Antigenic drift by gender**							
Gender	Male (N=58)	**N125D[a] (1),** 156N[a] (44), K163I[a] (1), K163Q[a] (7), S185T[b] (30), **A186T[b] (1),** S190V[b] (12), S203T[c] (44), R205K[c] (6), **H138Q[d] (1),** H138R[d] (12), 222D[d] (43), D222E[d] (1)	S164T[a] (13), 191L[b] (14), **K142R[d] (1),** S74R[d] (13)	S164T[a] (6), 191L[b] (6), S74R[d] (6)	-	-	I185T[b] (6), A187D[b] (6), E189Q[b] (6)
	Female (n=46)	156N[a] (31), K163I[a] (4), K163Q[a] (5), S185T[b] (24), S190V[b] (11), S203T[c] (31), R205K[c] (3), H138R[d] (12), 222D[d] (29), D222E[d] (2), **A73T[e] (1)**	S164T[a] (13), 191L[b] (15), S74R[d] (13)	S164T[a] (5), 191L[b] (5), S74R[d] (5)	-	-	I185T[b] (5), A187D[b] (5), E189Q[b] (5)
	No data (N=3)	Same as above	Same as above	Same as above	Same as above	Same as above	Same as above
	HA not recovered (N=9)	-	-	-	-	-	-
**Antigenic drift by age group**							
Age [years]	1 month-<2 (N=34)	156N[a] (23), K163I[a] (2), K163Q[a] (5), S185T[b] (18), S190V[b] (10), S203T[c] (23), R205K[c] (2), H138R[d] (10), 222D[d] (21), D222E[d] (2)	S164T[a] (9), 191L[b] (11), S74R[d] (9)	S164T[a] (3), 191L[b] (3), S74R[d] (3)	-	-	I185T[b] (3), A187D[b] (3), E189Q[b] (3)
	2-<5 (N=39)	**N125D[a] (1),** 156N[a] (27), K163I[a] (3), K163Q[a] (5), S185T[b] (23), **A186T[b]**	S164T[a] (11), 191L[b] (12), S74R[d] (11)	S164T[a] (7), 191L[b] (7), S74R[d] (7)	-	-	I185T[b] (7), A187D[b] (7), E189Q[b] (7)

		**(1),** S190V^{b} (8), S203T^{c} (27), R205K^{c} (2), **H138Q^{d} (1),** H138R^{d} (9), 222D^{d} (26), D222E^{d} (1), **A73T^{e} (1)**					
	5-<15 (N=20)	156N^{a} (14), K163Q^{a} (2), S185T^{b} (10), S190V^{b} (4), S203T^{c} (14), R205K^{c} (2), H138R^{d} (4), 222D^{d} (14)	S164T^{a} (6), 191L^{b} (6), **K142R^{d} (1),** S74R ^{d} (6)	S164T^{a} (1), 191L^{b} (1), S74R ^{d} (1)	-	-	I185T^{b} (1), A187D^{b} (1), E189Q^{b} (1)
	15-<50 (N=10)	156N^{a} (10), S185T^{b} (2), S203T^{c} (10), R205K^{c} (3), 222D^{d} (10)	-	-	-	-	-
	50-<65 (N=1)	156N^{a} (1), S185T^{b} (1), S190V^{b} (1), S203T^{c} (1), H138R^{d} (1), 222D^{d} (1)	-	-	-	-	-
	≥ 65 (N=0)	-	-	-	-	-	-
	No data (N=3)	Same as above	Same as above	Same as above	Same as above	Same as above	Same as above
	HA not recovered (N=9)	-	-	-	-	-	-
**Antigenic drift by geographical region**							
Region	Central (N=81)	156N^{a} (54), K163I ^{a} (1), K163Q^{a} (11), S185T^{b} (35), S190V^{b} (10), S203T^{c} (54), R205K^{c} (7), **H138Q^{d} (1),** H138R^{d} (10), 222D^{d} (54)	**S164T^{a} (27),** 191L^{b} (27), **K142R^{d} (1), S74R ^{d} (27)**	S164T^{a} (11), 191L^{b} (11), S74R ^{d} (11)	-	-	I185T^{b} (11), A187D^{b} (11), E189Q^{b} (11)
	Eastern (N=3)	**N125D^{a} (1),** 156N^{a} (3**),** K163Q^{a} (1), S185T^{b} (3), S190V^{b} (2), S203T^{c} (3), H138R^{d} (3), 222D^{d} (3)	-	-	-	-	-
	Northwest (N=11)	156N^{a} (11**),** K163I ^{a} (2), S185T^{b} (7), S190V^{b} (3), S203T^{c} (11), H138R^{d} (4), 222D^{d} (7), D**222E^{d} (4)**	-	-	-	-	-
	Western (n=12)	156N^{a} (9**),** K163I ^{a} (2), S185T^{b} (9), S190V^{b} (8), S203T^{c} (9), R205K^{c} (2), H138R^{d} (8), 222D^{d} (9), **A73T^{e} (1)**	191L^{b} (3)	-	-	-	-
	HA not recovered (N=9)	-	-	-	-	-	-

SH= Southern hemisphere vaccine. NH= Northern hemisphere vaccine. SNH= vaccine shared for both Southern and Northern hemispheres. A(H1N1)pdm09 HA1 antigenic sites are Sa= a, Sb =b, Ca_1=c, Ca_2 = d, Cb= e as described by Caton et al., 1982. The frequency of an amino acid substitution observed at a given antigenic site in Uganda HA1 proteins is reported as (n). The substitutions in **BOLD** are unique between subgroups relative to the vaccine of a given season. Amino acid substitutions against the NH vaccine strains are coloured brown for visualization purposes only. Recommended vaccines for each influenza season are adopted from the World Health Organization (WHO, https://www.who.int/teams/global-influenza-programme/vaccines/who-recommendations).

Supplementary Table 3: Antigenic drift among successfully sequenced Uganda A(H1N1)pdm09 viruses (N=107) from 2010-2020 season vaccine viruses, recommended for the Southern (SH), Northern (NH) hemisphere or both (SNH), by cases, gender, age group, and geographical region. Hemagglutinin (HA) sequences were not recovered for 9 H1N1pdm09 viruses.

Supplementary Table 4: Antigenic drift observed among Uganda A(H3N2) viruses per case, gender, age group, and geographical region per year

Season		2010-2011 (n=25)	2012 (n=16)		2013 (n=6)		2014 (n=11)	2015 (n=14)
**Vaccine**		A/Perth/16/2009: E3, A/Perth/16 clade (SNH)	A/Perth/16/2009: E3, A/Perth/16 clade (SH)	A/Victoria/361/2011: E3/E3, Clade 3C.1 (NH)	A/Victoria/361/2011: E3/E3, Clade 3C.1 (SH)	A/Texas/50/2012: E5, Clade 3C.1 (NH)	A/Texas/50/2012: E5, Clade 3C.1 (SNH)	A/Switzerland/9715293/2013: E4/E2, clade 3C.3a (SNH)
**Antigenic drift by case**								
Case	ILI (N= 65)	**A138S[a] (1), I140K[a] (1), R142G[a] (1),** K144N[a] (20), N145S[a] (12), **T128A[b] (1), F159S[b] (1), I192V[b] (3), A196T[b] (1),** A198S[b] (11), **A198P[b] (1), S45N[c] (6), T48I[c] (1), G275S[c] (1), N278K[c] (1),** N312S[c] (11), T212A[d] (20), S214I[d] (20), **N225D[d] (1), I242V[d] (1),** K62E[e] (20), **K83E[e] (1)**	**S124N[a] (1),** R142G[a] (4), K144N[a] (13), N145S[a] (7), T128A[b] (4), A198S[b] (7), S45N[c] (10), T48I[c] (4), N278K[c] (4), N312S[c] (7), **N121K[d] (1),** T212A[d] (13), **S214V[d] (3),** S214I[d] (10), **I242V[d] (1),** K62E[e] (13), **S262N[e] (3)**	**S124N[a] (1),** R142G[a] (4), N145S[a] (7), T128A[b] (4), Q156H[b] (13), V186G[b] (13), S198A[b] (6), **N45S[c] (3),** I48T[c] (9), N278K[c] (4), S312N[c] (6), **N121K[d] (1), I214V[d] (3),** Y219S[d] (13), **I242V[d] (1), S262N[e] (3)**	**N122S[a] (1), I140K[a] (1),** R142G[a] (4), N145S[a] (4), T128A[b] (4), Q156H[b] (4), V186G[b] (4), **Q197H[b] (1),** N278K[c] (4), Y219S[d] (4), **S91G[e] (1)**	**N122S[a] (1), I140K[a] (1),** R142G[a] (4), N145S[a] (4), N128A[b] (4), V186G[b] (4), **Q197H[b] (1),** P198S[b] (4), F219S[d] (4), **S91G[e] (1)**	I140K[a] (4), R142G[a] (4), N145S[a] (4), N128A[b] (4), V186G[b] (4), P198S[b] (3), F219S[d] (4), **N121D[d] (1), E62K[e] (1)**	S138A[a] (4), R140K[a] (1), R140I[a] (1), **G142K[a] (2),** N144S[a] (2), A128T[b] (2), S159F[b] (2), S159Y[b] (2), K160T[b] (2), V186G[b] (4), **Q197R[b] (2),** D53N[c] (2), Q311H[c] (2), 219S[d] (4), D225N[d] (2)
	SARI (N=33)	K144N[a] (4), N145S[a] (4), A198S[b] (4), N312S[c] (4), T212A[d] (4), S214I[d] (4), K62E[e] (4)	**I140K[a] (1),** R142G[a] (1), K144N[a] (3), N145S[a] (1), T128A[b] (1), A198S[b] (1), S45N[c] (3), T48I[c] (1), N278K[c] (1), N312S[c] (1), T212A[d] (3), S214I[d] (3), K62E[e] (3)	**I140K[a] (1),** R142G[a] (1), N145S[a] (1), T128A[b] (1), Q156H[b] (3), V186G[b] (3), S198A[b] (2), I48T[c] (2), N278K[c] (1), S312N[c] (2), Y219S[d] (3)	**S124N[a] (1),** R142G[a] (2), N145S[a] (2), T128A[b] (2), Q156H[b] (2), **L157S[b] (1),** V186G[b] (2), **D53N[c] (1),** N278K[c] (2), **V297I[c] (1), I214T[d] (1),** Y219S[d] (2)	**S124N[a] (1),** R142G[a] (2), N145S[a] (2), N128A[b] (2), **L157S[b] (1),** V186G[b] (2), P198S[b] (2), **D53N[c] (1), V297I[c] (1), I214T[d] (1),** F219S[d] (2)	**A138S[a] (1),** I140K[a] (2), R142G[a] (7), N145S[a] (7), N128A[b] (7), **F159S[b] (1),** V186G[b] (7), P198S[b] (7), F219S[d] (7), **N225D[d] (1)**	**S124N[a] (1), S124R[a] (1),** A128T[b] (4), S138A[a] (9), R140K[a] (4), R140I[a] (6), **G142R[a] (4),** N144S[a] (4), **N144K[a] (1), M168V[a] (2), L157S[b] (1),** S159F[b] (5), S159Y[b] (4), K160T[b] (4), V186G[b] (10), D53N[c] (1), Q311H[c] (4), **N171H[d] (1), I214T[d] (1),** 219S[d] (10), D225N[d] (5), **V88I[e] (1), S91N[e] (1), S262N[e] (1), S265G[e] (1)**
	No data (N=1)	K144N[a] (1), **S44N[c] (1), D53N[c] (1), G275S[c] (1),** T212A[d] (1), S214I[d] (1), K62E[e] (1)	-	-	-	-	-	-
	HA not recovered (N= 19)	-	-	-	-	-	-	-
**Antigenic drift by gender**								
Gender	Male (N=57)	**A138S[a] (1), I140K[a] (1), R142G[a] (1),** K144N[a] (16), N145S[a] (11), **T128A[b] (1), F159S[b] (1),** I192V[b] (2), **A196T[b] (1),** A198S[b] (10), **A198P[b] (1),**	**S124N[a] (1), I140K[a] (1),** R142G[a] (4), K144N[a] (11), N145S[a] (6), T128A[b] (4), A198S[b] (6), S45N[c] (8), T48I[c] (4), N278K[c] (4), N312S[c] (6), **N121K[d]**	**S124N[a] (1), I140K[a] (1),** R142G[a] (4), N145S[a] (6), T128A[b] (4), Q156H[b] (10), V186G[b] (10), S198A[b] (4), N45S[c] (2), I48T[c] (6), N278K[c] (4), S312N[c] (4),	**S124N[a] (1),** R142G[a] (3), N145S[a] (3), T128A[b] (3), Q156H[b] (3), **L157S[b] (1),** V186G[b] (3), **D53N[c] (1),** N278K[c] (3), **V297I[c] (1), I214T[d] (1),** Y219S[d] (3)	**S124N[a] (1),** R142G[a] (3), N145S[a] (3), N128A[b] (3), **L157S[b] (1),** V186G[b] (3), P198S[b] (3), D53N[c] (1), **V297I[c] (1), I214T[d] (1),** F219S[d] (3)	**A138S[a] (1),** I140K[a] (2), R142G[a] (4), N145S[a] (4), N128A[b] (4), V186G[b] (4), P198S[b] (4), **F159S[b] (1),** F219S[d] (4), **N121D[d] (1), N225D[d] (1), E62K[e] (1)**	**S124N[a] (1), S124R[a] (1),** S138A[a] (8), R140K[a] (3), R140I[a] (6), G142K[a] (1), G142R[a] (3), N144S[a] (4), **N144K[a] (1),** M168V[a] (1), A128T[b] (4), **L157S[b] (1),**

		S45N[c] (3), **T48I[c] (1), G275S[c] (1), N278K[c] (1),** N312S[c] (10), T212A[d] (16), S214I[d] (16), **N225D[d] (1), I242V[d] (1),** K62E[e] (16)	**(1),** T212A[d] (11), S214V[d] (2), S214I[d] (8), **I242V[d] (1),** K62E[e] (11), S262N[e] (2)	**N121K[d] (1),** I214V[d] (2), Y219S[d] (10), **I242V[d] (1),** S262N[e] (2)				S159F[b] (4), S159Y[b] (4), K160T[b] (4), V186G[b] (9), Q197R[b] (1), D53N[c] (2), Q311H[c] (4), **I214T[d] (1),** 219S[d] (9), D225N[d] (4), **S91N[e] (1), S265G[e] (1)**
	Female (N=41)	K144N[a] (8), N145S[a] (5), I192V[b] (1), A198S[b] (5), S45N[c] (3), N312S[c] (5), T212A[d] (8), S214I[d] (8), K62E[e] (8), **K83E[e] (1)**	R142G[a] (1), K144N[a] (6), N145S[a] (2), T128A[b] (1), A198S[b] (2), S45N[c] (5), T48I[c] (1), N278K[c] (1), N312S[c] (2), T212A[d] (6), S214I[d] (5), S214V[d] (1), K62E[e] (6), S262N[e] (1)	R142G[a] (1), N145S[a] (2), T128A[b] (1), Q156H[b] (6), V186G[b] (6), S198A[b] (4), N45S[c] (1), I48T[c] (5), N278K[c] (1), S312N[c] (4), I214V[d] (1), Y219S[d] (6), S262N[e] (1)	**N122S[a] (1), I140K[a] (1),** R142G[a] (3), N145S[a] (3), T128A[b] (3), Q156H[b] (3), V186G[b] (3), **Q197H[b] (1),** N278K[c] (3), Y219S[d] (3), **S91G[e] (1)**	**N122S[a] (1), I140K[a] (1),** R142G[a] (3), N145S[a] (3), N128A[b] (3), V186G[b] (3), **Q197H[b] (1),** P198S[b] (3), F219S[d] (3), **S91G[e] (1)**	I140K[a] (4), R142G[a] (7), N145S[a] (7), N128A[b] (7), V186G[b] (7), P198S[b] (6), F219S[d] (7)	S138A[a] (5), R140K[a] (3), R140I[a] (2), G142R[a] (1), G142K[a] (1), N144S[a] (2), M168V[a] (1), A128T[b] (2), S159F[b] (3), S159Y[b] (2), K160T[b] (2), V186G[b] (5), Q197R[b] (1), D53N[c] (1), Q311H[c] (2), **N171H[d] (1),** 219S[d] (5), D225N[d] (3), **S262N[e] (1), V88I[e] (1)**
	No data (N=1)	Same as above	-	-	-	-	-	-
	HA not recovered (N= 19)	-	-	-	-	-	-	-
**Antigenic drift by age group**								
Age [years]	1 month-<2 (N=29)	K144N[a] (4), N145S[a] (2), A198S[b] (2), N312S[c] (2), T212A[d] (4), S214I[d] (4), K62E[e] (4)	**I140K[a] (1),** R142G[a] (2), K144N[a] (7), N145S[a] (4), T128A[b] (2), A198S[b] (4), S45N[c] (5), T48I[c] (2), N278K[c] (2), N312S[c] (4), T212A[d] (7), S214V[d] (2), S214I[d] (5), K62E[e] (7), S262N[e] (2)	**I140K[a] (1),** R142G[a] (2), N145S[a] (4), T128A[b] (2), Q156H[b] (7), V186G[b] (7), S198A[b] (3), N45S[c] (2), I48T[c] (5), N278K[c] (2), S312N[c] (3), I214V[d] (2), Y219S[d] (7), S262N[e] (2)	**S124N[a] (1),** R142G[a] (4), N145S[a] (4), T128A[b] (4), Q156H[b] (4), **L157S[b] (1),** V186G[b] (4), **Q197H[b] (1), D53N[c] (1),** N278K[c] (4), **V297I[c] (1), I214T[d] (1),** Y219S[d] (4), **S91G[e] (1)**	**S124N[a] (1),** R142G[a] (4), N145S[a] (4), N128A[b] (4), **L157S[b] (1),** V186G[b] (4), **Q197H[b] (1),** P198S[b] (4), **D53N[c] (1), V297I[c] (1), I214T[d] (1),** F219S[d] (4), **S91G[e] (1)**	**A138S[a] (1),** I140K[a] (1), R142G[a] (5), N145S[a] (5), N128A[b] (5), **F159S[b] (1),** V186G[b] (5), P198S[b] (5), F219S[d] (5), **N225D[d] (1)**	**S124R[a] (1),** S138A[a] (2), R140I[a] (2), G142R[a] (2), N144S[a] (2), M168V[a] (1), A128T[b] (2), S159Y[b] (2), K160T[b] (2), V186G[b] (2), Q311H[c] (2), 219S[d] (2), **S91N[e] (1), S265G[e] (1)**
	2-<5 (N=41)	**A138S[a] (1), I140K[a] (1), R142G[a] (1),** K144N[a] (9), N145S[a] (7), **T128A[b] (1), F159S[b] (1),** I192V[b] (2), **A196T[b] (1),** A198S[b] (6), **A198P[b] (1), S45N[c] (3), T48I[c] (1), N278K[c] (1),** N312S[c] (6), T212A[d] (9), S214I[d] (9), **N225D[d] (1), I242V[d] (1),** K62E[e] (9), **K83E[e] (1)**	**S124N[a] (1),** R142G[a] (3), K144N[a] (7), N145S[a] (4), T128A[b] (3), A198S[b] (4), S45N[c] (6), T48I[c] (3), N278K[c] (3), N312S[c] (4), T212A[d] (7), S214V[d] (1), S214I[d] (6), **I242V[d] (1),** K62E[e] (7), S262N[e] (1)	**S124N[a] (1),** R142G[a] (3), N145S[a] (4), T128A[b] (3), Q156H[b] (7), V186G[b] (7), S198A[b] (3), N45S[c] (1), I48T[c] (4), N278K[c] (3), S312N[c] (3), I214V[d] (1), Y219S[d] (7), **I242V[d] (1),** S262N[e] (1)	**N122S[a] (1), I140K[a] (1),** R142G[a] (2), N145S[a] (2), T128A[b] (2), Q156H[b] (2), V186G[b] (2), N278K[c] (2), Y219S[d] (2)	**N122S[a] (1), I140K[a] (1),** R142G[a] (2), N145S[a] (2), N128A[b] (2), V186G[b] (2), P198S[b] (2), F219S[d] (2)	I140K[a] (4), R142G[a] (5), N145S[a] (5), N128A[b] (5), V186G[b] (5), P198S[b] (4), F219S[d] (5), **N121D[d] (1), E62K[e] (1)**	**S124N[a] (1),** S138A[a] (7), R140K[a] (5), R140I[a] (3), G142K[a] (1), N144S[a] (1), **N144K[a] (1),** A128T[b] (1), **L157S[b] (1),** S159F[b] (6), S159Y[b] (1), K160T[b] (1), V186G[b] (8), **Q197R[b] (1),** D53N[c] (2), Q311H[c] (1), **I214T[d] (1), N171H[d] (1),** 219S[d] (8), D225N[d] (6), **S262N[e] (1), V88I[e] (1)**
	5-<15 (N=18)	K144N[a] (5), N145S[a] (4), I192V[b] (1), A198S[b] (4), G275S[c] (1), N312S[c] (4), T212A[d] (5), S214I[d] (5), K62E[e] (5)	K144N[a] (2), S45N[c] (2), N121K[d] (1), T212A[d] (2), S214I[d] (2), K62E[e] (2)	Q156H[b] (2), V186G[b] (2), S198A[b] (2), I48T[c] (2), S312N[c] (2), N121K[d] (1), Y219S[d] (2)	**-**	**-**	I140K[a] (1), R142G[a] (1), N145S[a] (1), N128A[b] (1), V186G[b] (1), P198S[b] (1), F219S[d] (1)	S138A[a] (2), R140K[a] (1), R140I[a] (1), G142K[a] (1), N144S[a] (1), A128T[b] (1), S159F[b] (1), S159Y[b] (1), K160T[b] (1), V186G[b] (2), Q197R[b] (1), D53N[c] (1),

								Q311H[c] (1), 219S[d] (2), D225N[d] (1)
	15-<50 (N=9)	K144N[a] (5), N145S[a] (2), A198S[b] (2), S45N[c] (3), N312S[c] (2), T212A[d] (5), S214I[d] (5), K62E[e] (5)	-	-	-	-	-	S138A[a] (2), R140I[a] (2), G142R[a] (2), N144S[a] (2), M168V[a] (1), A128T[b] (2), S159Y[b] (2), K160T[b] (2), V186G[b] (2), Q311H[c] (2), 219S[d] (2)
	50-<65 (N=1)	K144N[a] (1), N145S[a] (1), A198S[b] (1), N312S[c] (1), T212A[d] (1), S214I[d] (1), K62E[e] (1)	-	-	-	-	-	-
	≥ 65 (N=0)	-	-	-	-	-	-	-
	No data (N=1)	Same as above	-	-	-	-	-	-
	HA not recovered (N= 19)	-	-	-	-	-	-	-
**Antigenic drift by age region**								
Region	Central (N=80)	A138S[a] (1), I140K[a] (1), R142G[a] (1), K144N[a] (25), N145S[a] (16), T128A[b] (1), F159S[b] (1), I192V[b] (3), A196T[b] (1), A198S[b] (15), A198P[b] (1), S45N[c] (7), T48I[c] (1), D53N[c] (1), G275S[c] (2), N278K[c] (1), N312S[c] (15), T212A[d] (25), S214I[d] (25), N225D[d] (1), I242V[d] (1), K62E[e] (25), K83E[e] (1)	**S124N[a] (1),** R142G[a] (4), K144N[a] (13), N145S[a] (7), T128A[b] (4), A198S[b] (7), S45N[c] (10), T48I[c] (4), N278K[c] (4), N312S[c] (7), T212A[d] (13), **S214V[d] (3),** S214I[d] (10), **I242V[d] (1),** K62E[e] (13), **S262N[e] (3)**	**S124N[a] (1),** R142G[a] (4), N145S[a] (7), T128A[b] (4), Q156H[b] (13), V186G[b] (13), S198A[b] (6), **N45S[c] (3),** I48T[c] (9), N278K[c] (4), S312N[c] (6), **I214V[d] (3),** Y219S[d] (13), **I242V[d] (1), S262N[e] (3)**	**N122S[a] (1), I140K[a] (1),** R142G[a] (5), N145S[a] (5), T128A[b] (5), Q156H[b] (5), V186G[b] (5), **Q197H[b] (1),** N278K[c] (5), Y219S[d] (5), **S91G[e] (1)**	N122S[a] (1), I140K[a] (1), R142G[a] (5), N145S[a] (5), N128A[b] (5), V186G[b] (5), **Q197H[b] (1),** P198S[b] (5), F219S[d] (5), **S91G[e] (1)**	**I140K[a] (6),** R142G[a] (7), N145S[a] (7), N128A[b] (7), V186G[b] (7), P198S[b] (6), F219S[d] (7), **N121D[d] (1), E62K[e] (1)**	S138A[a] (6), R140K[a] (3), R140I[a] (3), G142R[a] (1), G142K[a] (2), N144S[a] (3), M168V[a] (1), A128T[b] (3), S159F[b] (3), S159Y[b] (3), K160T[b] (3), V186G[b] (6), **Q197R[b] (2),** D53N[c] (2), Q311H[c] (3), 219S[d] (6), D225N[d] (3)
	Eastern (N=7)	-	-	-	**S124N[a] (1),** R142G[a] (1), N145S[a] (1), T128A[b] (1), Q156H[b] (1), **L157S[b] (1),** V186G[b] (1), **D53N[c] (1),** N278K[c] (1), **V297I[c] (1), I214T[d] (1),** Y219S[d] (1)	**S124N[a] (1),** R142G[a] (1), N145S[a] (1), N128A[b] (1), **L157S[b] (1),** V186G[b] (1), P198S[b] (1), **D53N[c] (1), V297I[c] (1), I214T[d] (1),** F219S[d] (1)	-	**S124N[a] (1),** S138A[a] (4), R140K[a] (2), R140I[a] (2), G142R[a] (1), N144S[a] (1), A128T[b] (1), L157S[b] (1), S159F[b] (3), S159Y[b] (1), K160T[b] (1), V186G[b] (4), D53N[c] (1), Q311H[c] (1), I214T[d] (1), N171H[d] (1), 219S[d] (4), D225N[d] (3)
	Northwest (N=3)	-	K144N[a] (2), S45N[c] (2), N121K[d] (1), T212A[d] (2), S214I[d] (2), K62E[e] (2)	Q156H[b] (2), V186G[b] (2), S198A[b] (2), I48T[c] (2), S312N[c] (2), **N121K[d] (1),** Y219S[d] (2)	-	-	**A138S[a] (1),** R142G[a] (1), N145S[a] (1), N128A[b] (1), **F159S[b] (1),** V186G[b] (1), P198S[b] (1), F219S[d] (1), **N225D[d] (1)**	-

	Western (N=9)	-	**I140K^{a} (1),** R142G^{a} (1), K144N^{a} (1), N145S^{a} (1), T128A^{b} (1), A198S^{b} (1), S45N^{c} (1), T48I^{c} (1), N278K^{c} (1), N312S^{c} (1), T212A^{d} (1), S214I^{d} (1), K62E^{e} (1)	I140K^{a} (1), R142G^{a} (1), N145S^{a} (1), T128A^{b} (1), Q156H^{b} (1), V186G^{b} (1), N278K^{c} (1), Y219S^{d} (1)	-	-	R142G^{a} (3), N145S^{a} (3), N128A^{b} (3), V186G^{b} (3), P198S^{b} (3), F219S^{d} (3)	**S124R^{a} (1),** S138A^{a} (3), R140K^{a} (1), R140I^{a} (3), G142R^{a} (1), N144S^{a} (2), **N144K^{a} (1),** M168V^{a} (1), A128T^{b} (2), S159F^{b} (1), S159Y^{b} (2), K160T^{b} (2), V186G^{b} (4), Q311H^{c} (2), 219S^{d} (4), D225N^{d} (1), V88I^{e} (1), S91N^{e} (1), **S262N^{e} (1), S265G^{e} (1)**
	HA not recovered (N= 19)	-	-	-	-	-	-	-

SH= Southern hemisphere vaccine. NH= Northern hemisphere vaccine. SNH= vaccine shared for both Southern and Northern hemispheres. A(H3N2) HA1 antigenic sites are A=a, B=b, C=c, D=d, and E=e as described by Wiley and Skehel, 1987. The frequency of an amino acid substitution observed at a given antigenic site in Uganda HA1 proteins is reported as (n). The substitutions in **BOLD** are unique between subgroups relative to the vaccine of a given season. Amino acid substitutions against the NH vaccine strains are coloured brown for visualization purposes only. Recommended vaccines for each influenza season are adopted from the World Health Organization (WHO, https://www.who.int/teams/global-influenza-programme/vaccines/who-recommendations).

Supplementary Table 4: Antigenic drift among successfully sequenced Uganda A(H3N2) viruses (N=99) from 2010-2020 season vaccine viruses, recommended for the Southern (SH), Northern (NH) hemisphere or both (SNH), by cases, gender, age, and geographical region. Hemagglutinin (HA) sequences were not recovered for 19 H1N1pdm09 viruses.

Supplementary Table 4 (continued): Antigenic drift observed among Uganda A(H3N2) viruses per case, gender, age group, and geographical region per year

Season		2016-2017 (n=27)	2018 (Only 2017 viruses analysed, n=13)	2019 (Only 2017 viruses analysed, n=13)		2020 (Only 2017 viruses analysed, n=13)	
**Vaccine**		**A/Hong Kong/4801/2014:** E5/E3, clade 3C.2a (SNH)	**A/Singapore/INFIMH-16-0019/2016**: E5, Clade 3C.2a1 (SNH)	**A/Switzerland/8060/2017**: E5/E2, Clade 3C.2a2 (SH)	**A/Kansas/14/2017**: E5, Clade 3C.3a (NH)	**A/South Australia/34/2019**: E5, Clade 3C.2a1b (SH)	**A/Hong Kong/2671/2019**: E7, Clade 3C.2a1b (NH)
**Antigenic drift by case**							
Case	ILI (N= 65)	**N122D^{a} (3), A138S^{a} (6), I140K^{a} (9),** R142G^{a} (9), R142K^{a} (3), S144N^{a} (11), S144K^{a} (1), **S145N^{a} (2), S146G^{a} (1),** T128A^{b} (9), **T135K^{b} (2), H156Q^{b} (1),** Y159F^{b} (5), **Y159S^{b} (6),** K160T^{b} (8), P194L^{b} (20), **A196T^{b} (2),** Q197R^{b} (3), **S198A^{b} (2),** S198P^{b} (6), **I48T^{c} (2), D53E^{c} (2),** D53N^{c} (3), **G275S^{c} (1), K276R^{c} (1), K278N^{c} (2),** H311Q^{c} (14), **S312N^{c} (8),** S96N^{d} (19), N121K^{d} (6), N171K^{d} (5), 203T^{d}	**N122D^{a} (2), A138S^{a} (5),** I140K^{a} (5), **G142R^{a} (6), S144N^{a} (7), S145N^{a} (2), S146G^{a} (1), T128A^{b} (5), T135K^{b} (2), H156Q^{b} (1), Y159F^{b} (2), Y159S^{b} (5),** K160T^{b} (4), P194L^{b} (11), **A196T^{b} (2), S198A^{b} (2), S198P^{b} (5), I48T^{c} (2), D53E^{c} (1), G275S^{c} (1), K276R^{c} (1), K278N^{c} (2),** H311Q^{c} (9), **S312N^{c} (7),** K121N^{d} (7), K171N^{d} (7), I214T^{d} (1), **225N^{d} (2),** 225D^{d} (9), **Q75H^{e} (1),**	**N122D^{a} (2),** K131T^{a} (11), **A138S^{a} (5), I140K^{a} (5),** K142R^{a} (6), **K142G^{a} (5), S144N^{a} (7), S145N^{a} (2), S146G^{a} (1), T128A^{b} (5), T135K^{b} (2), H156Q^{b} (1), Y159F^{b} (2), Y159S^{b} (5),** K160T^{b} (4), P194L^{b} (11), **A196T^{b} (2), S198A^{b} (2), S198P^{b} (5), I48T^{c} (2), D53E^{c} (1), G275S^{c} (1), K276R^{c} (1), K278N^{c} (2),** 300I^{c} (11), H311Q^{c} (9), **S312N^{c} (7),** S96N^{d} (11), N121K^{d} (4), N171K^{d} (4), I214T^{d} (1), **D225N^{d} (2), Q75H^{e}**	**N122D^{a} (2),** S138A^{a} (6), **I140K^{a} (5),** G142R^{a} (6), K144S^{a} (4), **K144N^{a} (7), S145N^{a} (2), S146G^{a} (1),** A128T^{b} (6), **T135K^{b} (2), H156Q^{b} (1), S159F^{b} (2),** S159Y^{b} (4), K160T^{b} (4), N190D^{b} (4), S193F^{b} (11), A196T^{b} (2), **S198A^{b} (2), S198P^{b} (5), I48T^{c} (2), D53E^{c} (1), G275S^{c} (1), K276R^{c} (1), K278N^{c} (2),** Q311H^{c} (2), **S312N^{c} (7),** N121K^{d} (4), N171K^{d} (4), I214T^{d} (1), **D225N^{d} (2),** 246N^{d} (11), **Q75H^{e} (1),**	**N122D^{a} (2),** K131T^{a} (11), **A138S^{a} (5), I140K^{a} (5),** G142R^{a} (6), **S144N^{a} (7), S145N^{a} (2), S146G^{a} (1), T128A^{b} (5), T135K^{b} (2), H156Q^{b} (1), Y159F^{b} (2), Y159S^{b} (5),** K160T^{b} (4), I186G^{b} (11), A196T^{b} (2), **S198A^{b} (2), S198P^{b} (5), I48T^{c} (2), D53E^{c} (1), G275S^{c} (1), K276R^{c} (1), K278N^{c} (2),** Q311H^{c} (2), **S312N^{c} (7),** K121N^{d} (7), K171N^{d} (7), I214T^{d} (1), F219S^{d} (11), **D225N^{d} (2),**	**N122D^{a} (2),** F137S^{a} (11), S138A^{a} (6), **I140K^{a} (5),** G142R^{a} (6), **S144N^{a} (7), S145N^{a} (2), S146G^{a} (1),** A128T^{b} (6), K135T^{b} (9), **H156Q^{b} (1), Y159F^{b} (2), Y159S^{b} (5),** I160T^{b} (4), **I160K^{b} (7),** V186G^{b} (11), S193F^{b} (11), **A196T^{b} (2), S198A^{b} (2), S198P^{b} (5), I48T^{c} (2), D53E^{c} (1), G275S^{c} (1), K276R^{c} (1), K278N^{c} (2),** Q311H^{c} (2), **S312N^{c} (7),** K121N^{d} (7), K171N^{d} (7), I214T^{d} (1),

		(20), **K207Q[d] (1),** I214T[d] (1), D225N[d] (5), **E62K[e] (1), Q75H[e] (2), G78D[e] (2), K83E[e] (1), S91N[e] (1),** K92R[e] (3), **Y94H[e] (2), S262N[e] (1)**	**G78D[e] (2), K83E[e] (1),** K92R[e] (2), **Y94H[e] (2)**	**(1), G78D[e] (2), K83E[e] (1),** K92R[e] (2), **Y94H[e] (2),** Q261R[e] (11)	**G78D[e] (2), K83E[e] (1),** N91S[e] (11), K92R[e] (2), **Y94H[e] (2)**	G62E[e] (11), **Q75H[e] (1), G78D[e] (2), K83E[e] (1),** R92K[e] (9), **Y94H[e] (2)**	N225D[d] (9), G62E[e] (11), **Q75H[e] (1), G78D[e] (2), K83E[e] (1),** R92K[e] (9), **Y94H[e] (2)**
	SARI (N=33)	**S124N[a] (1),** R142G[a] (3), R142K[a] (2), S144N[a] (2), S144K[a] (1), T128A[b] (2), **L157S[b] (1),** Y159F[b] (2), K160T[b] (5), P194L[b] (7), Q197R[b] (2), S198P[b] (1), D53N[c] (4), H311Q[c] (4), S96N[d] (7), N121K[d] (3), N171K[d] (2), 203T[d] (7), I214T[d] (2), D225N[d] (2), **E62G[e] (1),** K92R[e] (2)	**G140R[a] (1),** G140K[a] (1), K160T[b] (1), P194L[b] (2), **Q197R[b] (1), D53N[c] (2),** H311Q[c] (1), K121N[d] (1), K171N[d] (1), I214T[d] (1), 225D[d] (2), K92R[e] (1)	K131T[a] (2), K142R[a] (2), K160T[b] (2), P194L[b] (2), **Q197R[b] (1), D53N[c] (2),** 300I[c] (2), H311Q[c] (1), S96N[d] (2), N121K[d] (1), N171K[d] (1), I214T[d] (1), K92R[e] (1), Q261R[e] (2)	S138A[a] (2), G142R[a] (1), **G142K[a] (1),** K144S[a] (2), A128T[b] (2), S159Y[b] (2), K160T[b] (2), N190D[b] (2), S193F[b] (2), **Q197R[b] (1), D53N[c] (2),** Q311H[c] (1), N121K[d] (1), N171K[d] (1), I214T[d] (1), 246N[d] (2), N91S[e] (2), K92R[e] (1)	K131T[a] (2), G142R[a] (1), **G142K[a] (1),** K160T[b] (2), I186G[b] (2), **Q197R[b] (1), D53N[c] (2),** Q311H[c] (1), K121N[d] (1), K171N[d] (1), I214T[d] (1), F219S[d] (2), G62E[e] (2), R92K[e] (1)	F137S[a] (2), S138A[a] (2), G142R[a] (1), **G142K[a] (1),** A128T[b] (2), K135T[b] (2), I160T[b] (2), V186G[b] (2), S193F[b] (2), **Q197R[b] (1), D53N[c] (2),** Q311H[c] (1), K121N[d] (1), K171N[d] (1), I214T[d] (1), N225D[d] (2), G62E[e] (2), R92K[e] (1)
	No data (N=1)	-	-	-	-	-	-
	HA not recovered (N= 19)	-	-	-	-	-	-
**Antigenic drift by gender**							
Gender	Male (N=57)	N122D[a] (2), **S124N[a] (1),** A138S[a] (3), I140K[a] (4), R142G[a] (5), R142K[a] (3), S144N[a] (5), S144K[a] (1), **S146G[a] (1),** T128A[b] (5), **T135K[b] (2), L157S[b] (1), Y159F[b] (2),** Y159S[b] (3), K160T[b] (10), P194L[b] (15), Q197R[b] (3), S198P[b] (4), **D53E[c] (2),** D53N[c] (5), H311Q[c] (9), S312N[c] (3), S96N[d] (15), N121K[d] (7), N171K[d] (6), 203T[d] (15), I214T[d] (3), D225N[d] (2), **Q75H[e] (2), G78D[e] (2),** K92R[e] (4), **Y94H[e] (2)**	**N122D[a] (2),** A138S[a] (3), I140K[a] (3), G142R[a] (5), S144N[a] (3), **S146G[a] (1),** T128A[b] (3), **T135K[b] (2),** Y159S[b] (3), K160T[b] (5), P194L[b] (8), S198P[b] (3), **D53E[c] (1),** D53N[c] (1), H311Q[c] (6), S312N[c] (3), K121N[d] (3), K171N[d] (3), **I214T[d] (2),** 225D[d] (8), **Q75H[e] (1), G78D[e] (2), K92R[e] (3), Y94H[e] (2)**	**N122D[a] (2),** K131T[a] (8), A138S[a] (3), I140K[a] (3), K142R[a] (5), K142G[a] (3), S144N[a] (3), **S146G[a] (1),** T128A[b] (3), **T135K[b] (2),** Y159S[b] (3), K160T[b] (5), P194L[b] (8), S198P[b] (3), **D53E[c] (1),** D53N[c] (1), 300I[c] (8), H311Q[c] (6), S312N[c] (3), S96N[d] (8)**, N121K[d] (5), N171K[d] (5), I214T[d] (2), Q75H[e] (1), G78D[e] (2), K92R[e] (3), Y94H[e] (2),** Q261R[e] (8)	**N122D[a] (2),** S138A[a] (5), I140K[a] (3), G142R[a] (5), K144S[a] (3), K144N[a] (5), **S146G[a] (1),** A128T[b] (5), **T135K[b] (2),** S159Y[b] (5), K160T[b] (5), N190D[b] (8), S193F[b] (8), S198P[b] (3), **D53E[c] (1),** D53N[c] (1), Q311H[c] (2), S312N[c] (3), **N121K[d] (5), N171K[d] (5), I214T[d] (2),** 246N[d] (8), **Q75H[e] (1), G78D[e] (2),** N91S[e] (8), **K92R[e] (3), Y94H[e] (2)**	**N122D[a] (2),** K131T[a] (8), A138S[a] (3), I140K[a] (3), G142R[a] (5), S144N[a] (3), **S146G[a] (1),** T128A[b] (3), **T135K[b] (2),** Y159S[b] (3), K160T[b] (5), I186G[b] (8), S198P[b] (3), **D53E[c] (1),** D53N[c] (1), Q311H[c] (2), S312N[c] (3), K121N[d] (3), K171N[d] (3), **I214T[d] (2),** F219S[d] (8), G62E[e] (8), **Q75H[e] (1), G78D[e] (2),** R92K[e] (5), **Y94H[e] (2)**	**N122D[a] (2),** F137S[a] (8), S138A[a] (5), I140K[a] (3), G142R[a] (5), S144N[a] (3), **S146G[a] (1),** A128T[b] (5), K135T[b] (6), Y159S[b] (3), I160T[b] (5), I160K[b] (3), V186G[b] (8), S193F[b] (8), S198P[b] (3), D53E[c] (1), D53N[c] (1), Q311H[c] (2), S312N[c] (3), K121N[d] (3), K171N[d] (3), **I214T[d] (2),** N225D[d] (8), G62E[e] (8), **Q75H[e] (1), G78D[e] (2),** R92K[e] (5), **Y94H[e] (2)**
	Female (N=41)	N122D[a] (1), A138S[a] (3), I140K[a] (5), R142G[a] (7), R142K[a] (2), S144N[a] (8), S144K[a] (1), S145N[a] (2), T128A[b] (6), **H156Q[b] (1), Y159F[b] (5),** Y159S[b] (3), K160T[b] (4), P194L[b] (12), **A196T[b] (2),** Q197R[b] (2), **S198A[b] (2),** S198P[b] (3), **I48T[c] (2),** D53N[c] (2), **G275S[c] (1), K276R[c] (1), K278N[c] (2),** H311Q[c] (9), S312N[c] (5), S96N[d] (11), N121K[d] (2), N171K[d] (1), 203T[d]	A138S[a] (2), I140K[a] (2), G142R[a] (2), **G142K[a] (1),** S144N[a] (4), **S145N[a] (2),** T128A[b] (2), **H156Q[b] (1), Y159F[b] (2),** Y159S[b] (2), K160T[b] (1), P194L[b] (5), **A196T[b] (2), Q197R[b] (1), S198A[b] (2),** S198P[b] (2), **I48T[c] (2),** D53N[c] (1), **G275S[c] (1), K276R[c] (1), K278N[c] (2),** H311Q[c] (4), S312N[c] (4), K121N[d] (5),	K131T[a] (5), A138S[a] (2), I140K[a] (2), K142R[a] (2), K142G[a] (2), S144N[a] (4), **S145N[a] (2),** T128A[b] (2), **H156Q[b] (1), Y159F[b] (2),** Y159S[b] (2), K160T[b] (1), P194L[b] (5), **A196T[b] (2), Q197R[b] (1), S198A[b] (2),** S198P[b] (2), **I48T[c] (2),** D53N[c] (1), **G275S[c] (1), K276R[c] (1), K278N[c] (2),** 300I[c] (5), H311Q[c] (4), S312N[c] (4), S96N[d] (5), **D225N[d] (2)**, **K83E[e] (1),** Q261R[e] (5)	S138A[a] (3), I140K[a] (2), G142R[a] (2), **G142K[a] (1),** K144S[a] (1), K144N[a] (4), S145N[a] (2), A128T[b] (3), **H156Q[b] (1), S159F[b] (2),** S159Y[b] (1), K160T[b] (1), N190D[b] (5), S193F[b] (5), **A196T[b] (2), Q197R[b] (1), S198A[b] (2),** S198P[b] (2), **I48T[c] (2),** D53N[c] (1) **G275S[c] (1), K276R[c] (1), K278N[c] (2),** Q311H[c] (1), S312N[c] (4),	K131T[a] (5), A138S[a] (2), I140K[a] (2), G142R[a] (2), **G142K[a] (1),** S144N[a] (4), **S145N[a] (2),** T128A[b] (2), **H156Q[b] (1), Y159F[b] (2), Y159S[b] (2),** K160T[b] (1), I186G[b] (5), **A196T[b] (2), Q197R[b] (1), S198A[b] (2),** S198P[b] (2), **I48T[c] (2),** D53N[c] (1), **G275S[c] (1), K276R[c] (1), K278N[c] (2),** Q311H[c] (1), S312N[c] (4), K121N[d] (5), K171N[d] (5),	F137S[a] (5), S138A[a] (3), I140K[a] (2), G142R[a] (2), **G142K[a] (1),** S144N[a] (4), **S145N[a] (2),** A128T[b] (3), K135T[b] (5), **H156Q[b] (1), Y159F[b] (2),** Y159S[b] (2), I160T[b] (1), I160K[b] (4), V186G[b] (5), S193F[b] (5), A196T[b] (2), Q197R[b] (1), **S198A[b] (2),** S198P[b] (2), **I48T[c] (2),** D53N[c] (1), **G275S[c] (1), K276R[c] (1), K278N[c] (2),** Q311H[c] (1),

		(12), **K207Q^{d} (1),** D225N^{d} (5), **E62G^{e} (1), E62K^{e} (1), K83E^{e} (1), S91N^{e} (1),** K92R^{e} (1), **S262N^{e} (1)**	K171N^{d} (5), **225N^{d} (2),** 225D^{d} (3), **K83E^{e} (1)**		**D225N^{d} (2),** 246N^{d} (5), **K83E^{e} (1),** N91S^{e} (5)	F219S^{d} (5), **D225N^{d} (2),** G62E^{e} (5), **K83E^{e} (1),** R92K^{e} (5)	S312N^{c} (4), K121N^{d} (5), K171N^{d} (5), N225D^{d} (3), G62E^{e} (5), **K83E^{e} (1),** R92K^{e} (5)
	No gender data (N=1)	Same as above	-	-	-	-	-
	HA not recovered (N= 11+8)	-	-	-	-	-	-
**Antigenic drift by age group**							
Age [years]	1 month-<2 (N=29)	N122D^{a} (1), **S124N^{a} (1),** T128A^{b} (3), I140K^{a} (2), R142G^{a} (3), S144N^{a} (3), **S144K^{a} (2), S146G^{a} (1), L157S^{b} (1),** Y159F^{b} (3), K160T^{b} (4), P194L^{b} (7), S198P^{b} (1), D53E^{c} (1), D53N^{c} (1), H311Q^{c} (5), S96N^{d} (7), N121K^{d} (4), N171K^{d} (2), 203T^{d} (7), **K207Q^{d} (1),** I214T^{d} (2), D225N^{d} (3), Q75H^{e} (1), K92R^{e} (2), **S262N^{e} (1)**	G142R^{a} (2), S146G^{a} (1), K160T^{b} (2), P194L^{b} (2), **D53E^{c} (1),** H311Q^{c} (2), I214T^{d} (1), 225D^{d} (2), **Q75H^{e} (1),** K92R^{e} (2)	K131T^{a} (2), K142R^{a} (2), S146G^{a} (1), K160T^{b} (2), P194L^{b} (2), **D53E^{c} (1),** 300I^{c} (2), H311Q^{c} (2), S96N^{d} (2), N121K^{d} (2), N171K^{d} (2), I214T^{d} (1), **Q75H^{e} (1),** K92R^{e} (2), Q261R^{e} (2)	S138A^{a} (2), G142R^{a} (2), K144S^{a} (2), S146G^{a} (1), A128T^{b} (2), S159Y^{b} (2), K160T^{b} (2), N190D^{b} (2), S193F^{b} (2), **D53E^{c} (1),** N121K^{d} (2), N171K^{d} (2), I214T^{d} (1), 246N^{d} (2), **Q75H^{e} (1),** N91S^{e} (2), K92R^{e} (2)	K131T^{a} (2), G142R^{a} (2), S146G^{a} (1), K160T^{b} (2), I186G^{b} (2), **D53E^{c} (1),** I214T^{d} (1), F219S^{d} (2), G62E^{e} (2), **Q75H^{e} (1)**	F137S^{a} (2), S138A^{a} (2), G142R^{a} (2), S146G^{a} (1), A128T^{b} (2), K135T^{b} (2), I160T^{b} (2), V186G^{b} (2), S193F^{b} (2), **D53E^{c} (1),** I214T^{d} (1), N225D^{d} (2), G62E^{e} (2), **Q75H^{e} (1)**
	2-<5 (N=41)	N122D^{a} (1), T128A^{b} (5), T135K^{b} (1), A138S^{a} (3), I140K^{a} (4), R142G^{a} (5), R142K^{a} (3), S144N^{a} (5), **H156Q^{b} (1),** Y159F^{b} (2), Y159S^{b} (3), K160T^{b} (5), P194L^{b} (10), A196T^{b} (1), Q197R^{b} (3), S198P^{b} (3), D53N^{c} (4), H311Q^{c} (6), S312N^{c} (3), S96N^{d} (9), N121K^{d} (2), N171K^{d} (2), 203T^{d} (10), I214T^{d} (1), D225N^{d} (2), **E62K^{e} (1),** G78D^{e} (1), K92R^{e} (1), Y94H^{e} (1)	N122D^{a} (1), A138S^{a} (2), I140K^{a} (2), G142R^{a} (2), **G142K^{a} (1),** S144N^{a} (2), T128A^{b} (2), T135K^{b} (1), **H156Q^{b} (1),** Y159S^{b} (2), K160T^{b} (3), P194L^{b} (5), A196T^{b} (1), Q197R^{b} (1), S198P^{b} (2), **D53N^{c} (2),** H311Q^{c} (3), S312N^{c} (2), K121N^{d} (3), K171N^{d} (3), I214T^{d} (1), 225D^{d} (5), G78D^{e} (1), K92R^{e} (1), Y94H^{e} (1)	N122D^{a} (1), K131T^{a} (5), A138S^{a} (2), I140K^{a} (2), K142R^{a} (2), K142G^{a} (2), S144N^{a} (2), T128A^{b} (2), T135K^{b} (1), **H156Q^{b} (1),** Y159S^{b} (2), K160T^{b} (3), P194L^{b} (5), A196T^{b} (1), Q197R^{b} (1), S198P^{b} (2), **D53N^{c} (2),** 300I^{c} (5), H311Q^{c} (3), S312N^{c} (2), S96N^{d} (5), N121K^{d} (2), N171K^{d} (2), I214T^{d} (1), G78D^{e} (1), K92R^{e} (1), Y94H^{e} (1), Q261R^{e} (5)	N122D^{a} (1), S138A^{a} (3), I140K^{a} (2), G142R^{a} (2), **G142K^{a} (1),** K144S^{a} (3), K144N^{a} (2), A128T^{b} (3), T135K^{b} (1), **H156Q^{b} (1),** S159Y^{b} (3), K160T^{b} (3), N190D^{b} (5), S193F^{b} (5), A196T^{b} (1), Q197R^{b} (1), S198P^{b} (2), **D53N^{c} (2),** Q311H^{c} (2), S312N^{c} (2), N121K^{d} (2), N171K^{d} (2), I214T^{d} (1), G78D^{e} (1), N91S^{e} (5), K92R^{e} (1), Y94H^{e} (1)	N122D^{a} (1), K131T^{a} (5), A138S^{a} (2), I140K^{a} (2), G142R^{a} (2), **G142K^{a} (1),** S144N^{a} (2), T128A^{b} (2), T135K^{b} (1), **H156Q^{b} (1),** Y159S^{b} (2), K160T^{b} (3), I186G^{b} (5), A196T^{b} (1), Q197R^{b} (1), S198P^{b} (2), **D53N^{c} (2),** Q311H^{c} (2), S312N^{c} (2), K121N^{d} (3), K171N^{d} (3), I214T^{d} (1), F219S^{d} (5), G62E^{e} (5), G78D^{e} (1), R92K^{e} (4), Y94H^{e} (1)	N122D^{a} (1), F137S^{a} (5), S138A^{a} (3), I140K^{a} (2), G142R^{a} (2), **G142K^{a} (1),** S144N^{a} (2), A128T^{b} (3), K135T^{b} (4), **H156Q^{b} (1),** Y159S^{b} (2), I160T^{b} (3), I160K^{b} (2), V186G^{b} (5), S193F^{b} (5), A196T^{b} (1), Q197R^{b} (1), S198P^{b} (2), **D53N^{c} (2),** Q311H^{c} (2), S312N^{c} (2), K121N^{d} (3), K171N^{d} (3), I214T^{d} (1), N225D^{d} (5), G62E^{e} (5), G78D^{e} (1), R92K^{e} (5), Y94H^{e} (1)

	5-<15 (N=18)	N122D[a] (1), T128A[b] (3), T135K[b] (1), A138S[a] (3), I140K[a] (3), R142G[a] (4), R142K[a] (1), S144N[a] (4), S145N[a] (1), Y159F[b] (1), Y159S[b] (3), K160T[b] (4), P194L[b] (8), A196T[b] (1), Q197R[b] (1), S198A[b] (3), S198P[b] (1), I48T[c] (1), D53E[c] (1), D53N[c] (1), K278N[c] (1), H311Q[c] (6), S312N[c] (4), S96N[d] (8), N121K[d] (3), N171K[d] (3), 203T[d] (8), D225N[d] (1), **E62G[e] (1),** Q75H[e] (1), G78D[e] (1), K83E[e] (1), S91N[e] (1), K92R[e] (2), Y94H[e] (1)	N122D[a] (1), A138S[a] (3), I140K[a] (3), G142R[a] (2), S144N[a] (4), S145N[a] (1), T128A[b] (3), T135K[b] (1), **Y159F[b] (1),** Y159S[b] (3), K160T[b] (1), P194L[b] (5), A196T[b] (1), S198A[b] (1), S198P[b] (3), I48T[c] (1), K278N[c] (1), H311Q[c] (4), S312N[c] (4), K121N[d] (4), K171N[d] (4), 225N[d] (1), 225D[d] (4), G78D[e] (1), **K83E[e] (1),** Y94H[e] (1)	N122D[a] (1), K131T[a] (5), A138S[a] (3), I140K[a] (3), K142R[a] (2), K142G[a] (3), S144N[a] (4), S145N[a] (1), T128A[b] (3), T135K[b] (1), **Y159F[b] (1),** Y159S[b] (3), K160T[b] (1), P194L[b] (5), A196T[b] (1), S198A[b] (1), S198P[b] (3), I48T[c] **(1),** K278N[c] (1), 300I[c] (5), H311Q[c] (4), S312N[c] (4), S96N[d] (5), N121K[d] (1), N171K[d] (1), D225N[d] (1), G78D[e] (1), **K83E[e]** (1), Y94H[e] (1), Q261R[e] (5)	N122D[a] (1), S138A[a] (2), I140K[a] (3), G142R[a] (2), K144S[a] (1), K144N[a] (4), S145N[a] (1), A128T[b] (2), T135K[b] (1), **S159F[b] (1),** S159Y[b] (1), K160T[b] (1), N190D[b] (5), S193F[b] (5), A196T[b] (1), S198A[b] (1), S198P[b] (3), I48T[c] (1), K278N[c] (1), Q311H[c] (1), S312N[c] (4), N121K[d] (1), N171K[d] (1), D225N[d] (1), 246N[d] (5), G78D[e] (1), **K83E[e] (1),** N91S[e] (5), Y94H[e] (1)	N122D[a] (1), K131T[a] (5), A138S[a] (3), I140K[a] (3), G142R[a] (2), S144N[a] (4), S145N[a] (1), T128A[b] (3), T135K[b] (1), **Y159F[b] (1),** Y159S[b] (3), K160T[b] (1), I186G[b] (5), A196T[b] (1), S198A[b] (1), S198P[b] (3), I48T[c] (1), K278N[c] (1), Q311H[c] (1), S312N[c] (4), K121N[d] (4), K171N[d] (4), F219S[d] (5), D225N[d] (1), G62E[e] (5), G78D[e] (1), **K83E[e] (1),** R92K[e] (5), Y94H[e] (1)	N122D[a] (1), F137S[a] (5), S138A[a] (2), I140K[a] (3), G142R[a] (2), S144N[a] (4), S145N[a] (1), A128T[b] (2), K135T[b] (4), **Y159F[b] (1),** Y159S[b] (3), I160T[b] (1), I160K[b] (4), V186G[b] (5), S193F[b] (5), A196T[b] (1), S198A[b] (1), S198P[b] (3), I48T[c] (1), K278N[c] (1), Q311H[c] (1), S312N[c] (4), K121N[d] (4), K171N[d] (4), N225D[d] (4), G62E[e] (5), G78D[e] (1), **K83E[e] (1),** R92K[e] (5), Y94H[e] (1)
	15-<50 (N=9)	R142K[a] (1), S144N[a] (1), S145N[a] (1), Y159F[b] (1), K160T[b] (1), P194L[b] (2), Q197R[b] (1), S198A[b] (1), I48T[c] (1), D53N[c] (1), **G275S[c] (1), K276R[c] (1),** K278N[c] (1), H311Q[c] (1), S312N[c] (1), S96N[d] (2), 203T[d] (2), D225N[d] (1)	G142R[a] (1), S144N[a] (1), S145N[a] (1), Y159F[b] (1), P194L[b] (1), S198A[b] (1), I48T[c] (1), **G275S[c] (1), K276R[c] (1),** K278N[c] (1), H311Q[c] (1), S312N[c] (1), K121N[d] (1), K171N[d] (1), 225N[d] (1)	K131T[a] (1), K142R[a] (1), S144N[a] (1), S145N[a] (1), Y159F[b] (1), P194L[b] (1), S198A[b] (1), I48T[c] (1), **G275S[c] (1), K276R[c] (1),** K278N[c] (1), 300I[c] (1), H311Q[c] (1), S312N[c] (1), S96N[d] (1), D225N[d] (1), Q261R[e] (1)	S138A[a] (1), G142R[a] (1), K144S[a] (1), S145N[a] (1), A128T[b] (1), S159F[b] (1), N190D[b] (1), S193F[b] (1), S198A[b] (1), I48T[c] (1), **G275S[c] (1), K276R[c] (1),** K278N[c] (1), S312N[c] (1), D225N[d] (1), 246N[d] (1), N91S[e] (1)	K131T[a] (1), G142R[a] (1), S144N[a] (1), S145N[a] (1), Y159F[b] (1), I186G[b] (1), S198A[b] (1), I48T[c] (1), **G275S[c] (1), K276R[c] (1),** K278N[c] (1), S312N[c] (1), K121N[d] (1), K171N[d] (1), F219S[d] (1), D225N[d] (1), G62E[e] (1), R92K[e] (1)	F137S[a] (1), S138A[a] (1), G142R[a] (1), S144N[a] (1), S145N[a] (1), A128T[b] (1), K135T[b] (1), Y159F[b] (1), I160K[b] (1), V186G[b] (1), S193F[b] (1), S198A[b] (1), I48T[c] (1), **G275S[c] (1), K276R[c] (1),** K278N[c] (1), S312N[c] (1), K121N[d] (1), K171N[d] (1), G62E[e] (1), R92K[e] (1)
	50-<65 (N=1)	**-**	**-**	**-**	**-**	**-**	**-**
	≥ 65 (N=0)	**-**					
	No data (N=1)	Same as above	**-**	**-**	**-**	**-**	**-**
	HA not recovered (N= 19)	**-**	**-**	**-**	**-**	**-**	**-**
**Antigenic drift by geographical region**							
Region	Central (N=80)	**N122D[a] (3), S124N[a] (1), T128A[b] (11), T135K[b] (2), A138S[a] (6), I140K[a] (9),** R142G[a] (11), **R142K[a] (4), S144N[a] (13),** S144K[a] (1), **S145N[a] (2), S146G[a] (1), H156Q[b] (1), L157S[b] (1), Y159F[b] (7), Y159S[b] (6),** K160T[b] (11), P194L[b] (24), **A196T[b] (2),** Q197R[b] (4), **S198A[b] (2),** S198P[b] (6), **I48T[c] (2), D53E[c] (2),** D53N[c] (6), **G275S[c] (1), K276R[c] (1), K278N[c] (2),** H311Q[c] (17), **S312N[c] (8),** S96N[d] (23),	**N122D[a] (2), A138S[a] (5), I140K[a] (5), G142R[a] (7), S144N[a] (7), S145N[a] (2), S146G[a] (1), T128A[b] (5), T135K[b] (2), H156Q[b] (1), Y159F[b] (2), Y159S[b] (5),** K160T[b] (5), P194L[b] (12), **A196T[b] (2), S198A[b] (2), S198P[b] (5), I48T[c] (2), D53E[c] (1),** D53N[c] (1), **G275S[c] (1), K276R[c] (1), K278N[c] (2), H311Q[c] (10), S312N[c] (7),** K121N[d] (7), K171N[d] (7), **I214T[d] (2), 225N[d] (2),** 225D[d] (10),	**N122D[a] (2),** K131T[a] (12), **A138S[a] (5), I140K[a] (5), K142R[a] (7), K142G[a] (5), S144N[a] (7), S145N[a] (2), S146G[a] (1), T128A[b] (5), T135K[b] (2), H156Q[b] (1), Y159F[b] (2), Y159S[b] (5),** K160T[b] (5), P194L[b] (12), **A196T[b] (2), S198A[b] (2), S198P[b] (5), I48T[c] (2), D53E[c] (1),** D53N[c] (1), **G275S[c] (1), K276R[c] (1), K278N[c] (2),** 300I[c] (12), **H311Q[c] (10), S312N[c] (7),** S96N[d] (12), **N121K[d] (5), N171K[d] (5), I214T[d] (2),**	**N122D[a] (2),** S138A[a] (7), **I140K[a] (5), G142R[a] (7),** K144S[a] (5), **K144N[a] (7), S145N[a] (2), S146G[a] (1),** A128T[b] (7), **T135K[b] (2), H156Q[b] (1), S159F[b] (2),** S159Y[b] (5), K160T[b] (5), N190D[b] (12), S193F[b] (12), **A196T[b] (2), S198A[b] (2), S198P[b] (5), I48T[c] (2), D53E[c] (1),** D53N[c] (1), **G275S[c] (1), K276R[c] (1), K278N[c] (2),** Q311H[c] (2), **S312N[c] (7), N121K[d] (5), N171K[d] (5), I214T[d] (2), D225N[d] (2), 246N[d]**	**N122D[a] (2),** K131T[a] (12), **A138S[a] (5), I140K[a] (5), G142R[a] (7), S144N[a] (7), S145N[a] (2), S146G[a] (1), T128A[b] (5), T135K[b] (2), H156Q[b] (1), Y159F[b] (2), Y159S[b] (5),** K160T[b] (5), I186G[b] (12), **A196T[b] (2), S198A[b] (2), S198P[b] (5), I48T[c] (2), D53E[c] (1),** D53N[c] (1), **G275S[c] (1), K276R[c] (1), K278N[c] (2),** Q311H[c] (2), **S312N[c] (7),** K121N[d] (7), K171N[d] (7), **I214T[d] (2),** F219S[d] (12),	**N122D[a] (2),** F137S[a] (12), S138A[a] (7), **I140K[a] (5), G142R[a] (7), S144N[a] (7), S145N[a] (2), S146G[a] (1),** A128T[b] (7), K135T[b] (10), **H156Q[b] (1), Y159F[b] (2), Y159S[b] (5),** I160T[b] (5), **I160K[b] (7),** V186G[b] (12), S193F[b] (12), **A196T[b] (2), S198A[b] (2), S198P[b] (5), I48T[c] (2), D53E[c] (1),** D53N[c] (1), **G275S[c] (1), K276R[c] (1), K278N[c] (2),** Q311H[c] (2), **S312N[c] (7),** K121N[d] (7), K171N[d] (7),

		N121K^{d} (7), N171K^{d} (6), 203T^{d} (24), **K207Q^{d} (1), I214T^{d} (3), D225N^{d} (7), E62K^{e} (1), Q75H^{e} (2), G78D^{e} (2), K83E^{e} (1), S91N^{e} (1),** K92R^{e} (4), **Y94H^{e} (2), S262N^{e} (1)**	**Q75H^{e} (1), G78D^{e} (2), K83E^{e} (1), K92R^{e} (3), Y94H^{e} (2)**	**D225N^{d} (2), Q75H^{e} (1), G78D^{e} (2), K83E^{e} (1), K92R^{e} (3), Y94H^{e} (2),** Q261R^{e} (12)	**(12), Q75H^{e} (1), G78D^{e} (2), K83E^{e} (1),** N91S^{e} (12), **K92R^{e} (3), Y94H^{e} (2)**	**D225N^{d} (2),** G62E^{e} (12), **Q75H^{e} (1), G78D^{e} (2), K83E^{e} (1),** R92K^{e} (9), **Y94H^{e} (2)**	**I214T^{d} (2),** N225D^{d} (10), G62E^{e} (12), **Q75H^{e} (1), G78D^{e} (2), K83E^{e} (1),** R92K^{e} (9), **Y94H^{e} (2)**
	Eastern (N=7)	R142G^{a} (1), S144K^{a} (1), K160T^{b} (2), P194L^{b} (2), S198P^{b} (1), H311Q^{c} (1), S96N^{d} (2), N121K^{d} (2), N171K^{d} (1), 203T^{d} (2), **E62G^{e} (1),** K92R^{e} (1)	-	-	-	-	-
	Northwest (N=3)	-	-	-	-	-	-
	Western (N=9)	R142K^{a} (1), K160T^{b} (1), P194L^{b} (1), Q197R^{b} (1), D53N^{c} (1), S96N^{d} (1), 203T^{d} (1)	**G142K^{a} (1),** K160T^{b} (1), P194L^{b} (1), **Q197R^{b} (1),** D53N^{c} (1), K121N^{d} (1), K171N^{d} (1), 225D^{d} (1)	K131T^{a} (1), K160T^{b} (1), P194L^{b} (1), **Q197R^{b} (1),** D53N^{c} (1), 300I^{c} (1), S96N^{d} (1), Q261R^{e} (1)	S138A^{a} (1), **G142K^{a} (1),** K144S^{a} (1), A128T^{b} (1), S159Y^{b} (1), K160T^{b} (1), N190D^{b} (1), S193F^{b} (1), **Q197R^{b} (1),** D53N^{c} (1), Q311H^{c} (1), N91S^{e} (1)	K131T^{a} (1), **G142K^{a} (1),** K160T^{b} (1), I186G^{b} (1), **Q197R^{b} (1),** D53N^{c} (1), Q311H^{c} (1), K121N^{d} (1), K171N^{d} (1), F219S^{d} (1), G62E^{e} (1), R92K^{e} (1)	F137S^{a} (1), S138A^{a} (1), **G142K^{a} (1),** A128T^{b} (1), K135T^{b} (1), I160T^{b} (1), V186G^{b} (1), S193F^{b} (1), Q197R^{b} (1), D53N^{c} (1), Q311H^{c} (1), K121N^{d} (1), K171N^{d} (1), N225D^{d} (1), G62E^{e} (1), R92K^{e} (1)
	HA not recovered (N= 19)	-	-	-	-	-	-

SH= Southern hemisphere vaccine. NH= Northern hemisphere vaccine. SNH= vaccine shared for both Southern and Northern hemispheres. A(H3N2) HA1 antigenic sites are A=a, B=b, C=c, D=d, and E=e as described by Wiley and Skehel, 1987. The frequency of an amino acid substitution observed at a given antigenic site in Uganda HA1 proteins is reported as (n). The substitutions in **BOLD** are unique between subgroups relative to the vaccine of a given season. Amino acid substitutions against the NH vaccine strains are coloured brown for visualization purposes only. Recommended vaccines for each influenza season are adopted from the World Health Organization (WHO, https://www.who.int/teams/global-influenza-programme/vaccines/who-recommendations).

Supplementary Table 4 (continued): Antigenic drift among successfully sequenced Uganda A(H3N2) viruses (N=99) from 2010-2020 season vaccine viruses, recommended for the Southern (SH), Northern (NH) hemisphere or both (SNH), by cases, gender, age, and geographical region. Hemagglutinin (HA) sequences were not recovered for 19 H1N1pdm09 viruses.

Supplementary Table 5: Amino acid substitutions (AASs) in the complete HA, NA, and MP protein sequences of Uganda A(H1N1)pdm09 viruses compared to Southern and Northern Hemisphere vaccines viruses

Season	Vaccine: Passage, Genetic clade (Hemisphere)	No. of Uganda viruses analysed *(year sampled)*	Total unique AAS; Average AAS per gene	Mean AA identity (%) *(range)*	Virulence	Strong/Mild drug resistance	Host specificity shift	Antigenic drift/ Escape mutant	Creates/Removes potential glycosylation site	Structural protein interactions: host cell receptor binding, binding small ligand(s), viral oligomerization interfaces, and antibody recognition sites	Other
**Hemagglutinin (H1)**											
2010 - 2016	**A/California/7/2009:** Clade 1 (SNH)	77 *(2010 - 2016)*	78; 11.12	97.85 *(96.99-98.94)*	E391K:61 (14,886)	P100S[+]:76 (24,202)	R269K:1(7), A151T:10(259), D239E:4(669)	S200P:10(4,314), N142D:1(382), S145P:3(376), S207V:23(1), K180I:5(122), K180Q:12(10,222)		N458K:1(59), A13T:1(8119), A203T:1(491), A214T:1(1673), A273T:12(10192), A90T:1(39), D103E:1(47), D103N:1(76), D114N:58(12545), D518E:1(83), D52N:1(111),, E373A:1(132), E516K:58(10435), E516Q:1*, F12L:1(162), G56R:1(45), H143Y:2(80), H155Q:1(223), H155R:24 (92), H290Y:1(92), I166M:1(8), I233V:7(394), I284T:4 (27), I303L:1(29), I303V:7(154), I303X:1*, I341V:2(38), I392V:1(43), I435V:2(83), I517V:1(3), I74T:1(13), K180I:17(122), K180Q:12(10222), K300E:44(10978), K419R:2(31),, K475E:1(17), K475R:1(27), M274V:1(28), N146D:1(997), N245S:2(15), N458K:1(59), P135X:1*, P199Q:1(21), P288S:1(175), R222K:9(979), R62K:1(211), S101I:1(73), S101N:1(8254), S160G:2(1209), S202T:54(12381), S468N:54(11516), S86P:2(154), T137A:1(813), T258K:3(4), V251I:10(721), V289A:2(227), V289I:15(194), V36I:3(256), V444I:2(34), V47X:1*, V64I:1(184)	I338V[@]:75(22,922), P100S[@]:76(24,062), S220T[@]:77(22,514), I533V:1(13), L58I:1(4), L8M:1(389), R526K:23(67), V266L:33(491), V537A:24(1303), V6A:1(200)
2017-2018	**A/Michigan/45/2015:** E3, Clade 6B.1 (SNH)	30 *(2017- 2018)*	15; 4.87	99.09 *(98.76-99.65)*			R240Q[+]:30 (8,199)	K53N[+]:1(10), K159R:1(43), R240Q:30(8,199)	T295X[R]:1*	A278D:1(12), I113V:3(24), I303V:1(84), I312V:27(4637), K516E:1(50), N472S:1(32), S181T:11(4546), S91R:27(4628), T137A:23(724), V36I:1 (32)	R526G:1(1)
2019	**A/Michigan/45/2015**: E3, Clade 6B.1 (SH)	11 *(2018)*	8; 5.18	99.07 *(98.94-99.29)*			R240Q[+]:11(8,199)	R240Q:11(8,199)	T295X[R]:1*	I303V:1(84), I312V:11(4637), N472S:1(32), S181T:11(4546), S91R:11(4628), T137A:10(724)	
	**A/Brisbane/02/2018**: E3, Clade 6B.1A.1 (NH)		9; 6.18	98.9 *(98.76-99.12)*			R240Q[+]:11(4,003)	P200S:11(501), R240Q:11(4,003)	T295X[R]:1*	A299P:11(3501), G62R:11(3430), I303V:1(66), N472S:1(31), T137A:10(687), V315I:11(3519)	
**Neuraminidase (N1)**											

**2010-2016**	A/California/7/2009; Clade1 (SNH)	2010 - 2016 (n=76)	62; 7.7	98.04 *(96.8-99.57)*	**I365T:1**(335)	**Y155H[S]:2**(63), **V267A[S+]:1**(13), **I117M[M]:2**(325), **N248D[+]:75**(17,494), **N248X:1***, **V106I[+]:32**(9,341)		**I365T:1**(335), **A343T:1**(4), **N200S:44**(8,216), **K432E:27** (7,632), **N369K:59**(10,324)	**A343T:1**(4), **N44S[C]:45**(9,073)(8,932), **P272T[C]:2**(2), **N386K[R]:23**(7,307)	**A76T:1**(62), **A86V:1**(90), **C49Y:1**(4), **F115L:1**(14), **F74S:1**(868), **G382R:2**(49), **G454S:1**(61), **I321V:27**(7785), **I389K:9**(477), **I389T:1**(74), **K262R:2**(18), **K84E:1**(7), **K84R:5**(16), **L85I:1**(184), **N141S:1**(12), **N270K:1**(6648), **N397K:3**(458), **P93H:1**(520), **Q313R:9**(174), **R220K:9**(199), **S299A:1**(166), **S339L:2**(91), **S339P:1**(50), **T452I:5**(947), **V166I:14**(71), **V264I:1**(6530), **V394I:27**(367), **V448L:1**(4), **V453X:1***, **Y353X:1***	**N248D[@]:75**(17,352), **V106I[@]:32**(9,340), **A20T:1**(43), **A20V:5**(23), **E47G:1**(65), **I314M:5**(6320), **I34V:27**(7468), **I359M:1**(11), **I46T:1**(135), **I46V:1**(39), **L40I:24**(7058), **N42D:1**(32), **N71X:1***, **Q43K:2**(117), **Q45H:2**(71), **S442G:1**(5), **S82P:3**(591), **T9I:2**(69), **V13I:1**(6574), **V241I:59**(10391)
**2017-2018**	**A/Michigan/45/2015:** E3, Clade 6B.1 (SNH)	2017-2018 (n=30)	20; 6.5	98.47 *(98.08-99.79)*	**I365T:23**(186)			**I365T:23**(186), **S366I:1**(4), **I396V:1**(26)		**A75V:1**(22), **D416N:24**(1566), **G454D:1**(10), **G77R:27**(3978), **I108M:1**(2), **I188T:27**(3819), **K260E:1**(3), **N449D:27**(4082), **P93H:1**(520), **L412S:1***, **S366I:1**(4), **T72I:24**(311), **V234I:3**(12)	**Q45P:1***, **T16A:1**(9), **T362I:1**(3), **V80M:1**(497), **V81A:27**(3936)
**2019**	**A/Michigan/45/2015:** E3, Clade 6B.1 (SH)	2018 (n=11)	16; 7.73	98.34 *(98.08-98.51)*				**I365T:10**(186), **S366I:1**(4)		**A75V:1**(22), **D416N:11**(1566), **G454D:1**(10), **G77R:11**(3978), **I108M:1**(2), **I188T:11**(3819), **K260E:1**(3), **N449D:11**(4082), **S366I:1**(4), **T72I:11**(311)	**Q45P:1***, **T16A:1**(9), **T362I:1**(3), **V80M:1**(497), **V81A:11**(3936)
	**A/Brisbane/02/2018:** E3, Clade 6B.1A.1 (NH)		13; 4.73	98.97 *(98.72-99.15)*				**I365T:10**(161), **S366I:1**(2)		**A75V:1**(17), **D416N:11**(1511), **G454D:1**(7), **I108M:1**(1), **K260E:1**(2), **S366I:1**(2), **T72I:11**(276)	**Q45P:1***, **T13I:11**(3647), **T16A:1**(5), **T362I:1***, **V80M:1**(469)
**Matrix Protein (M1 and M2)**											
**2010-2016**	A/California/7/2009; Clade1 (SNH)	2010 - 2016 (n=79)	16; 2.7	98.95 *(97.73-100)*			**N133S:1**(43), **E16G:1**(52), **G89D:1**(14)			**A33T:1**(9), **D21G:50**(6871), **D21V:2**(323)	**A227T:1**(187), **K230R:45**(7509), **M192V:45**(7395), **Q208K:1**(6028), **T167A:1**(74), **V80I:54**(8507), **E14G:4**(448), **R18K:6**(98), **T11I:1**(77), **Y76F:1**(2)
**2017-2018**	**A/Michigan/45/2015:** E3, Clade 6B.1 (SNH)	2017-2018 (n=30)	4; 0.23	99.86 *(97.73-100*						**F55L:1**(12), **I28F:3**	**E204X:1***, **M203I:1**(7)
**2019**	**A/Michigan/45/2015:** E3, Clade 6B.1 (SH)	2018 (n=11)	1; 0.09	99.95 *(98.86-100)*						**F55L:1**(12)	
	**A/Brisbane/02/2018:** E3, Clade 6B.1A.1 (NH)	2018 (n=11)	2; 1.09	99.75 *(99.6-100)*						**F55L:1**(2)	**X167T:11***

Data are in n or n (%), unless otherwise indicated. AA= Amino acid. AAS= Amino acid substitutions. SH= Southern hemisphere vaccine. HN= Northern hemisphere vaccine. SNH= vaccine strain shared by both the Southern and Northern hemispheres for a given influenza season. Vaccine virus passage history indicated as indicated by FluSurver. The number of unique amino acid substitutions observed in all proteins sequences and per protein sequence are represented as N; n. The number of times a substitution is observed in the Uganda virus proteins and globally is reported in **bold** and (bracket) if available, respectively. Substitutions are colour-coded based on their known or predicted level of biological relevance. **Red** substitutions are the most significant (interestlevel =3) as they alter virulence, cause strong drug resistance and reverse premature stop codon in PB1-F2. The **Orange** (significant, interestlevel=2) occur at drug binding sites, affect host specificity and cause antigenic shift and mild drug resistance**. Magenta (**significant, interestlevel=2) adds or removes glycosylation sites. **Blue** substitutions (moderately significant, interestlevel=1) are involved at sites of interaction such as host cell receptor binding, binding small ligand(s), viral oligomerization interfaces, and antibody recognition sites**.** The **Green** substitutions (least significant, interestlevel=0) are common to subtypes while Black (least significant with warnlevel=0) have no known effects. Superscript symbol definitions: **“*” =** AAS reported for the first time (current global frequency = 0). “+” = AAS have their function reported in combination with others**. “R”** = AAS removes a potential glycosylation site**. “C”** = AAS creates a potential glycosylation site. **“S”** = AAS causes strong drug resistance. **“M”** = AAS causes mild drug resistance**. “@”** = AAS is a common subtype marker**.** Amino acid substitutions with multiple functions reported were indicated under all functions as appropriate. For substitutions observed for the first time (global frequency=0), their biological relevance (functions) were reported based on the functions of their equivalent positions in the resolved protein structures.

Supplementary Table 5: Amino acid substitutions in the complete protein sequences of HA (H1), NA (N1) and MP (M1 and M2) of Uganda A(H1N1)pdm09 viruses compared to Southern and Northern Hemisphere vaccines. Amino acid similarity between protein sequences, amino acid substitutions and their corresponding global frequencies and functions were obtained from Flusurver (http://flusurver.bii.a-star.edu.sg; accessed on 24th September 2021). The highest number of unique substitutions in the H1 was 78, 62 in N1, and 16 in MP observed in 2010-2016 viruses against the A/California/7/2009 vaccine strain.

Supplementary Table 6: Amino acid substitutions (AASs) in the complete HA, NA, and MP protein sequences of Uganda A(H3N2) viruses compared to Southern and Northern Hemisphere vaccines viruses

Season	Vaccine: Passage, Genetic clade (Hemisphere)	No. of Uganda viruses analysed *(year sampled)*	Total unique AAS; Mean number of AAS per gene (total substitutions/number of sequences)	Mean AA similarity (%) *(range)*	Virulence & Host specificity shift	Virulence & Antigenic drift / Escape mutant	Antigenic drift / Escape mutant	Strong/Mild drug resistance	Host specificity shift	Creates/Removes potential glycosylation site	Structural protein interactions: host cell receptor binding, binding small ligand(s), viral oligomerization interfaces, and antibody recognition sites	Other
						**Hemagglutinin (H3)**						
**2010-2011**	**A/Perth/16/2009:** A/Perth/16 clade (SNH)	25 *(2010 – 2011)*	37; 9.72	98.13 *(96.29-98.94)*	**A154S:1** (3,152)	**A214S:15**(20,232), **A214P:1**(769), **N161S:24** (205,17)	**I156K[+]:1**(97), **K160N:25**(8,203), **R158G:1**(7,817), **K78E:25** (21,726)		**S230I:25**(24,366), **K99E[+]:1** (168), **A212T:1**(6), **I208V:3**(53), **T144A[+]:1** (5,294), **F175S:1**(2,979), **N241D:1** (16,616)	**K160N[C]:25**(8,203), **T144A[R]:1**(5,294), **S61N[C]:7**(20,569)	**A492T:3**(71), **D503N:15**(698), **Y9C:1**(2141), **D69N:1**(2328), **G291S:2**(23), **I258V:1**(139), **I422V:2**(4959) **I434V:1**(62), **K280R:1**(58), **K2M:1***, **L199H:25**(24599), **N294K:1**(20201), **N328S:15**(20443), **T64I:1**(20194), **P305S:2**(35), **Q49R:1**(19595), **R285K:2**(87), **T228A:25**(22524), **V239I:16**(21153)	**I538M:1**(189)
**2012**	**A/Perth/16/2009:** A/Perth/16 clade (SH)	16 *(2012)*	28; 11.13	97.83 *(96.82-98.76)*		**A214S:8**(20,232), **N161S:8** (205,17)	**I156K[+]:1**(97), **K160N:16**(8,203), **R158G:5**(7,817), **K78E:16** (21,726)		**S230I:13**(24,366), **T144A[+]:5** (5,294), **S230V:3** (30)	**K160N[C]:16**(8,203), **S140N[R]:1**(145), **S61N[C]:13**(20,569), **T144A[R]:5** (5,294)	**D503N:3**(698), **D505N:2**(15949), **Y9C:5**(2141), **I258V:1**(139), **K280R:8**(58), **T64I:5**(20194), **L199H:16**(24599), **L19V:1**(9), **N137K:1**(5034), **N294K:5**(20101), **N328S:8**(20443), **Q49R:5**(19595), **S25R:1**(6), **S278N:3**(336), **T228A:16**(22524), **V14G:1**(57), **V239I:8**(21153)	
	**A/Victoria/361/2011:** Clade 3C.1 (NH)		27; 9.75	98.26 *(97.88-98.41)*		**S214A:8**(778), **N161S:8** (20,312)	**I156K[+]:1**(90), **Q172H:16**(21,478), **V202G:16**(21,412), **R158G:5**(7,721)		**I230V:3** (26)	**N61S[R]:3**(1,230), **S140N[R]:1**(126), **T144A[R]:5** (5,288)	**D503N:3**(520), **D505N:2**(15778), **H9C:5**(2129), **H9Y:11**(18463), **I239V:9**(678), **I64T:11**(1384), **K280R:8**(55), **L19V:1**(6), **N137K:1**(5023), **N294K:5**(20024), **Q49R:5**(19541), **S25R:1**(5), **S278N:3**(316), **S328N:11**(1241), **V14G:1**(54), **Y235S:16**(20668)	
**2013**	**A/Victoria/361/2011:** Clade 3C.1 (SH)	6 *(2013)*	21; 11	97.99 *(97.35-98.41)*		**L173S:1**(710), **N161S:8** (20,312)	**I156K[+]:1**(90), **Q172H:6**(21,478), **V202G:6**(21,412), **N138S:1**(31), **R158G:6**(7,721)	**S107G[M]:1**(11), **N138S[M]:1**(31)	**I230T:1**(200), **Q213H:1**(240),	**S140N[R]:1**(126), **T144A[R]:6** (5,288)	**D69N:1**(1223), **H9Y:6**(18463), **I422V:1**(4955), **L19V:1**(6), **N294K:6**(20024), **Q49R:6**(19541), **V313I:1**(19), **V363M:1**(1382), **Y235S:6**(20668)	
	**A/Texas/50/2012**: E5, Clade 3C.1 (NH)		18; 8	98.49 *(97.88-98.94)*		**P214S:6**(19,232), **L173S:1**(709), **N161S:6**(19,737)	**V202G:6** (20,336), **I156K[+]:1**(90), **N138S:1**(30), **R158G:6**(7,663)	**S107G[M]:1**(10), **N138S[M]:1**(30)	**I230T:1**(197), **Q213H:1**(240), **T144A[+]:6**(5,281)	**N138S[R]:1**(30), **S140N[R]:1**(122)	**D69N:1**(962), **F235S:6**(19566), **I422V:1**(4952), **L19V:1**(6), **V313I:1**(13) **V363M:1**(1343)	

**2014**	**A/Texas/50/2012**: E5, Clade 3C.1 (SNH)	11 *(2014)*	17; 8	98.56 *(96.23-98.76)*	**A154S:1**(3,132)	**P214S:10**(19,232), **N161S:11**(19,737)	**I156K^{+}:1**(90), **V202G:11**(20,336), **E78K:1**(562), **R158G:11**(7,663)		**F175S:1(**2,973), **N241D:1** (16,534), **N144A^{+}:11**(5,281)		**A16T:3**(432), **F235S:11**(19596), **I10V:1**(47), **K342R:1**(2804), **L19I:1**(15632), **L19V:6**(6), **N137D:1**(244)	
**2015**	**A/Switzerland/9715293/2013**: E4/E2, clade 3C.3a (SNH)	14 *(2015)*	40; 11.57	97.76 *(97.17-99.29)*	**L173S:1** (599), **S154A:13**(14,309)		**G158K:2**(4,110), **G158R:4**(6,837), **V202G:14**(17,342), **I156I^{+}:8**(16,900), **I156K^{+}:6**(77) **N160K:1**(2,812), **N160S:6**(12,395), **K176T:6**(12,943)	**S107N^{M+}:1**(2,099)	**D241N:7**(928), **I230T:1** (189), **Q213R:2**(1,111), **S175F:7** (1,050), **S175Y:6** (13,393), **K176T:6** (12,943)	**K176T^{C}:6** (12,943), **A144T^{C}:6**(13,268), **N160K^{R}:1**(2,812), **N160S^{R}:6**(12,395), **S140N^{R}:1**(79), **S140R^{R}:1** (24)	**A122T:1**(50), **D307N:1**(19), **D505N:7**(15149), **D69N:3**(576), **F15S:1**(71), **G65S:1**(23), **L19F:1**(5), **L19I:7**(15473), **L19V:4**(6), **L443I:1**(8), **M184V:2**(37), **N187H:1***, **N461S:1**(8), **Q327H:6**(10839), **R342K:13**(14234), **S25N:1**(106), **S278N:1**(295), **S281G:1**(8), **X235S:14***, **V104I:1**(89), **Y9C:1**(2123)	
**2016-2017**	**A/Hong Kong/4801/2014**: E5/E2, clade **3C.2a** (SNH)	27 *(2016-2017)*	67; 11.85	97.73 *(96.82-98.94)*	**A154S:1**(3,053)	**S214A:2**(56), **S214P:7**(721), **S161N:2**(135), **L173S:1**(561)	**H172Q:1**(20), **R158K:5**(4,107), **I156K^{+}:9**(73), **S160N:13**(1,585), **N138D:1**(813), **E78G:1**(1,492), **E78K:1**(556), **R158G:12**(6,183), **S160K:2**(2,812), **K176T:14**(12,910)	**S107N^{M+}:1**(2,099), **N138D^{M}:1**(813)	**K99E^{+}:1** (129), **A212T:2**(1), **D241N:7**(667), **I230T:3** (187), **Q213R:5**(1,111), **Y175F:7** (774), **Y175S:6** (2,916), **T144A^{+}:11** (3,861), **K176T:14**(12,910)	**T56K^{R}:1***, **S160N^{C}:13**(1,585), **N138D^{R}:1**(813), **S140N^{R}:1**(76), **T144A^{R}:11** (3,861), **T151K^{R}:2**(1,734)	**A492T:1**(45), **C12R:1**(8), **D509E:1**(4), **D69E:2**(89), **D69N:7**(556), **G291S:1**(14), **G495E:2**(1139), **G500E:1**(4620), **G94D:2**(167), **H327Q:18**(6034), **I19L:10**(1585), **I19V:3**(6), **I239V:2**(49), **I41V:1**(50), **I422V:7**(4946), **I50V:1**, **I64T:2**(122), **K108R:5**(2729), **K223Q:1**(15), **K275R:1**(29), **P210L:27**(17061), **K292R:1**(24), **K294N:2**(97), **K342R:6**(2776), **K519R:1**(16), **N137K:9**(4986), **N187K:7**(5198), **N461S:5**(8), **R49Q:2**(358), **N505D:12**(1319), **V14G:2**(44), **Q91H:2**(139), **R421G:1***, **S162G:1**(9), **S112N:26**(16974), **S278N:1**(293), **S328N:8**(487), **V363M:1**(887), **Y110H:2**(266)	**I538M:2**(165)
**2018**	**A/Singapore/INFIMH-16-0019/2016**: E5/E1, Clade **3C.2a1** (SNH)	13 *(2017)*	50; 14.38	97.01 *(95.94-98.76)*	**A154S:5**(2,275)	**S214A:2**(33), **S214P:5**(237), **S161N:2**(49)	**G158K:1**(3,675), **G158R:7**(3,133), **H172Q:1**(9), **S160N:7**(119), **N138D:2**(340), **K176T:6** (9,104), **I156K^{+}:5**(31)	**N138D^{M}:2**(340)	**A212T:2**(1), **D241N:2**(18), **I230T:2** (149), **Q213R:1**(719), **Y175F:2** (44), **K99E^{+}:1** (116), **T144A^{+}:5** (2,457), **K176T:7** (9,104), **Y175S:5** (2,168)	**S160N^{C}:7**(119), **N138D^{R}: 2**(340), **T144A^{R}:5** (2,457), **T151K^{R}:2**(1,705) **T56K^{R}:1***	**A492T:1**(17), **D69E:1**(88), **D69N:2**(419), **E495G:11**(10312), **E500G:8**(7151), **G291S:1**(7), **G94D:2**(152), **H327Q:10**(4665), **I19L:7**(134), **I239V:2**(40), **I41V:1**(29), **I64T:2**(40), **K108R:3**(2703), **K137N:8**(6402), **K187N:8**(6613), **K275R:1**(15), **K292R:2**(16), **K294N:2**(40), **K342R:5**(2161), **N461S:1**(4), **N505D:7**(120), **P210L:13**(11545), **Q91H:1**(127), **R421G:1***, **R49Q:2**(260), **S162G:1**(6), **S328N:7**(119), **V422I:8**(6819), **Y110H:2**(137)	**I538M:2**(151)

**2019**	**A/Switzerland/8060/2017**: E5/E1, Clade 3C.2a2 (SH)	13 *(2017)*	54; 16.08	97.04 *(96.29-98.59)*	**A154S:5**(1,938)	**S214A:2**(22), **S214P:5**(76), **S161N:2**(25)	**H172Q:1**(3), **K147T:13** (2,515), **K158R:7** (239), **I156K**$^{+}$**:5**(27), **S160N:7**(11), **N138D:2**(47) **K158G:5** (3,050), **K176T:6**(3,244)	**N138D**^{M}**:2**(47)	**A212T:2***, **D241N:2**(1), **I230T:2** (95), **Q213R:1**(429), **Y175F:2** (24), **K99E**$^{+}$**:1** (113), **Y175S:5**(1,846), **K176T:6**(3,244)	**T144A**^{R}**:5** (2,124), **T151K**^{R}**:2**(528) **T56K**^{R}**:1***	**A492T:1**(10), **D69E:1**(2), **D69N:2**(134), **G291S:1**(6), **G495E:2**(77), **G500E:5**(1016), **G94D:2**(47), **H327Q:10**(3210), **I19L:7**(24), **I239V:2**(23), **I41V:1**(15), **I422V:5**(1355), **I64T:2**(24), **K108R:3**(1485), **K275R:1**(6), **K292R:1**(9) **K294N:2**(24), **K342R:5**(1839), **N137K:5**(1425), **N187K:5**(1384), **N461S:1**(2), **N505D:7**(23), **P210L:13**(5215), **Q277R:13**(3206), **Q91H:1**(21), **R421G:1***, **R49Q:2**(199), **S112N:13**(5185), **S162G:1**(6), **S328N:7**(27), **Y110H:2**(66)	**I538M:2**(129)
	**A/Kansas/14/2017**: E5, Clade 3C.3a (NH)		57; 18.46	96.55 *(95.58-97.88)*	**S154A:8**(3,435)	**S214A:2**(22), **S214P:5**(76), **S209F:13**(3,398), **S161N:2**(25)	**G158K:1**(2,052), **G158R:7**(251), **H172Q:1**(3), **I156K**$^{+}$**:5**(27), **S160N:7**(11), **N138D:2**(50), **K160S**:6(3,409), **K176T:6**(3,404)	**N138D**^{M}**:2**(50), **N107S**$^{M+}$**:13**(3,513)	**N206D:13**(5,378), **A212T:2***, **D241N:2**(1), **I230T:2** (96), **Q213R:1**(432), **S175F:2** (44), **S175Y:6** (3,505), **K176T:6**(3,404), **A144T**$^{+}$**:8**(3,250), **K99E**$^{+}$**:1** (113)	**N138D**^{R}**:2**(50), **T151K**^{R}**:2**(541), **T262N**^{C}**:13**(5,382), **T56K**^{R}**:1***	**A492T:1**(10), **C9Y:13**(3471), **D69E:1**(2), **D69N:2**(136), **G291S:1**(6), **G495E:2**(80), **G500E:5**(1030), **G94D:2**(50), **I19L:7**(24), **I239V:2**(23), **I41V:1**(17), **I422V:5**(1369), **I64T:2**(24), **K108R:3**(1509), **K275R:1**(6), **K292R:1**(11), **K294N:2**(24), **M494I:13**(3562), **N137K:5**(1441), **N187K:5**(1400), **N461S:1**(2), **N505D:7**(23), **Q327H:3**(2157), **Q91H:1**(21), **R342K:8**(3539), **R421G:1***, **R49Q:2**(199), **S162G:1**(6), **S328N:7**(28), **Y110H:2**(69)	**I538M:2**(130)
**2020**	**A/South Australia/34/2019**: E5, Clade 3C.2a1b (SH)	13 *(2017)*	56; 18.69	96.36 *(95.23-98.41)*	**A154S:5** (1,252)	**S214A:2***, **S214P:5**(89), **S161N:2**(4)	**G158K:1**(2), **G158R:7**(13), **H172Q:1**(2), **I202G:13**(1,954), **K147T:13** (1,312), **I156K**$^{+}$**:5**(1), **S160N:7**(59), **N138D:2**(3), **K176T:6**(930), **G78E:13**(1093),	**N138D**^{M}**:2**(3)	**A212T:2***, **D241N:2***, **I230T:2** (9), **Q213R:1**(416), **Y175F:2***, **K99E**$^{+}$**:1** (122), **Y175S:5**(1,075)	**S160N**^{C}**:7**(59), **N138D**^{R}**:2**(3), **T144A**^{R}**:5** (1,288), **T151K**^{R}**:2**(252), **T56K**^{R}**:1***	**A492T:1***, **D69E:1**(2), **D69N:2**(46), **E500G:8**(1491), **F235S:13**(1467), **G291S:1**(1), **G495E:2**(8), **G78E:13**(1093), **G94D:2**(2), **I19L:7***, **I239V:2***, **I41V:1**(5), **I64T:2***, **K137N:8**(899), **K187N:8**(1077), **K275R:1***, **K292R:1**(1), **K294N:2**(2), **K342R:5**(1067), **M363V:13**(1437), **N461S:1***, **N505D:7***, **Q327H:3**(11), **Q91H:1**(16), **R108K:10**(1087), **R421G:1***, **R49Q:2**(82), **S162G:1**(2), **S328N:7**(2), **V422I:8**(1092), **Y110H:2***	**I538M:2**(117), **I545V:13**(1326)
**Neuraminidase (N2)**												
**2010-2011**	**A/Perth/16/2009**: A/Perth/16 clade (SNH)	25 *(2010 - 2011)*	32; 5.8	98.61 *(97.02-99.36)*			**E221D:1**(12,941), **K369T:23**(17,155), **S367N:23** (17,347)	**D251N**^{M}**:1**(16)		**N402D**^{R}**:24**(15,970), **S331R**^{R}**:11**(592)	**D127T:1***, **D339A:2**(1), **D93G:1**(14174), **G414D:1**(24), **I26T:3**(205), **I26V:1**(496),	**F42L:1**(33), **L81P:16**(15783), **M51V:1**(544),

											**I307M**:**1**(302), **I464L**:**23**(16912), **I77K**:**1**(10), **K249E**:**1**(244), **K308E**:**1**(47), **K369T**:**40**(17155) **L338S**:**1**(485), **Q273R**:**1**(201), **R60K**:**3**(86), **S334N**:**10**(97), **T95A**:**3**(25), **V143M**:**3**(511)	**N43H**:**1**(26), **N43Y**:**6**(114), **P55T**:**1**(4), **S44F**:**1**(40), **V317M**:**2**(5), **Y40C**:**2**(207)
**2012**	**A/Perth/16/2009**: A/Perth/16 clade (SH)	17 *(2012)*	19; 7.35	98.36 *(97.87-98.93)*			**E221K**:**1**(54), **E344K**:**1**(2,739), **K369T**:**17**(17,155)			**N329D**^{R}:**1**(35), **S367N**^{C}:**40**(17,347), **N402D**^{R}:**8** (15,970), **S331R**^{R}:**9**(592)	**E344K**:**1**(2,739), **K369T**:**17**(1,755), **D93G**:**5**(1,4174), **E343D**:**1***, **I26T**:**9**(205), **I307M**:**7**(302), **I464L**:**17**(16,912), **I73V**:**2**(14), **I77T**:**1**(158), **S315N**:**1**(28), **S334N**:**9**(97)	**L81P**:**8**(15,783), **N43H**:**9**(26), **V13I**:**2**(15)
	**A/Victoria/361/2011**: Clade 3C.1 (NH)		18; 6.82	98.11 *(97.66-99.57)*			**T329N**:**5**(9,543), **E221K**:**1**(45), **T329D**:**1**(28), **E344K**:**1**(2,738)			**T329N**^{C}:**5**(9,543), **D402N**^{C}:**9** (317)	**G93D**:**12**(1686), **E343D**:**1***, **I26T**:**9**(51), **I307M**:**7**(209), **I73V**:**2**(9), **I77T**:**1**(152), **K258E**:**17**(15998), **S315N**:**1**(21), **S331R**:**9**(558), **S334N**:**9**(92)	**N43H**:**9**(25), **P81L**:**9**(312), **V13I**:**2**(14)
**2013**	**A/Victoria/361/2011**: Clade 3C.1 (SH)	7 *(2013)*	9; 2.86	99.25 *(98.93-99.57)*			**T329N**:**5**(9,543), **T329S**:**2**(4,291)	**D251V**^{M}:**1**(311), **Y155F**^{S}:**1**(296)		**T329N**^{C}:**5**(9,543)	**G111A**:**1***, **K258E**:**7**(15998), **S315G**:**1**(288), **V165I**:**1**(6)	**M241L**:**1**(3)
	**A/Texas/50/2012**: E5, Clade 3C.1 (NH)		8; 2.14	99.39 *(99.15-99.79)*			**H150R**:**7**(14,990), **N329S**:**1**(4,286)	**D251V**^{M}:**1**(297), **Y155F**^{S}:**1**(296), **N329S**^{S}:**1**(4,286)		**N329S**^{R}:**1**(4,286)	**G111A**:**1***, **S315G**:**1**(288), **V165I**:**1**(6)	**M241L**:**1**(3)
**2014**	**A/Texas/50/2012**: E5, Clade 3C.1 (SNH)	11 *(2014)*	12; 2.18	99.39 *(98.93-99.79)*			**E221D**$^{+}$:**1**(12,843), **H150R**:**11**(14,990), **E344K**:**1**(2,738)	**H150R**$^{S+}$:**11**(14,990), **I106V**$^{M+}$:**1**(16)			**D127E**:**3**(20), **I392T**:**1**(660), **P126S**:**1**(38), **V143L**:**1**(8)	**A82T**:**1**(37), **I194V**:**1**(112), **V317M**:**1**(4), **Y40C**:**1**(38)
**2015**	**A/Switzerland/9715293/2013**: E4/E2, clade 3C.3a (SNH)	14 *(2015)*	19; 3.93	99.07 *(98.51-99.57)*			**D221E**:**6**(292), **E344K**:**5**(2,736)	**D251V**^{M}:**1**(195), **Y155F**^{S}:**1**(197), **S247T** $^{M+}$:**4** (10,132), **S245N**$^{C+}$:**2**(10,271)		**S245N**^{C}:**2**(10,271)	**E344K**:**5**(2,736), **I28L**:**1**(1), **I380V**:**7**(11,056), **P468H**:**1**(8,317), **S315G**:**1**(201), **S315N**:**1**(18), **S384F**:**1**(31), **T267K**:**7**(11,458), **T392I**:**13**(12,093), **V231I**:**1**(16), **V263I**:**1**(84)	**I194V**:**1**(104), **I30V**:**1**(131), **M51T**:**2**(3)
**2016-2017**	**A/Hong Kong/4801/2014**: E5/E3, clade **3C.2a** (SNH)	27 *(2016-2017)*	49; 7.89	98.10 *(97.43-99.36)*			**D221E**:**7**(215), **N329S**:**1**(4,283), **E344K**:**3**(2,735), **G346V**:**1**(289), **K220N**:**1**(1,604), **R400K**:**1**(80), **K220R**:**1**(2), **R150H**:**1**(178)	**D251V**^{M}:**1**(133), **Y155F**^{S}:**1**(133), **S247T** $^{M+}$:**10** (10,130), **R150H**$^{S+}$:**1**(178)		**N329S**^{R}:**3**(4,283), **D309N**^{C}:**1**(71), **D402N**^{C}:**2** (33), **N86H**^{R}:**1**(1), **S245N**$^{C+}$:**2**(10,268), **S331R**^{R}:**6**(527)	**D339N**:**6**(8101), **G286D**:**1**(13), **G93D**:**4**(747), **H347N**:**1**(4), **I176M**:**2**(2,614), **I231V**:**27**(12,770), **I26V**:**6**(443), **I307M**:**2**(168), **I380V**:**13**(11,054), **I65V**:**1**(15), **K249E**:**6**(223), **K431R**:**1**(5), **L338S**:**6**(440), **L464I**:**1**(27), **P126L**:**1**(1,278), **P468H**:**7**(8,316), **P468L**:**2**(558), **Q273R**:**6**(198), **S315G**:**1**(141), **S315N**:**1**(18), **T267K**:**14**(11,439), **T392I**:**27**(11,944), **T69A**:**1**(9), **T95A**:**7**(21)	**F23L**:**2**(48), **I30V**:**1**(128), **M51V**:**6**(517), **N161S**:**2**(1,934), **N43Y**:**2**(1), **P55T**:**1**(4), **P81L**:**2**(7), **P81S**:**1**(61), **V303I**:**2**(1469)
**2018**	**A/Singapore/INFIMH-16-0019/2016**:	13 *(2017)*	30; 9	97.28 *(96.38-99.79)*			**D221E**$^{+}$:**2**(4), **N329S**:**2**(4,267), **K220R**:**1**(2)	**N245S**$^{M+}$:**7**(257), **T247S** $^{M+}$:**7**(240)		**N329S**^{R}:**2**(4,267), **N245S**^{R}:**7**(257), **D402N**^{C}:**2** (16),	**G93D**:**2**(650), **H347N**:**1**(3), **H468P**:**7**(281), **I176M**:**2**(2604), **I26V**:**5**(71), **I212V**:**13**(8894),	**I30V**:**1**(14), **M51V**:**5**(91), **N43Y**:**2***,

	E5/E1, Clade **3C.2a1** (SNH)									N86H[R]:1(1), S331R[R]:5(135)	I307M:1(155), K249E:5(14), K267T:7(225), L338S:5(70), L464I:1(5), N339D:10(1062), Q273R:5(15), T95A:6(16), V380I:7(432)	P55T:1(4), P81L:2(3), P81S:1(52), V303I:1(1461)
**2019**	**A/Switzerland/8060/2017**: E5/E2, Clade 3C.2a2 (SH)	13 *(2017)*	31; 11.46	96.78 *(95.74-99.36)*			D221E[+]:2*, S329N:11(81), K220R:1(2)	N245S[M+]:7(28), T247S[M+]:7(11)		S329N[C]:11(81), N245S[R]:7(28), D402N[C]:2 (1), N86H[R]:1(1)	G93D:2(93), H347N:1(2), H468P:7*, I26V:1(5), I307M:1(89), K249E:5*, K267T:7(11), L338S:5(2), L464I:1(3), M176I:11(2932), N339D:10(33), Q273R:5(1), S331R:5(8), S386P:13(1595), T95A:6(4), V380I:7(114)	I30V:1(7), M51V:5(11), N43Y:2*, P55T:1(1), P81L:2(2), P81S:1(12), V194I:13(4235), V303I:1(1106)
	**A/Kansas/14/2017**: E5, Clade 3C.3a (NH)		35; 13.85	96.49 *(95.31-98.72)*			D221E[+]:2*, T329N:11(89), K220R:1(2), K344E:13(1,819), T329S:2(2,519)	T247S[M+]:7(12), N245S[M+]:7(29), A149V[M]:13(2,578), H155Y[S]:13(2,598)		T329N[C]:11(89), N245S[R]:7(29), D402N[C]:2 (1), N86H[R]:1(1)	G93D:2(102), H347N:1(2), H468P:7*, I140L:13(2592), I176M:2(1431), I26V:5(5), I307M:1(89), K249E:5*, K267T:7(12), L338S:5(2), L464I:1(3), N339D:10(36), Q273R:5(1), R75K:13(2623), S331R:5(8), T95A:6(4), V380I:7(117)	I30V:1(7), M51V:5(11), N43Y:2*, P55T:1(2), P81L:2(2), P81S:1(12), V303I:1(1103)
**2020**	**A/South Australia/34/2019**: E5, Clade 3C.2a1b (SH)	13 *(2017)*	33; 13.46	96.55 *(95.74-98.72)*			D221E[+]:2*, S329N:11(9), N220K:12(1,116), N220R:1(1), K344E:13(84)	T247S[M+]:7*, N245S[M+]:7(5)		S329N[C]:11(9), N245S[R]:7(5), D402N[C]:2 (3), N86H[R]:1*	G93D:2(85), H347N:1*, H468P:7(2), I176M:2(40), I26V:5(4), I307M:1(21), K249E:5*, K267T:7*, L126P:13(1105), L338S:5*, L464I:1(3), N339D:10(7), Q273R:5(1), R315S:13(1223), S331R:5(7), T95A:6(2), V380I:7(59)	I303V:12(1118), I30V:1*, M51V:5(4), N43Y:2*, P55T:1*, P81L:2(1), P81S:1(7)
**Matrix Protein (M1 and M2)**												
**2010-2011**	**A/Perth/16/2009**: A/Perth/16 clade (SNH)	25 *(2010 - 2011)*	6: 0.28	99.86 *(98.86-100)*					G16E:1(759)		P25Q:1(8), M93I:1(3), K98R:1, L40F:1*	D88E:1(26), R18K:2(43)
**2012**	**A/Perth/16/2009**: A/Perth/16 clade (SH)	17 *(2012)*	4; 0.82	99.55 *(98.86-100)*							F48S:1(217), K98R:1(6), R61G:3(13)	D88E:9(26)
	**A/Victoria/361/2011**: Clade 3C.1 (NH)		4; 0.82	99.55 *(98.86-100)*							F48S:1(210), K98R:1(5), R61G:3(15)	D88E:9(20)
**2013**	**A/Victoria/361/2011**: Clade 3C.1 (SH)	7 *(2013)*	4; 0.57	99.78 *(98.86-100)*							A33V:1(4), F48S:1(210), L54V:1(185)	V15I:1(48)
	**A/Texas/50/2012**: E5, Clade 3C.1 (NH)		4; 0.57	99.78 *(98.86-100)*							A33V:1(3), F48S:1(209), L54V:1(185)	V15I:1(47)

**2014**	**A/Texas/50/2012**: E5/E1, Clade 3C.1 (SNH)	12 *(2014)*	4; 0.83	99.33 *(96.59-100)*							**F48S**:**7**(209), **I39M**:**1**(16)	**E14G:1**(24), **T227A:1**(27)
**2015**	**A/Switzerland/9715293/2013**: E4/E2, clade 3C.3a (SNH)	14 *(2015)*	7; 1.21	99.14 *(97.73-100)*				**V27A**$^{SM+}$:**2**(22),			**F48S**:**7**(208), **I39M**:**3**(16), **L54V**:**1**(147)	**N20S:1**(33), **Q208R:1**(8), **T239A:2**(25)
**2016-2017**	**A/Hong Kong/4801/2014**: E5/E3, clade **3C.2a** (SNH)	29 *(2016-2017)*	15; 1.38	99.08 *(96.59-100)*				**V27A**$^{SM+}$:**6**(21),	**G16E**:**7**(483)		**D21G**:**1**(30), **F48S**:**10**(208), **L54V**:**1**(104), **L59I**:**1**(10), **R61K**:**1**(3)	**E235K:1**(8), **K242R:1**(1), **N13D:1**(1), **N20S:1**(32), **R18K:1**(35), **T227A:1**(5), **T227N:1**(5), **T239A:6**(23)
**2018**	**A/Singapore/INFIMH-16-0019/2016**: E5/E1, Clade **3C.2a1** (SNH)	14 *(2017)*	8; 1.79	98.49 *(96.59-100)*				**A27V**$^{SM+}$:**13**(6,981)	**G16E**:**6**(132)		**D21G**:**1**(21), **R61K**:**1**(3), **F48S**:**1**(14)	**N20S:1**(13), **R18K:1**(26), **T239A:1**(11)
**2019**	**A/Switzerland/8060/2017**: E5/E2, Clade 3C.2a2 (SH)	14 *(2017)*	8; 0.93	99.28 *(97.72-100)*				**V27A**$^{SM+}$:**1**(1), **G16E** $^{S+}$:**6**(24)	**G16E**:**6**(24)		**D21G**:**1**(7), **R61K**:**1**(1), **F48S**:**1**(4)	**N20S:1**(6), **R18K:1**(8), **T239A:1**(5)
	**A/Kansas/14/2017**: E5, Clade 3C.3a (NH)		10; 2.86	97.48 *(95.46-100)*				**I27A**:**1**(1), **I27V**:**13**(2,635), **G16E**$^{S+}$:**6**(24)	**G16E**:**6**(24)		**C52Y**:**14**(2610), **D21G**:**1**(7), **F48S**:**1**(4), **R61K**:**1**(1)	**N20S:1**(7), **R18K:1**(8), **T239A:1**(5)
**2020**	**A/South Australia/34/2019**: E5, Clade 3C.2a1b (SH)	14 *(2017)*	9; 1.93	98.34 *(96.59-100)*				**V27A**$^{SM+}$:**1***, **G16E**$^{S+}$:**6**(15)	**G16E**:**6**(15)		**D21G**:**1**(2), **F48S**:**1**(2), **R61K**:**1***, **L25P**:**14**(1393)	**N20S:1**(1), **R18K:1**(9), **T239A:1**(2)

Data are in n or n (%), unless otherwise indicated. AA= Amino acid. AAS= Amino acid substitutions. SH= Southern hemisphere vaccine. HN= Northern hemisphere vaccine. SNH= vaccine strain shared by both the Southern and Northern hemispheres for a given influenza season. Vaccine virus passage history indicated as indicated by FluServer. The total number of unique amino acid substitutions observed in all protein sequences and the mean number of substitutions per protein sequence are represented as N; n, where n = total number of amino acid substitutions divide by number of sequences analysed. The number of times a substitution is observed in the Uganda virus proteins and globally is reported in **bold** and (bracket) if available, respectively. Substitutions are colour-coded based on their known or predicted level of biological relevance. **Red** substitutions are the most significant (interestlevel =3) as they alter virulence, cause strong drug resistance and reverse premature stop codon in PB1-F2. The Orange (significant, interestlevel=2) occur at drug binding sites, affect host specificity and cause antigenic shift and mild drug resistance. Magenta (significant, interestlevel=2) adds or removes glycosylation sites. **Blue** substitutions (moderately significant, interestlevel=1) are involved at sites of interactions such as host cell receptor binding, binding small ligand(s), viral oligomerization interfaces, and antibody recognition sites. The **Green** substitutions (least significant, interestlevel=0) are common to subtypes while **Black** (least significant with warnlevel=0) have no known effects. Superscript symbol definitions: **"*"** = AAS reported for the first time (current global frequency = 0). "+" = AAS have their function reported in combination with others. **"R"** = AAS removes a potential glycosylation site. **"C"** = AAS creates a potential glycosylation site. **"S"** = AAS causes strong drug resistance. **"M"** = AAS causes mild drug resistance. "@" = AAS is a common subtype marker. Amino acid substitutions with multiple functions reported were indicated under all functions as appropriate. For substitutions observed for the first time (global frequency=0), their biological relevance (functions) are reported based on the functions of their equivalent positions in the resolved protein structures.

Supplementary Table 6: Amino acid substitutions in the complete protein sequences of HA (H3), NA (N2) and MP (M1 and M2) of Uganda A(H3N2) viruses compared to Southern and Northern Hemisphere vaccines. Amino acid similarity, amino acid substitutions and their corresponding functions and frequencies were obtained from Flusurver (http://flusurver.bii.a-star.edu.sg; accessed on 24th September 2021).

Supplementary Fig. 3: Multiple sequence alignment (MSA) of Uganda A(H3N2) M2 protein sequences with adamantine-susceptible A/New York/392/2004(H3N2)

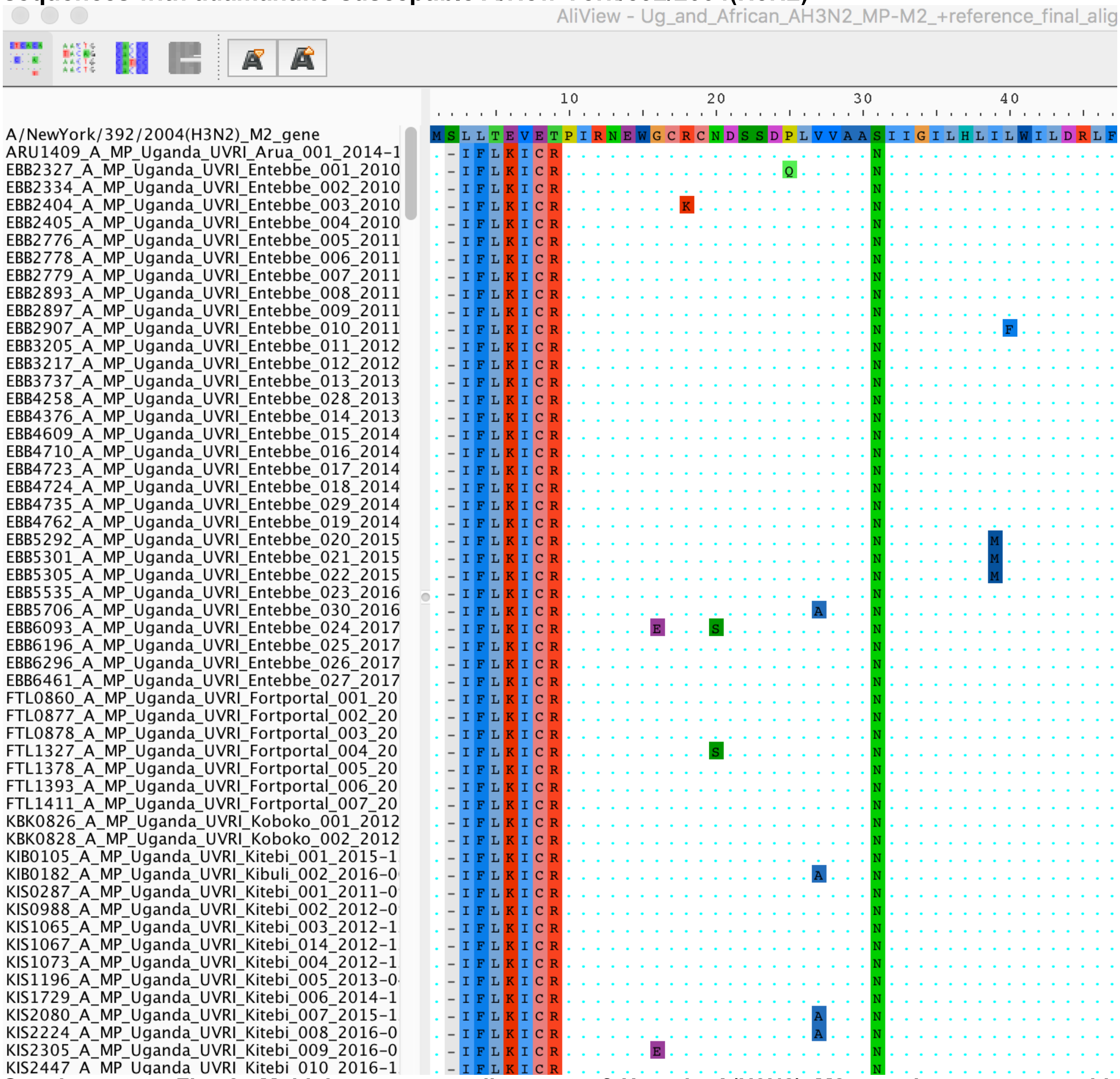

Supplementary Fig. 3: Multiple sequence alignment of Uganda A(H3N2) M2 protein sequences with adamantine-susceptible A/New York/392/2004(H3N2) as a reference. Substitutions with Alanine (A) and Asparagine (N) are observed in Uganda viruses at positions 27 and 31, respectively. Substitutions were visualized using AliView[1].

Supplementary Fig. 4: Phylogenies showing the spatial divergence Uganda influenza A viruses

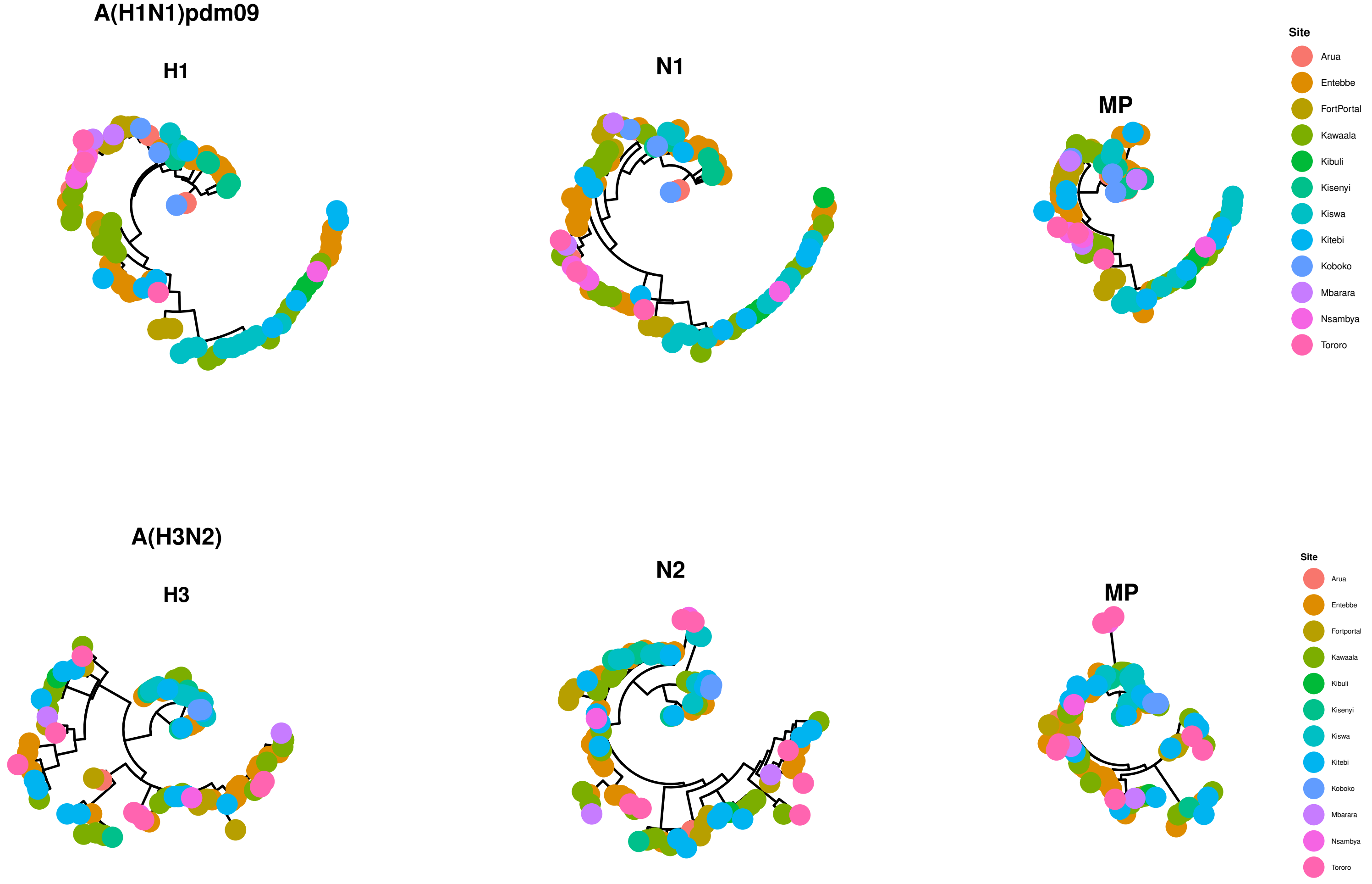

Supplementary Fig. 4: Phylogenies showing the spatial divergence of the HA, NA and MP genes of Uganda A(H1N1)pdm09 (A) and A(H3N2) (B) influenza viruses collected from 2010 to 2018. Trees were rooted using the oldest sequence in the Ugandan dataset. There was no uniqueness in viruses circulating in a given site or geographical region in all gene trees.

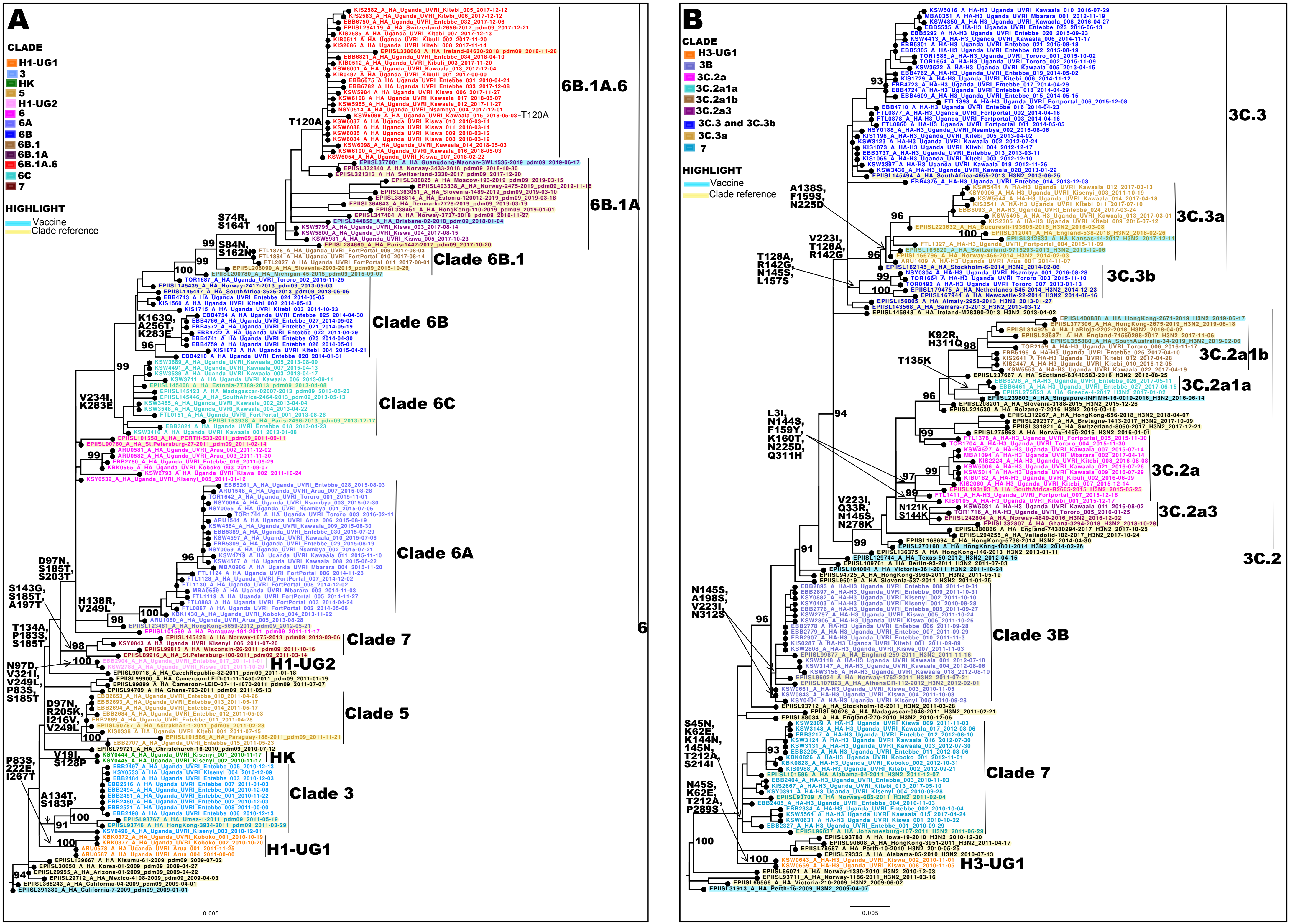
A
CLADE
H1-UG1
3
HK
5
H1-UG2
6
6A
6B
6B.1
6B.1A
6B.1A.6
6C
7
HIGHLIGHT
Vaccine
Clade reference
6B.1A.6
6B.1A
T120A
S74R, S164T
S84N, S162N
Clade 6B.1
Clade 6B
K163Q, A256T, K283E
Clade 6C
V234I, K283E
Clade 6A
D97N, S185T, S203T
S143G, S185T, A197T
H138R, V249L
T134A, P183S, S185T
Clade 7
H1-UG2
N97D, V321I, V249L, P83S, S185T
D97N, R205K, I216V, V249L
Clade 5
V19I, S128P
HK
P83S, 222E, I267T
A134T, S183P
Clade 3
H1-UG1
6
0.005
B
CLADE
H3-UG1
3B
3C.2a
3C.2a1a
3C.2a1b
3C.2a3
3C.3 and 3C.3b
3C.3a
7
HIGHLIGHT
Vaccine
Clade reference
3C.3
A138S, F159S, N225D
3C.3a
V223I, T128A, R142G
T128A, R142G, N145S, L157S
3C.3b
K92R, H311Q
T135K
3C.2a1b
3C.2a1a
L3I, N144S, F159Y, K160T, N225D, Q311H
3C.2a
N121K, S144K
3C.2a3
V223I, Q33R, N145S, N278K
3C.2
N145S, A198S, V223I, N312S
Clade 3B
S45N, K62E, K144N, 145N, T212A, S214I
Clade 7
N45S, K62E, T212A, P289S
H3-UG1
0.005

Supplementary Fig. 5: Genetic clades of influenza A viruses that previously circulated in 2010-2018 in Uganda. All labelled genetic clades (indicated by black bars) were inferred based on the signature amino acid substitutions in the HA1 protein (ECDC method), and for each clade these substitutions are indicated on the tree trunk in bold. **Panel A** shows genetic clades for 2010-2018 A(H1N1)pdm09 viruses. Novel clades H1-UG1 and H1-UG2 are indicated. Genetic clade 6 diverged into 6A, 6B, and 6C. All clade 3, 5, and 7 viruses were collected from Entebbe and Kampala (Central Uganda) and circulated in 2010-2011. **Panel B** shows genetic clades for 2010-2017 A(H3N2) viruses. A novel clade H3-UG1 characterized with 183H, 212A, 214I, and 289S is indicated. Clade 3 persisted in all the nine years while clade 7 re-emerged in 2017

Supplementary Table 7: Details of influenza A viral genetic clades that circulated in Africa from 1994 to 2019

Count	Genetic Clade	Signature substitutions in the HA1 protein	Origin of viruses in the clade	Geographical region	Duration (years) clade viruses circulated	Genetic Clade	Signature substitutions in the HA1 protein	Origin of viruses in the clade	Geographical region	Duration (years) clade viruses circulated
		**A(H1N1)pdm09**				**A(H3N2)**				
1.	**3**	A134T, S183P	Uganda	Eastern	2010-2011	**2**	N133D, R142G	South Africa	Southern	1994
2.	**5**	D97N, R205K, I216V, V249L	Kenya, Uganda	Eastern	2011	**3B**	N145S, A198S, V223I, N312S, D158N	Kenya, Uganda	Eastern	2010-2012
3.	**6**	D97N, S185T	Kenya, Uganda	Eastern	2010-2011	**3C**	T48I, N145S, V186G, A198S, V223I, N312S, D158N, N278K, Q33R, S45N	Uganda	Eastern	2012
4.	**6A**	H138R, V249L	Uganda	Eastern	2013-2016	**3C.2a**	L3I, N145S, F159Y, Q311H, V186G, K160T, N225D, Q311H	Kenya, Tanzania, Nigeria, South Africa, Mali, Burkina Faso, Ivory Coast, Ethiopia, Zambia, Uganda, Madagascar, Rwanda, Congo, Reunion,	Eastern, Western, Central, Southern	2014-2017
5.	**6B**	K163Q, A256T, K283E	Congo, Egypt, Ethiopia, Madagascar, Mali, Mozambique, Nigeria, Rwanda, South Africa, Tanzania, Kenya, Uganda, Zambia	Eastern, Western, Central, Northern, Southern	2014-2018	**3C.2a1**	N171K, N121K, Q311H	Congo, Mali, South Africa, Madagascar, Tanzania, Rwanda, Ethiopia, Reunion, Mayotte, Kenya	Eastern, Western, Central, Southern	2014-2016
6.	**6B.1**	S84N, S162N, I216T	Ethiopia, south Africa, Madagascar, Mali, Rwanda, Tanzania, Congo, Burkina Faso, Mozambique, Togo, Ivory Coast, Uganda	Eastern, Western, Central, Southern	2015-2018	**3c.2a1a**	T135K, Q311H	Uganda, South Africa, Togo, Niger, Nigeria Sierra Leone, Congo, Burkina Faso, Mali, Ivory Coast, Kenya	Eastern, Western, Central, Southern	2017-2019
7.	**6B.1A**	S74R, S164T	South Africa, Kenya, Togo, Tanzania, Niger, Congo, Nigeria, Burkina Faso, Uganda	Eastern, Western, Central, Southern	2017-2019	**3C.2a1b**	K92R, E62G, R142G, H311Q	Uganda, Tanzania, Madagascar, South Africa, Kenya, Mozambique, Niger, Nigeria, Mali, Burkina Faso, Togo, Ivory Coast,	Eastern, Western, Southern	2016-2019
8.	**6B.1A.1**	S183P, S74R, and S164T (majority had additional R45G, P282A, I298V)	Mozambique, South Africa, Uganda	Eastern, Southern	2018	**3C.2a2**	T131K, R142K, R261Q	Nigeria, Tanzania, South Africa	Eastern, Western, Southern	2017-2019
9.	**6B.1A.3**	T120A, P137S, A141E, H273Y	Kenya, Tanzania	Eastern	2018	**3C.2a3**	N121K, S144K	Ivory Coast, South Africa, Tanzania, Madagascar, Ethiopia, Togo, Kenya, Uganda	Eastern, Western, Southern	2015-2017
10.	**6B.1A.5a**	S183P, N260D, N129D	Madagascar, Tanzania, Congo, Burkina Faso	Eastern, Western, Central	2019	**3C.3**	T128A, R142G, N145S, S45N, T48I, A198S, V223I & N312S, Q33R	Uganda, Kenya, Congo	Eastern, Central	2012-2016
11.	**6B.1A.5b**	S183P, N260D, E235D	Ivory Coast, Mali, Burkina Faso, Sierra Leone, Niger, Nigeria,	Western	2017-2018	**3C.3a**	A138S, F159S, N225D (some with S91N, N144K, F193S)	Uganda, Ethiopia, Tanzania, Nigeria, Madagascar, Kenya, Togo	Eastern, Western	2011, 2014-2019
12	**6B.1A.6**	S74R, T120A (some have T2K, A315V), these are closest to clade 6B.1A.5	Uganda, Kenya, Madagascar, Nigeria, South Africa,	Eastern, Western, Southern	2017-2018	**3C.3b**	T128A, R142G, N145S, L157S, S45N, T48I, A198S, V223I, N312S, Q33R	Kenya, Uganda	Eastern	2013-2016
13	**6B.1A.6 (-T120A)**	S74R, S164T (Some have S183P**)**: viral sequences clustered within the 6B.1A.6 but lacked T120A	Tanzania, Congo, Uganda	Eastern, Central	2017-2018	**7**	S45N, K62E, K144N, 145N, 186G, T212A, S214I, 183H, 212A, 289P	Kenya, Uganda,	Eastern	2010-2012, 2017
14	**6B.2**	V152T, V173I	Burkina Faso, South Africa	Western, Southern	2015-2016	**H3-UG1**	N45S, K62E, K144N, 145N, 186G, T212A, S214I, L183H, S289P (virses from Uganda had P289S)	Kenya, Senegal, Uganda	Eastern, Western	2008-2010

15	**6C**	V234I, K283E	Uganda	Eastern	2013, 2015						
16	**7**	S143G, S185T, A197T	Uganda	Eastern	2011						
17	**H1-UG1**	D222E, P83S (those from Uganda had I267T)	Egypt, Tanzania, Uganda, Kenya	Eastern, Northern	2009-2011						
18	**H1-UG2**	N97D, V321I, V249L, P83S, S185T	Uganda	Eastern	2011						
19	**A/Hong Kong/2212/2010 (H1N1) clade (HK)**	V19I, N97D, S128P, N228S, I286V, I324V	Uganda	Eastern	2010						

Supplementary Table 7: Details of influenza A viral genetic clades that circulated in Africa from 1994 to 2019. Africa viral sequences were classified into globally defined genetic clades based on signature amino acid substitutions in their HA1 proteins[2]. Table summarises clade results in Fig. 3 and Supplementary Fig. 5.

Supplementary Fig. 6: Phylogenetic clustering and clades of Uganda and other Africa A(H1N1)pdm09 viruses in the hemagglutinin gene

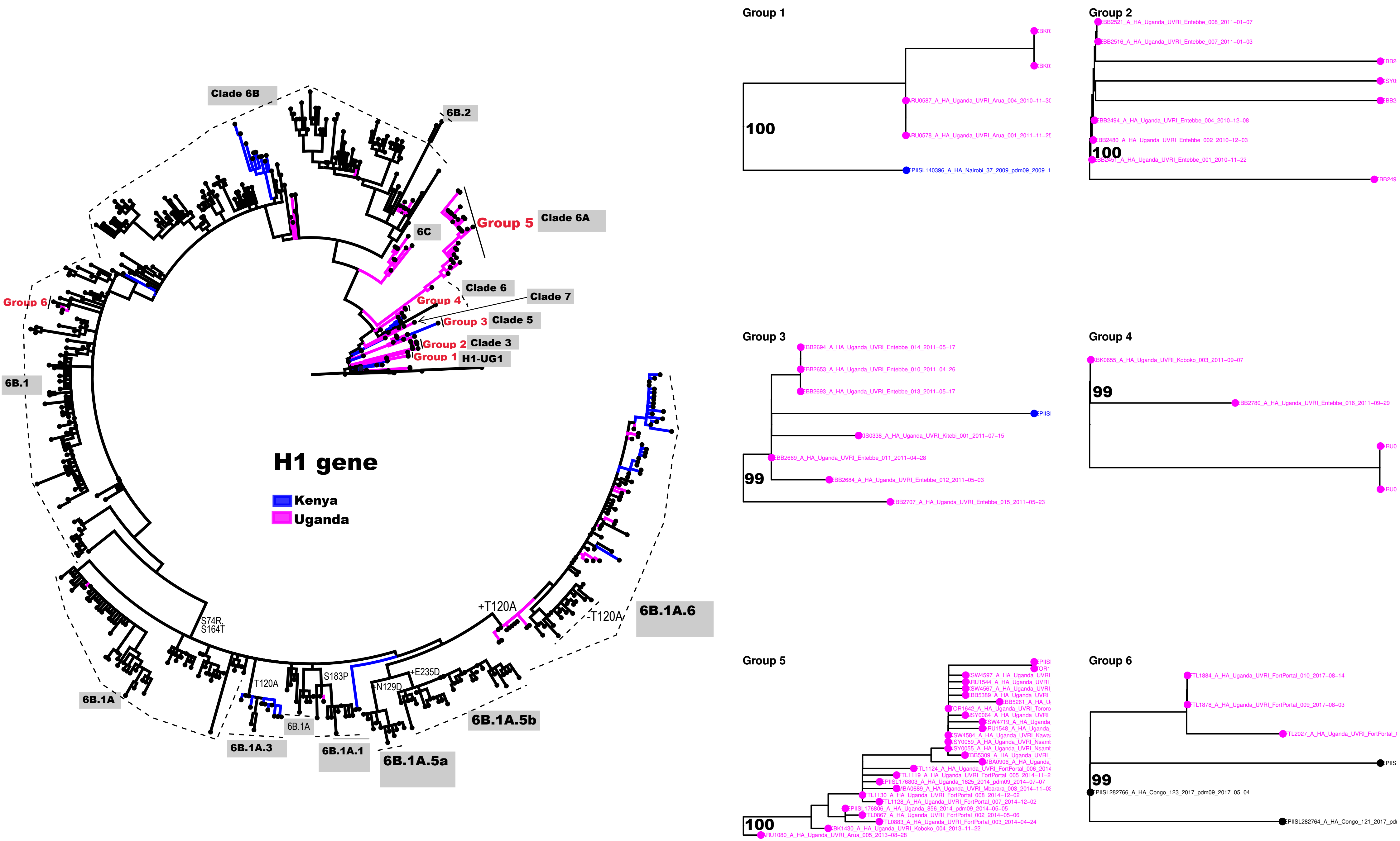

Supplementary Fig. 6: Phylogenetic clustering and clades of 2009-2018 Uganda A(H1N1)pdm09 viruses with other African viruses (2009-2019) in the HA (H1) gene. Groups are defined as highly supported phylogenetic clusters (bootstrap vlue equal or greater than 90%) with 3 or more Uganda virus sequences. All 6 groups are shown. Group details and bootstrap support are described in Supplementary Table 8 below. H1-UG2 not shown but was close to clade 7 with only 2 (sequences EBB2904 and KSW2788).

Supplementary Fig. 7: Phylogenetic clustering of Uganda and other Africa A(H1N1)pdm09 viruses in the neuraminidase gene

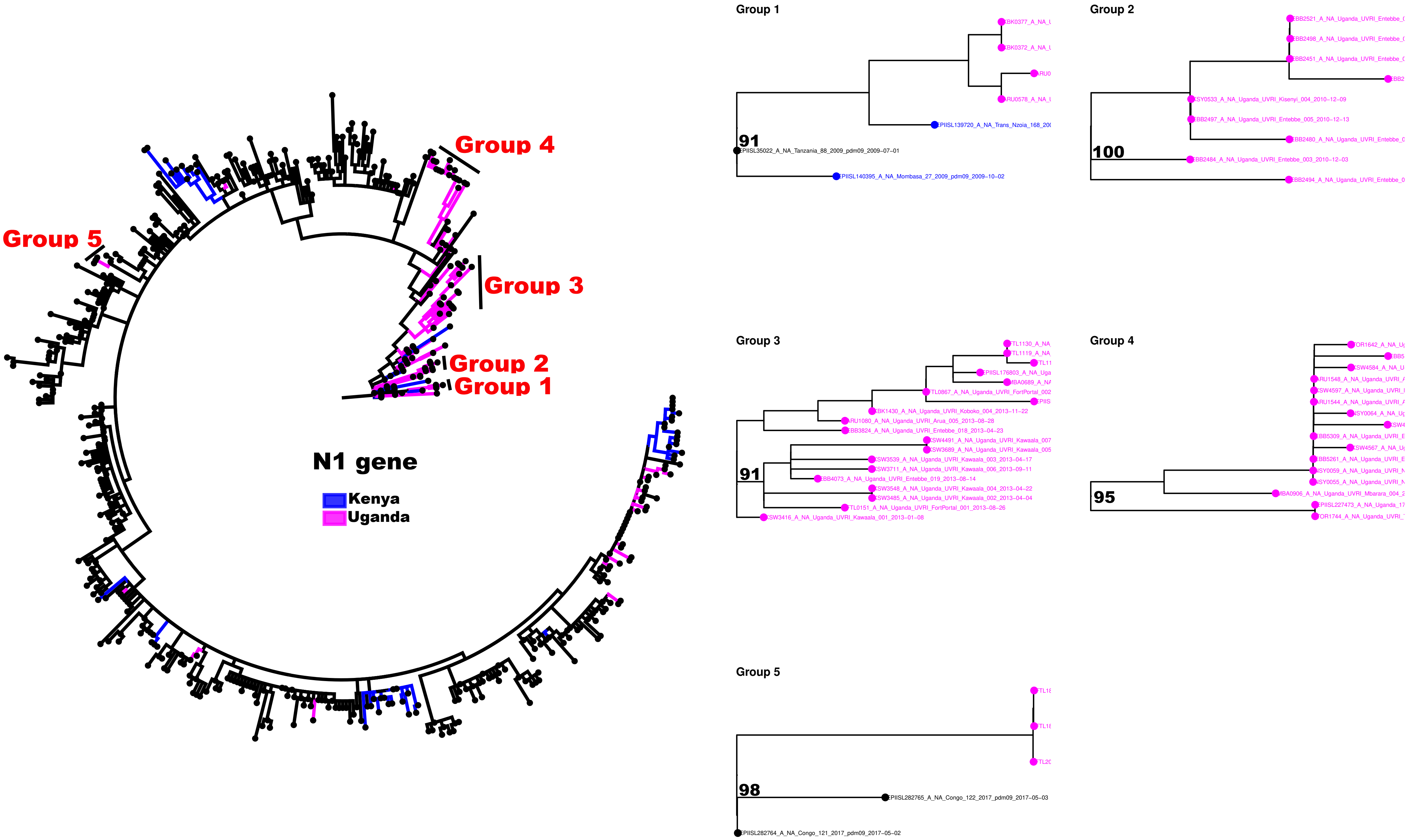

Supplementary Fig. 7: Phylogenetic clustering of 2009-2018 Uganda A(H1N1)pdm09 viruses with other African viruses (2009-2019) in the NA (N1) gene. All 5 groups are shown. Group details support are described in Supplementary Table 8 below.

Supplementary Fig. 8: Phylogenetic clustering of Uganda and other Africa A(H1N1)pdm09 viruses in the matrix protein gene

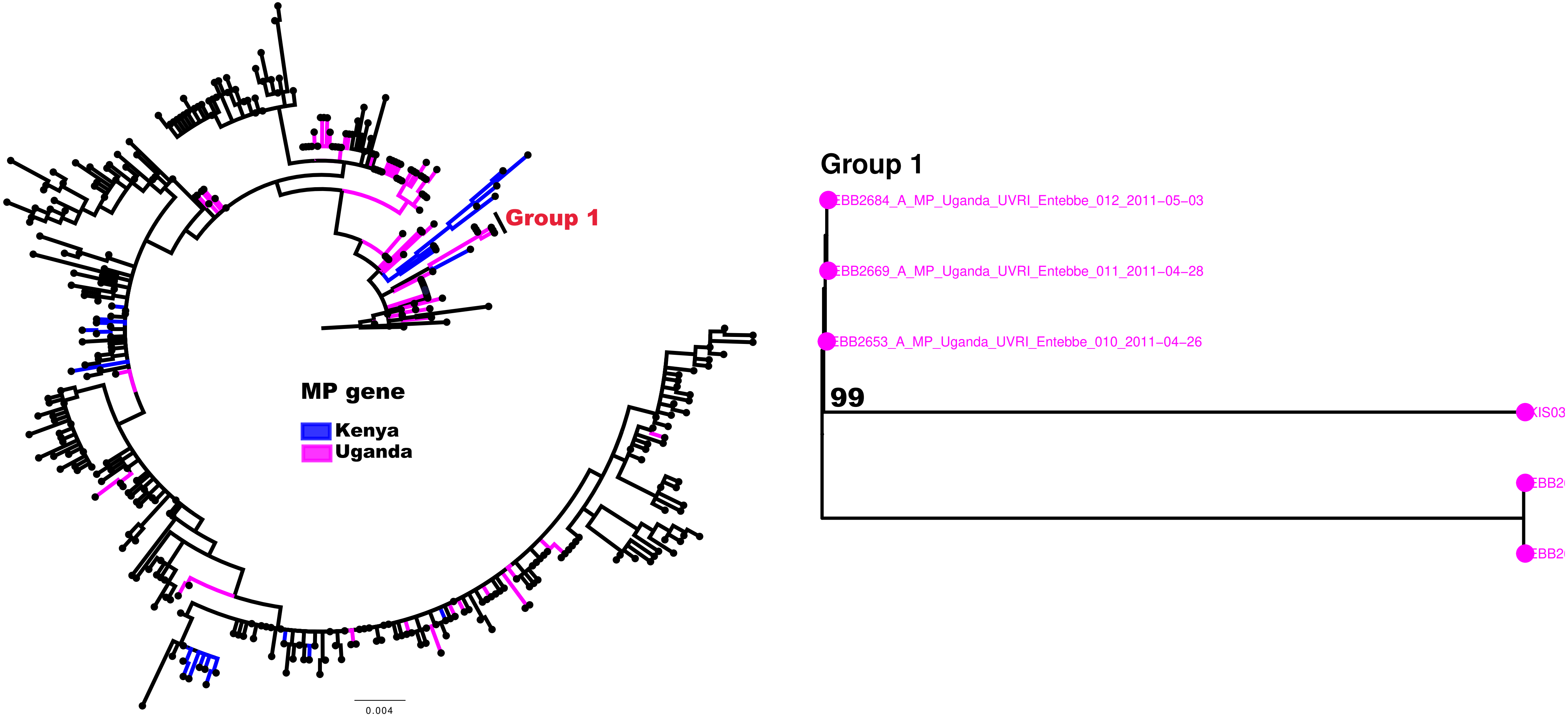

Supplementary Fig. 8: Phylogenetic clustering of 2009-2018 Uganda A(H1N1)pdm09 viruses with other African viruses (2009-2019) in the MP gene. Group details support are described in Supplementary Table 8 below.

Supplementary Fig. 9: Phylogenetic clustering and clades of Uganda and other Africa A(H3N2) viruses in the hemagglutinin gene

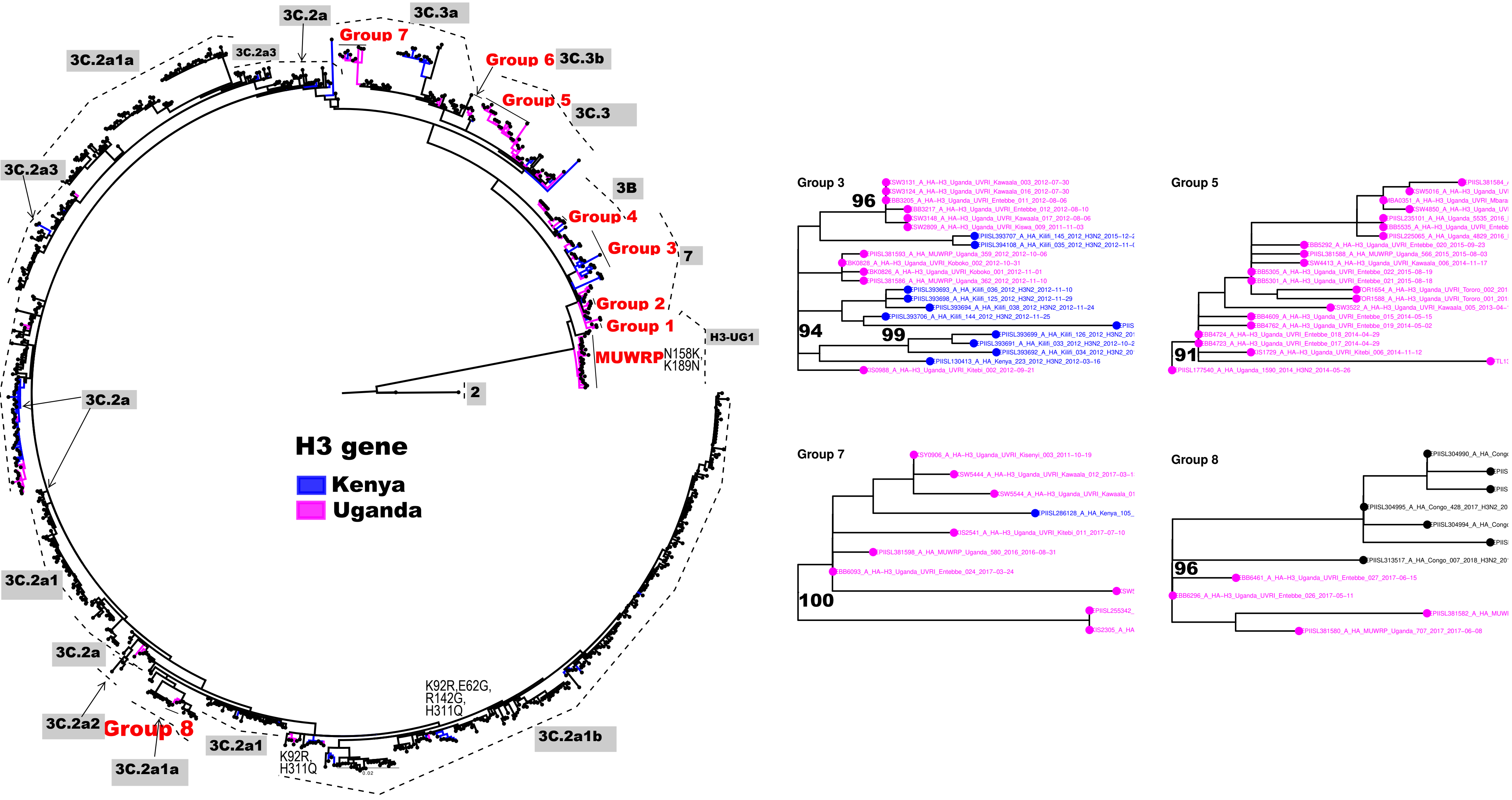

Supplementary Fig. 9: Phylogenetic clustering and clades of 2008- 2017 Uganda A(H3N2) viruses with other African viruses (1994-2019) in the HA (H3) gene. There were 8 groups. Only the 4 large groups are shown. The MWRP group contained viruses sampled in 2008 and had a bootstrap value 50%. Group details support are described in Supplementary Table 8 below.

Supplementary Fig. 10: Phylogenetic clustering of Uganda and other Africa A(H3N2) viruses in the neuraminidase gene

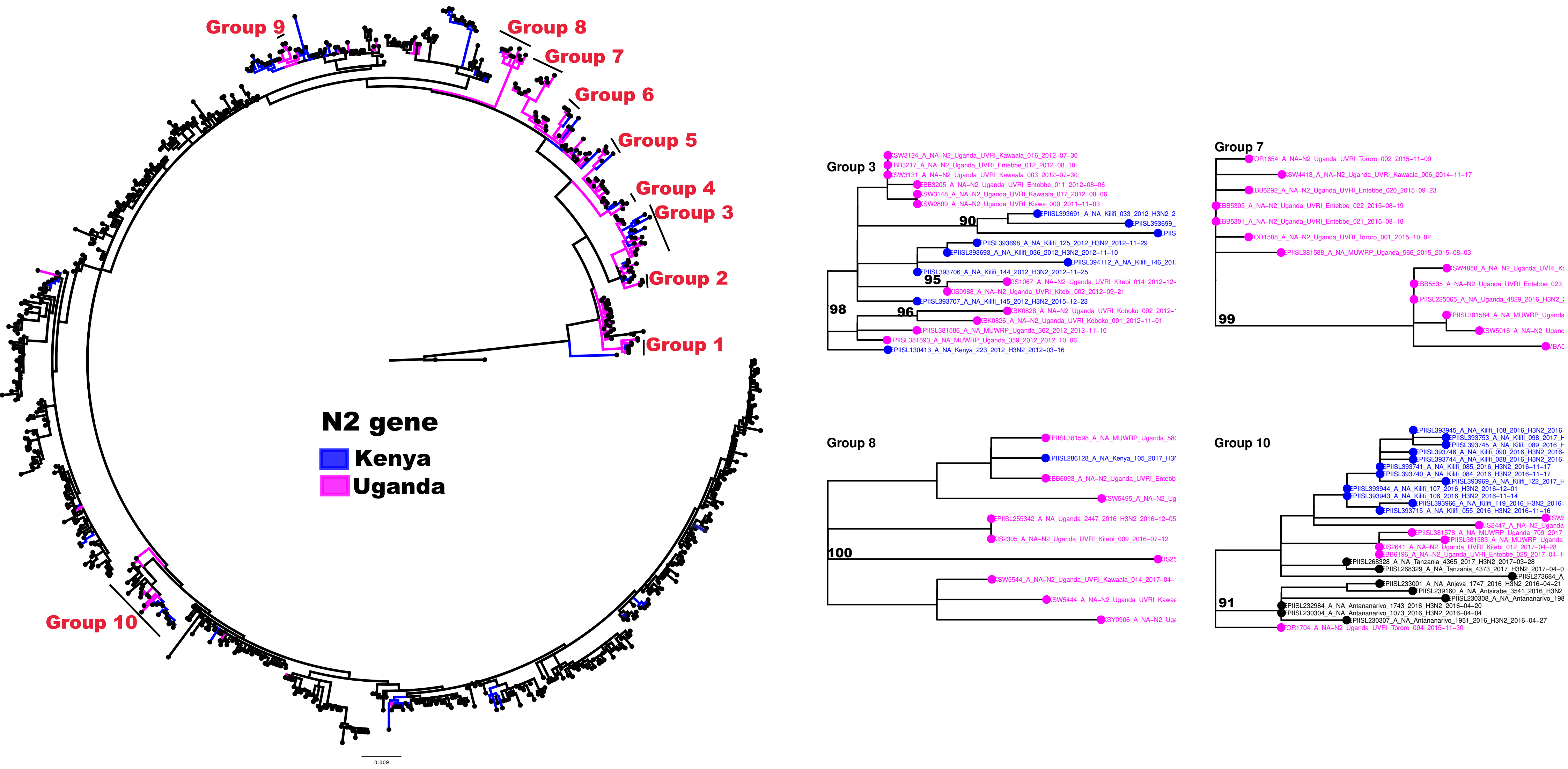

Supplementary Fig. 10: Phylogenetic clustering of 2008-2017 Uganda A(H3N2) viruses with other African viruses (1994-2019) in the NA (N2) gene. Only the large groups (4/10) with 10 or more Uganda sequences are shown. Group details support are described in Supplementary Table 8 below.

Supplementary Fig. 11: Phylogenetic clustering of Uganda and other Africa A(H3N2) viruses in the matrix protein gene

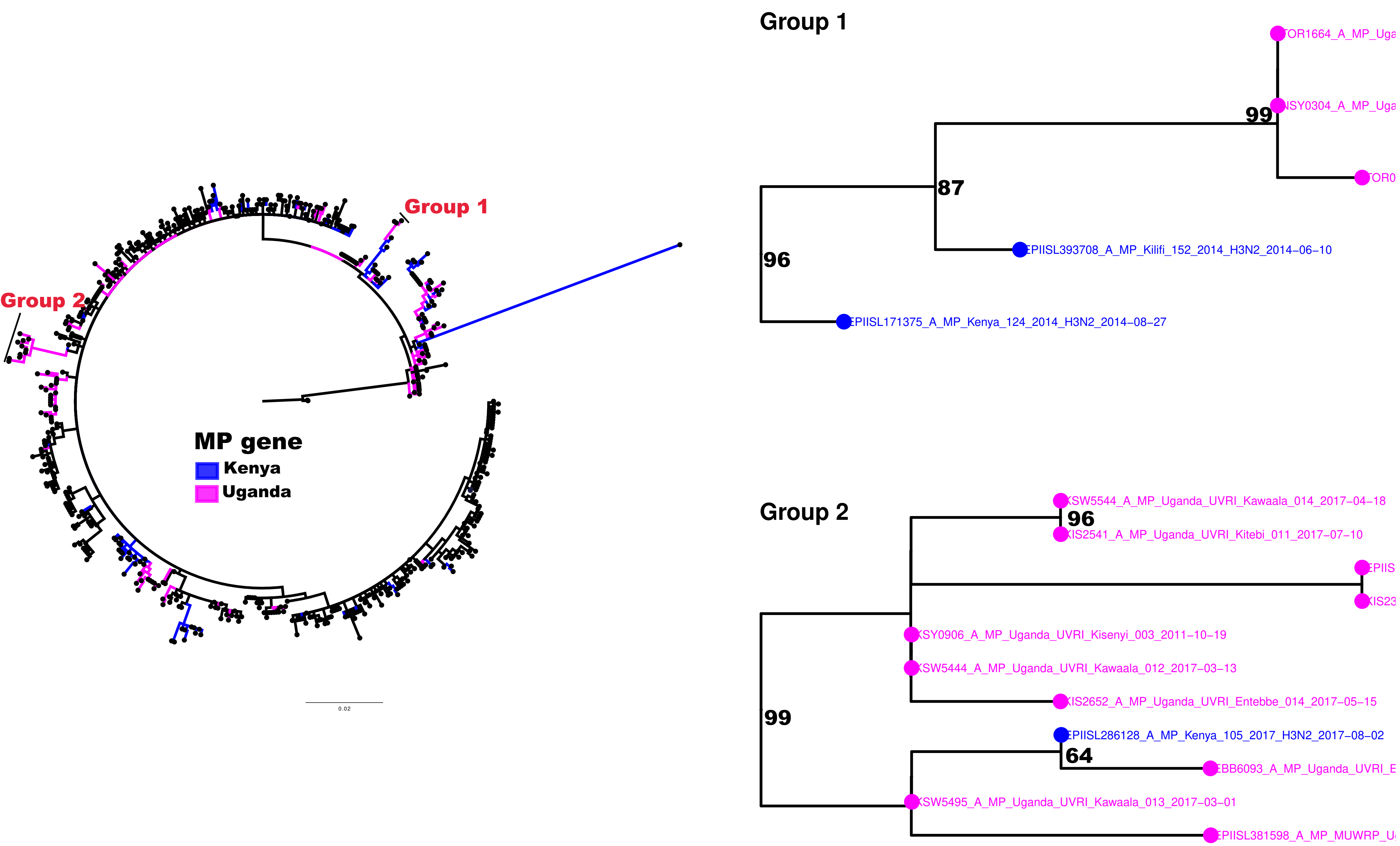

Supplementary Fig. 11: Phylogenetic clustering of 2008-2017 Uganda A(H3N2) viruses with other African viruses (1994-2019) in the MP gene. Only the large groups (9/15) with 5 or more Uganda sequences are displayed. Group details support are described in Supplementary Table 8 below.

Supplementary Table 8: Details of highly supported phylogenetic groups among the newly-generated Uganda viruses and other African viruses

Group	Bootstrap	Number of Uganda viruses/ total number of viruses in the group	Origin of other viruses in the group	Years group circulated	Clade to which group belongs
**A(H1N1)pdm09**					
**A. Hemagglutinin (H1)**					
**1**	100	4/5	Nairobi	2009 - 2011	**H1-UG1**
**2**	100	9/9	-	2010 -2011	**Clade 3**
**3**	99	7/8	Kenya	2011	**Clade 5**
**4**	99	4/4	-	2011	**Clade 6**
**5**	100	27/27	-	2013 -2016	**Clade 6A**
**6**	99	3/6	Congo	2017	**6B.1**
**Neuraminidase (N1)**					
**1**	91	3/7	Tanzania, Kenya	2009 - 2011	
**2**	100	9/9	-	2010 - 2011	
**3**	91	19/19	-	2013 - 2015	
**4**	95	16/16	-	2015-2016	
**5**	98	3/5	Congo	2017	
**Matrix protein (MP)**					
**1**	99	6/6	-	2011	

Group	Bootstrap	Number of Uganda viruses/ total number of viruses in the group	Origin of other viruses in the group	Years group circulated	Clade to which group belongs
**A(H3N2)**					
**B. Hemagglutinin (H3)**					
**1**	92	3/3	-	2009	**H3-UG1**
**2**	95	3/3	-	2010, 2017	**7**
**3**	94	11/22	Kilifi, Kenya	2011 – 2012	**7**
**4**	99	3/3	-	2011	**3B**
**5**	91	22/ 22	-	2014- 2016	**3C.3**
**6**	97	3/5	Kilifi, Kenya	2013 - 2016	**3C.3b**
**7**	100	9/10	Kenya	2011, 2016 - 2017	**3C.3a**
**8**	96	4/11	Congo	2017 - 2018	**3C.2a1a**
**Neuraminidase (N2)**					
**1**	99	5/8	Kilifi	2008 - 2010	
**2**	100	3/3	-	2010, 2017	
**3**	98	12/21	Kilifi	2011 - 2012	
**4**	98	3/3	-	2010 - 2011	
**5**	94	3/5	Kilifi, Kenya	2013 - 2016	
**6**	97	4/5	Kenya	2014	
**7**	99	13/13	-	2012, 2014-2016	
**8**	100	9/10	Kenya	2011, 2016 - 2017	
**9**	95	3/3	-	2015 - 2017	
**10**	91	7/28	Anjeva, Antsirabe, Antananarivo, Kilifi, Tanzania	2015 - 2017	
**Matrix protein (MP)**					
**1**	96	3/5	Kilifi, Kenya	2013 - 2016	
**2**	99	10/11	Kenya	2016 - 2017	

Supplementary Table 8: Highly supported phylogenetic groups (with bootstrap value ≥90% and at least 3 Uganda sequences) of Uganda A(H1N1)pdm09 and A(H3N2) viruses isolated in 2008-2018 [including (2008-2009) Makerere Walter Reed project (MWRP) and our new (2010-2018) dataset] with other African viruses (1994-2019) in the HA, NA, and MP genes. The number of Uganda viruses, country or city of origin for other African viruses, and years of viral sampling are reported for each group per gene. **Panel A** shows groups observed in the A(H1N1)pdm09 phylogenies. Panel B shows groups observed in the A(H3n2) phylogenies. The A(H3N2) group 1 consisted of the only MWRP viruses sampled in 2009. This table summarises information on the phylogenetic trees in Supplementary Fig. 6-11.

Supplementary materials and methods

Source of swabs and sampling

Nasal and oropharyngeal swabs were collected from outpatients and inpatients with influenza-like illnesses (ILI) and severe acute respiratory illnesses (SARI) at the different sentinel sites, respectively, as described by Lutwama *et.al*[3]. The swabs were tested for influenza (A and B) and the IAV were further subtyped for seasonal [A(H1N1) and A(H3N2)] and pandemic A(H1N1)pdm09 influenza using the Centers for Disease Control's (CDC) real-time reverse-transcription polymerase chain reaction (rRT-PCR) protocols and primers (Atlanta, Georgia)[4] . All patient swabs were uniquely coded and frozen at -80^0C, and their sociodemographic data recorded using EpiInfo (CDC, Atlanta)[3].

Viral RNA isolation and amplification

Following isolation, the viral RNA was reverse transcribed into cDNA and the entire IAV genome amplified using the multi-segment real-time polymerase chain reaction (M-RTPCR)[5] and universal IAV Uni/Inf primers in 25 µL reactions containing 8 µL nuclease-free water, 12.5 µL 2× RT-PCR buffer, 0.2 µL Uni12/Inf1 (10 µM), 0.3 µL Uni12/Inf3 (10 µM), 0.5 µL Uni13/Inf1 (10 µM), 0.5 µL SuperScript III One-Step RT-PCR with Platinum *Taq* High Fidelity (Invitrogen) and 3 µL extracted RNA. The M-RTPCR standardised thermocycling conditions were as follows: 42 °C for 50 minutes, 50 °C for 10 minutes, 94 °C for 2 minutes; 4 cycles (94 °C for 30 seconds, 43 °C for 30 seconds and 68 °C for 3 minutes and 50 seconds) followed by 30 cycles of 94 °C for 30 seconds, 57 °C for 30 seconds and 68 °C for 3 minutes and 30 seconds (with the 3 minutes and 30 seconds for the 68 °C extension step increased by 10 seconds per subsequent cycle after cycle 1); and a final extension step at 68 °C for 10 minutes.

Next-generation sequencing

Following PCR, the amplicons were purified using 1X AMPure XP beads (Beckman Coulter Inc., Brea, CA, USA), quantified with Quant-iT dsDNA High Sensitivity Assay (Invitrogen, Carlsbad, CA, USA), and normalized to 0.2 ng/µL. Indexed paired end libraries were then generated from 2.5 µL of 0.2 ng/µL amplicon pool using Nextera XT Sample Preparation Kit (Illumina, San Diego, CA, USA) following the manufacturer's protocol. Amplified libraries were purified using 0.8X AMPure XP beads, quantitated with Quant-iT dsDNA High Sensitivity Assay (Invitrogen, Carlsbad, CA, USA), and evaluated for fragment size in the Agilent 2100 BioAnalyzer System using the Agilent High Sensitivity DNA Kit (Agilent Technologies, Santa Clara, CA, USA). Libraries were then diluted to 2nM in preparation for pooling and denaturation for running on the Illumina MiSeq (Illumina, San Diego, CA, USA). Pooled libraries were sodium hydroxide denatured, diluted to 12.5 pM and sequenced on the Illumina MiSeq using 2 x 250 bp paired end reads with the MiSeq v2 500 cycle kit (Illumina, San Diego, CA, USA). Five percent Phi-X (Illumina, San Diego, CA, USA) spike-in was added to the libraries to increase library diversity by creating a more diverse set of library clusters. The MiSeq generated paired reads as fastq.gz files for each sample.

Sequence quality control

Raw MiSeq reads were de-duplicated using FastUniq v1.1[6] and Trimmomatic v0.39[7] was used to trim off Nextera transposase, adaptors, and PCR primers from the unique reads, retaining only reads with ≥80 bps. Clean reads were used as input for the Iterative Refinement Meta-Assembler (IRMA) assembly[8].

Genome assembly using Iterative Refinement Meta-Assembler (IRMA)

The sequence reads were assembled using a reference-based pre-built module (FLU) implemented in the Iterative Refinement Meta-Assembler's (IRMA)[8].

Due to the rapid evolution of influenza viruses, finding a single reference sequence with optimal coverage remains a challenge that IRMA tries to solve. IRMA uses a set of sequences or references which it iteratively refines to find an optimal consensus reference sequence for an optimal assembly. Specifically, IRMA's IAV reference set was built using publicly available genetic data (GenBank) for all 8 gene segments for all viral subtypes. Ineligible sequences i.e., duplicates, with more than 5 ambiguous bases, causing frame-shifts, and shorter than 60 % alignment length were excluded. Eligible sequences were aligned using MAFFT v7 to generate a consensus sequence set which IRMA uses as a default reference set for the FLU module.

We assembled the IAV genomes using the IRMA default settings as follows: median read quality score (Q-score) filter of 30; minimum read length of 125; frequency threshold for insertion and deletion refinement of 0.25 and 0.6, respectively; mismatch penalty of 5; and gap opening penalty of 10^8. The IRMA output included: consensus sequences for all the eight gene segments, paired read counts, coverage depth, allele frequencies, and statistically supported variants for each sample.

Identification of amino acid substitutions and their biological relevance across the complete HA, NA, and MP proteins of Uganda IAVs using the influenza surveillance (FluSurver) webtool

FluSurver is an influenza surveillance tool (http://flusurver.bii.a-star.edu.sg) used to identify and highlight phenotypically and epidemiologically important candidate amino acid substitutions among human influenza viruses for further investigation.

FluSurver compares the input viral protein sequences (multiple sequence alignment) to vaccine protein sequences and identify candidate amino acids, their biological relevance based on existing curated literature, their global frequency relative all sequences submitted in GenBank, and their binding activities (ligands binding, host-cell binding, antibody-binding,

and viral oligomerization interfaces) by visualizing resolved protein structures in the Protein Data Bank. FluSurver assigns a colour to each candidate substitution based on the significance of its biological function, reported as an "interestlevel". Specifically, the most significant substitutions (interestlevel=3) are those that alter the viral virulence, cause strong drug resistance, or reverse the premature PB1-F2 stop codon of the A(H1N1)pdm09 subtype and are coloured **Red**. Substitutions at drug-binding sites or that alter host cell specificity have an interestlevel of 2 (significant) and are coloured **Orange**. Also, substitutions whose equivalent site is known to cause antigenic shifts and mild drug resistance are coloured **Orange** and assigned an interest level of 2 (significant). **Magenta** is assigned to significant substitutions that create or remove a glycosylation site with interestlevel=2. **Blue** substitutions are moderately significant (interestlevel=1) with structural functions at sites of interaction such as host cell receptor binding, binding small ligand(s), viral oligomerization interfaces, and antibody recognition sites. **Green** substitutions are common to a given subtype but their function and **Black** substitutions have no known biological function, and all are least significant (interestlevel=0).

In this study, we aligned complete protein sequences for the newly-generated HA, NA, and MP per subtype using MUSCLE[9] and compared them to the reference vaccine virus sequences in the FluSurver webtool (http://flusurver.bii.a-star.edu.sg; accessed on 24th September 2021) to identify amino acid substitutions and their respective predicted biological functions.

Supplementary Fig. 12: Geographical distribution of sampled sentinel sites in the general UVRI-NIC influenza surveillance programme

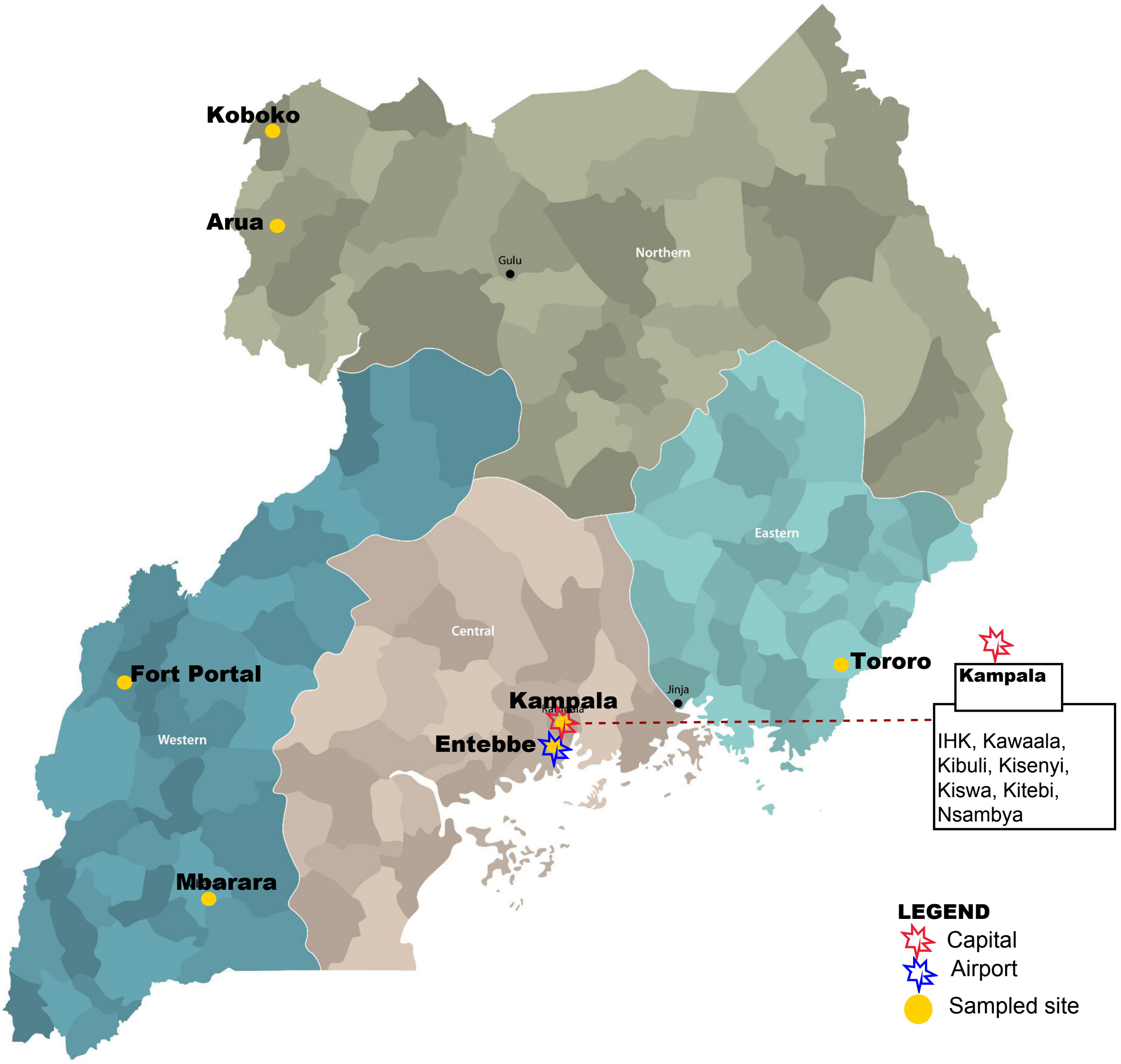

Supplementary Fig. 12: Geographical distribution of sampled sentinel sites in the UVRI-NIC influenza surveillance programme. District hospitals included Arua-ARU, Entebbe-EBB, Fort Portal-FTL, Koboko-KBK, Mbarara-MBA, Tororo-TOR, and Kampala. Kampala had three hospitals: International Hospital Kampala-IHK (International Hospital Kampala), Kibuli moslem hospital-KIB, Nsambya Hospital-NSY, and four clinics: Kawaala Health Center-KIS, Kiswa Health Center-KSW, Kisenyi Health Center-KSY, and Kitebi health centre-KIS samples. Swabs were assigned unique laboratory identification number using the site codes and a number. For example, EBB0001 for swab 0001 from Entebbe. The map template was purchased from https://www.alamy.com.

Supplementary Table 9: Accession numbers for the Africa influenza, clade references, and vaccine viral sequences analysed per subtype

	A(H1N1)pdm09	A(H3N2)
**Vaccine and clade reference viruses**	EPIISL391380, EPIISL200780, EPIISL344858, EPIISL377081, EPIISL368243, EPIISL90718, EPIISL29712, EPIISL139667, EPIISL93767, EPIISL93746, EPIISL29955, EPIISL79721, EPIISL30050, EPIISL90787, EPIISL101586, EPIISL90760, EPIISL101589, EPIISL101558, EPIISL89916], EPIISL99815, EPIISL145428, EPIISL94709, EPIISL99900, EPIISL99899, EPIISL123461, EPIISL145435, EPIISL145408, EPIISL145446, EPIISL145423, EPIISL145447, EPIISL153938, EPIISL200780, EPIISL206099, EPIISL284660, EPIISL294119, EPIISL344858, EPIISL364843, EPIISL338461, EPIISL347404, EPIISL332840, EPIISL321313, EPIISL40333, EPIISL363051, EPIISL388825, EPIISL388814	EPIISL31913, EPIISL104004, EPIISL129744, EPIISL165829, EPIISL270160, EPIISL239803, EPIISL331821, EPIISL312833, EPIISL355880, EPIISL400888, EPIISL66566, EPIISL93711, EPIISL86071, EPIISL93712, EPIISL96024, EPIISL96019, EPIISL90628, EPIISL79335, EPIISL78687, EPIISL93788, EPIISL90608, EPIISL93712, EPIISL99877, EPIISL107823, EPIISL94725, EPIISL101596, EPIISL93709, EPIISL96037, EPIISL107823, EPIISL109761, EPIISL145948, EPIISL136375, EPIISL143568, EPIISL156805, EPIISL145494, EPIISL168694], EPIISL193193, EPIISL223632, EPIISL162145, EPIISL312041, EPIISL166796, EPIISL167944, EPIISL179475, EPIISL208201, EPIISL224530, EPIISL237667, EPIISL275853, EPIISL286871, EPIISL314925, EPIISL275863, EPIISL312267, EPIISL292377, EPIISL242804, EPIISL332807, EPIISL294255, EPIISL286866, EPIISL377306
**Africa viruses**	"EPIISL106806" "EPIISL139708" "EPIISL139716" "EPIISL139719" "EPIISL139720" "EPIISL140396"<br>"EPIISL140404" "EPIISL140405" "EPIISL140406" "EPIISL140407" "EPIISL140408" "EPIISL140409"<br>"EPIISL140410" "EPIISL140411" "EPIISL140412" "EPIISL140413" "EPIISL171814" "EPIISL172640"<br>"EPIISL172641" "EPIISL172648" "EPIISL175254" "EPIISL175259" "EPIISL175880" "EPIISL176803"<br>"EPIISL176806" "EPIISL178482" "EPIISL178484" "EPIISL203293" "EPIISL203300" "EPIISL205286"<br>"EPIISL205287" "EPIISL205288" "EPIISL205293" "EPIISL205294" "EPIISL205297" "EPIISL205298"<br>"EPIISL205486" "EPIISL205487" "EPIISL206563" "EPIISL206565" "EPIISL206566" "EPIISL207384"<br>"EPIISL207387" "EPIISL207922" "EPIISL207923" "EPIISL207929" "EPIISL207930" "EPIISL207947"<br>"EPIISL207950" "EPIISL210330" "EPIISL210332" "EPIISL210340" "EPIISL211950" "EPIISL211951"<br>"EPIISL211952" "EPIISL213141" "EPIISL213145" "EPIISL213146" "EPIISL213147" "EPIISL213208"<br>"EPIISL214171" "EPIISL214908" "EPIISL214909" "EPIISL214910" "EPIISL214911" "EPIISL214912"<br>"EPIISL216223" "EPIISL216224" "EPIISL216226" "EPIISL216227" "EPIISL216256" "EPIISL216257"<br>"EPIISL216258" "EPIISL216261" "EPIISL216262" "EPIISL216284" "EPIISL216285" "EPIISL216286"<br>"EPIISL216287" "EPIISL218134" "EPIISL218135" "EPIISL218177" "EPIISL220958" "EPIISL220960"<br>"EPIISL220962" "EPIISL220965" "EPIISL220967"] "EPIISL220969" "EPIISL220999" "EPIISL221004"<br>"EPIISL221006" "EPIISL221008" "EPIISL221010" "EPIISL221654" "EPIISL223223" "EPIISL223224"<br>"EPIISL223226" "EPIISL223227" "EPIISL223229" "EPIISL223232" "EPIISL227473" "EPIISL230453"<br>"EPIISL230457" "EPIISL230459" "EPIISL230461" "EPIISL230465" "EPIISL230467" "EPIISL230470"<br>"EPIISL230471" "EPIISL230473" "EPIISL230529" "EPIISL230531" "EPIISL230532" "EPIISL230534"<br>"EPIISL230535" "EPIISL230536" "EPIISL230537" "EPIISL232134" "EPIISL232136" "EPIISL232587"<br>"EPIISL232588" "EPIISL232589" "EPIISL232590" "EPIISL232591" "EPIISL232592" "EPIISL232603"<br>"EPIISL232604" "EPIISL232605" "EPIISL232606" "EPIISL232607" "EPIISL232664" "EPIISL232666"<br>"EPIISL232667" "EPIISL232668" "EPIISL232669" "EPIISL232672" "EPIISL232674" "EPIISL232677"<br>"EPIISL232678" "EPIISL232679" "EPIISL232680" "EPIISL232683" "EPIISL232684" "EPIISL232686"<br>"EPIISL232688" "EPIISL233022" "EPIISL233023" "EPIISL233062" "EPIISL233063" "EPIISL233065"<br>"EPIISL233066" "EPIISL233489" "EPIISL233490" "EPIISL235013" "EPIISL235015" "EPIISL235016"<br>"EPIISL235017" "EPIISL235339" "EPIISL235340" "EPIISL235341" "EPIISL235342" "EPIISL235343"<br>"EPIISL235344" "EPIISL235345" "EPIISL235346" "EPIISL236216" "EPIISL236217" "EPIISL236837"<br>"EPIISL237808" "EPIISL237809" "EPIISL237811" "EPIISL237813" "EPIISL237822" "EPIISL240213"<br>"EPIISL248209" "EPIISL248210" "EPIISL248211" "EPIISL248212" "EPIISL248213" "EPIISL248216"<br>"EPIISL248217" "EPIISL256091" "EPIISL256092" "EPIISL268443" "EPIISL268444" "EPIISL268448"<br>"EPIISL268456" "EPIISL268462" "EPIISL273782" "EPIISL275663" "EPIISL275664" "EPIISL275665"<br>"EPIISL275666" "EPIISL275667" "EPIISL281577" "EPIISL281578" "EPIISL281579" "EPIISL281581"<br>"EPIISL281583" "EPIISL281585" "EPIISL281587" "EPIISL281588" "EPIISL281589" "EPIISL281590"<br>"EPIISL281613" "EPIISL281616" "EPIISL281618" "EPIISL281619" "EPIISL281620" "EPIISL281621"<br>"EPIISL281622" "EPIISL281623" "EPIISL281624" "EPIISL281625" "EPIISL281626" "EPIISL281629"<br>"EPIISL282764" "EPIISL282765" "EPIISL282766" "EPIISL282771" "EPIISL282776" "EPIISL283210"<br>"EPIISL283212" "EPIISL283213" "EPIISL283214" "EPIISL283215" "EPIISL283217" "EPIISL283218"<br>"EPIISL283219" "EPIISL283221" "EPIISL283222" "EPIISL283223" "EPIISL283225" "EPIISL283226"<br>"EPIISL283227" "EPIISL283229" "EPIISL283231" "EPIISL283233" "EPIISL283235" "EPIISL283237"<br>"EPIISL285866" "EPIISL287686" "EPIISL287687" "EPIISL287688" "EPIISL287704" "EPIISL287706"<br>"EPIISL287707" "EPIISL287708" "EPIISL288566" "EPIISL289562" "EPIISL292622" "EPIISL292623"<br>"EPIISL292624" "EPIISL292625" "EPIISL292626" "EPIISL292627" "EPIISL292628" "EPIISL292629"<br>"EPIISL292630" "EPIISL292631" "EPIISL296234" "EPIISL296235" "EPIISL296237" "EPIISL298512"<br>"EPIISL298513" "EPIISL298514" "EPIISL298515" "EPIISL298516" "EPIISL298517" "EPIISL298518"	"EPIISL130413" "EPIISL149681" "EPIISL171352" "EPIISL171359" "EPIISL171375" "EPIISL171376" "EPIISL171378" "EPIISL171380"<br>"EPIISL171381" "EPIISL171394" "EPIISL171399" "EPIISL172531" "EPIISL172532" "EPIISL172592" "EPIISL172737" "EPIISL172750"<br>"EPIISL172752" "EPIISL172755" "EPIISL172759" "EPIISL172773" "EPIISL172774" "EPIISL172776" "EPIISL172777" "EPIISL172779"<br>"EPIISL172781" "EPIISL172784" "EPIISL172785" "EPIISL172787" "EPIISL172788" "EPIISL172799" "EPIISL172800" "EPIISL172802"<br>"EPIISL172803" "EPIISL172808" "EPIISL172811" "EPIISL172814" "EPIISL174121" "EPIISL174127" "EPIISL174137" "EPIISL175197"<br>"EPIISL175202" "EPIISL175210" "EPIISL175211" "EPIISL175217" "EPIISL176513" "EPIISL176515" "EPIISL176547" "EPIISL176548"<br>"EPIISL176570" "EPIISL177536" "EPIISL177540" "EPIISL188871" "EPIISL189842" "EPIISL191672" "EPIISL191693" "EPIISL191694"<br>"EPIISL192157" "EPIISL192158" "EPIISL192190" "EPIISL202451" "EPIISL203567" "EPIISL203587" "EPIISL203588" "EPIISL205282"<br>"EPIISL205283" "EPIISL205284" "EPIISL206111" "EPIISL206166" "EPIISL206167" "EPIISL206171" "EPIISL206172" "EPIISL206173"<br>"EPIISL206175" "EPIISL206177" "EPIISL206178" "EPIISL206179" "EPIISL206180" "EPIISL206181" "EPIISL206183" "EPIISL206184"<br>"EPIISL206190" "EPIISL206191" "EPIISL206193" "EPIISL206194" "EPIISL206195" "EPIISL206196" "EPIISL206197" "EPIISL206199"<br>"EPIISL206203" "EPIISL206204" "EPIISL206206" "EPIISL206207" "EPIISL206208" "EPIISL206211" "EPIISL206213" "EPIISL206214"<br>"EPIISL206244" "EPIISL207025" "EPIISL207394" "EPIISL207395" "EPIISL207422" "EPIISL207431" "EPIISL207434" "EPIISL207455"<br>"EPIISL207457" "EPIISL211679" "EPIISL211681" "EPIISL211682" "EPIISL211683" "EPIISL211685" "EPIISL211686" "EPIISL211687"<br>"EPIISL211691" "EPIISL211693" "EPIISL211694" "EPIISL211705" "EPIISL211908" "EPIISL211910" "EPIISL211912" "EPIISL211919"<br>"EPIISL211931" "EPIISL212061" "EPIISL212366" "EPIISL212370" "EPIISL212371" "EPIISL212999" "EPIISL213001" "EPIISL213003"<br>"EPIISL213004" "EPIISL213005" "EPIISL213019" "EPIISL213975" "EPIISL213976" "EPIISL213977" "EPIISL213978" "EPIISL213979"<br>"EPIISL215614" "EPIISL215615" "EPIISL215616" "EPIISL215617" "EPIISL215618" "EPIISL215619" "EPIISL215628" "EPIISL215629"<br>"EPIISL215631" "EPIISL215632" "EPIISL215633" "EPIISL215635" "EPIISL215636" "EPIISL215770" "EPIISL215779" "EPIISL220268"<br>"EPIISL220272" "EPIISL220275" "EPIISL220283" "EPIISL224270" "EPIISL225057" "EPIISL225058" "EPIISL225065" "EPIISL226798"<br>"EPIISL230295" "EPIISL230296" "EPIISL230297" "EPIISL230298" "EPIISL230299" "EPIISL230300" "EPIISL230301" "EPIISL230302"<br>"EPIISL230303" "EPIISL230304" "EPIISL230305" "EPIISL230306" "EPIISL230307" "EPIISL230308" "EPIISL230364" "EPIISL230365"<br>"EPIISL230366" "EPIISL230368" "EPIISL230369" "EPIISL230372" "EPIISL230373" "EPIISL230375" "EPIISL232079" "EPIISL232093"<br>"EPIISL232094" "EPIISL232096" "EPIISL232098" "EPIISL232540" "EPIISL232541" "EPIISL232543" "EPIISL232546" "EPIISL232555"<br>"EPIISL232562" "EPIISL232564" "EPIISL233001" "EPIISL233726" "EPIISL235073" "EPIISL235075" "EPIISL235077" "EPIISL235088"<br>"EPIISL235090" "EPIISL235091" "EPIISL235092" "EPIISL235093" "EPIISL235101" "EPIISL235104" "EPIISL235363" "EPIISL235537"<br>"EPIISL249955" "EPIISL255341" "EPIISL255342" "EPIISL268306" "EPIISL268307" "EPIISL268308" "EPIISL268309" "EPIISL268310"<br>"EPIISL268311" "EPIISL268313" "EPIISL268315" "EPIISL268316" "EPIISL268317" "EPIISL268318" "EPIISL268319" "EPIISL268320"<br>"EPIISL268321" "EPIISL268322" "EPIISL268323" "EPIISL268324" "EPIISL268325" "EPIISL268326" "EPIISL268328" "EPIISL268331"<br>"EPIISL270211" "EPIISL270212" "EPIISL273663" "EPIISL273665" "EPIISL273667" "EPIISL273671" "EPIISL273684" "EPIISL273686"<br>"EPIISL275712" "EPIISL275928" "EPIISL275930" "EPIISL275932" "EPIISL275934" "EPIISL275936" "EPIISL275940" "EPIISL275943"<br>"EPIISL275946" "EPIISL277139" "EPIISL278059" "EPIISL279044" "EPIISL279209" "EPIISL279214" "EPIISL279218" "EPIISL279252"<br>"EPIISL279253" "EPIISL279254" "EPIISL281573" "EPIISL281685" "EPIISL281746" "EPIISL281747" "EPIISL281748" "EPIISL281749"<br>"EPIISL281753" "EPIISL281755" "EPIISL281756" "EPIISL281757" "EPIISL281760" "EPIISL281771" "EPIISL281782" "EPIISL281802"<br>"EPIISL281808" "EPIISL281817" "EPIISL281837" "EPIISL281848" "EPIISL281871" "EPIISL281877" "EPIISL281878" "EPIISL281882"<br>"EPIISL281885" "EPIISL281888" "EPIISL282861" "EPIISL283312" "EPIISL285892" "EPIISL286127" "EPIISL286128" "EPIISL287619"<br>"EPIISL288511" "EPIISL288512" "EPIISL288513" "EPIISL288515" "EPIISL288516" "EPIISL288517" "EPIISL288518" "EPIISL288519"<br>"EPIISL290658" "EPIISL290659" "EPIISL290660" "EPIISL290661" "EPIISL292535" "EPIISL292537" "EPIISL292538" "EPIISL292539"<br>"EPIISL296085" "EPIISL296523" "EPIISL299831" "EPIISL299834" "EPIISL299840" "EPIISL299842" "EPIISL299843" "EPIISL299852"<br>"EPIISL299853" "EPIISL300840" "EPIISL304989" "EPIISL304990" "EPIISL304993" "EPIISL304994" "EPIISL304995" "EPIISL304996"<br>"EPIISL306260" "EPIISL313517" "EPIISL319709" "EPIISL319711" "EPIISL320281" "EPIISL320344" "EPIISL320345" "EPIISL320346"<br>"EPIISL320347" "EPIISL320348" "EPIISL320349" "EPIISL320351" "EPIISL320356" "EPIISL320360" "EPIISL322930" "EPIISL322934"<br>"EPIISL322946" "EPIISL329853" "EPIISL330475" "EPIISL331067" "EPIISL331507" "EPIISL331761" "EPIISL331764" "EPIISL331766"<br>"EPIISL331767" "EPIISL335644" "EPIISL335645" "EPIISL335646" "EPIISL335647" "EPIISL335648" "EPIISL335649" "EPIISL335656"<br>"EPIISL336501" "EPIISL336503" "EPIISL336504" "EPIISL338561" "EPIISL338566" "EPIISL338619" "EPIISL338621" "EPIISL338622"

	"EPIISL298519" "EPIISL298520" "EPIISL298522" "EPIISL298524" "EPIISL298525" "EPIISL298526"<br>"EPIISL298528" "EPIISL298529" "EPIISL299835" "EPIISL299837" "EPIISL299838" "EPIISL299845"<br>"EPIISL299846" "EPIISL299849" "EPIISL299850" "EPIISL299851" "EPIISL300845" "EPIISL300849"<br>"EPIISL300851" "EPIISL300852" "EPIISL300853" "EPIISL300854" "EPIISL300855" "EPIISL300857"<br>"EPIISL300859" "EPIISL300885" "EPIISL300901" "EPIISL300903" "EPIISL300909" "EPIISL300913"<br>"EPIISL300916" "EPIISL305251" "EPIISL305252" "EPIISL305253" "EPIISL305254" "EPIISL305265"<br>"EPIISL305266" "EPIISL305267" "EPIISL305269" "EPIISL305270" "EPIISL305271" "EPIISL305275"<br>"EPIISL305277" "EPIISL305912" "EPIISL309495" "EPIISL309496" "EPIISL309497" "EPIISL309498"<br>"EPIISL312940" "EPIISL312953" "EPIISL312956" "EPIISL313594" "EPIISL313595" "EPIISL313596"<br>"EPIISL313597" "EPIISL316454" "EPIISL319781" "EPIISL319782" "EPIISL319783" "EPIISL319784"<br>"EPIISL319785" "EPIISL319786" "EPIISL319787" "EPIISL319788" "EPIISL319789" "EPIISL319792"<br>"EPIISL319793" "EPIISL319794" "EPIISL319795" "EPIISL319796" "EPIISL319799" "EPIISL319800"<br>"EPIISL319801" "EPIISL319802" "EPIISL319803" "EPIISL319828" "EPIISL319829" "EPIISL319830"<br>"EPIISL319831" "EPIISL319832" "EPIISL319833" "EPIISL319834" "EPIISL319835" "EPIISL319836"<br>"EPIISL319837" "EPIISL319839" "EPIISL320256" "EPIISL320257" "EPIISL320258" "EPIISL320259"<br>"EPIISL320260" "EPIISL320693" "EPIISL320710" "EPIISL320713" "EPIISL321969" "EPIISL322857"<br>"EPIISL322858" "EPIISL322860" "EPIISL322861" "EPIISL322862" "EPIISL322863" "EPIISL322867"<br>"EPIISL330360" "EPIISL330361" "EPIISL330362" "EPIISL330364" "EPIISL330366" "EPIISL330367"<br>"EPIISL330368" "EPIISL330370" "EPIISL330371" "EPIISL330372" "EPIISL330374" "EPIISL330375"<br>"EPIISL330377" "EPIISL330380" "EPIISL330381" "EPIISL330382" "EPIISL330383" "EPIISL330384"<br>"EPIISL330385" "EPIISL330386" "EPIISL330388" "EPIISL330389" "EPIISL330390" "EPIISL330391"<br>"EPIISL330392" "EPIISL330393" "EPIISL330394" "EPIISL330397" "EPIISL330823" "EPIISL330824"<br>"EPIISL330825" "EPIISL330830" "EPIISL330836" "EPIISL330842" "EPIISL330843" "EPIISL330844"<br>"EPIISL330847" "EPIISL330850" "EPIISL330851" "EPIISL330852" "EPIISL330853" "EPIISL330854"<br>"EPIISL330856" "EPIISL330857" "EPIISL330858" "EPIISL330861" "EPIISL330864" "EPIISL331563"<br>"EPIISL331564" "EPIISL331565" "EPIISL331566" "EPIISL331946" "EPIISL331951" "EPIISL331952"<br>"EPIISL331956" "EPIISL332315" "EPIISL332397" "EPIISL332398" "EPIISL33576" "EPIISL335852"<br>"EPIISL335854" "EPIISL335855" "EPIISL335858" "EPIISL335878" "EPIISL335879" "EPIISL335880"<br>"EPIISL335883" "EPIISL335884" "EPIISL335911" "EPIISL335951" "EPIISL335952" "EPIISL335954"<br>"EPIISL335955" "EPIISL336635" "EPIISL336636" "EPIISL336637" "EPIISL336638" "EPIISL336639"<br>"EPIISL338706" "EPIISL34539" "EPIISL349812" "EPIISL349814" "EPIISL35013" "EPIISL35021"<br>"EPIISL35022" "EPIISL351839" "EPIISL351842" "EPIISL351843" "EPIISL351848" "EPIISL351849"<br>"EPIISL351874" "EPIISL353332" "EPIISL353346" "EPIISL353439" "EPIISL355491" "EPIISL355508"<br>"EPIISL355524" "EPIISL355529" "EPIISL362255" "EPIISL362257" "EPIISL362284" "EPIISL364140"<br>"EPIISL364141" "EPIISL364156" "EPIISL364554" "EPIISL377817" "EPIISL377818" "EPIISL390404"<br>"EPIISL409210" "EPIISL409510" "EPIISL409511" "EPIISL409717" "EPIISL409736" "EPIISL409764"<br>"EPIISL62231" "EPIISL62338" "EPIISL71367" "EPIISL78853" "EPIISL78854" "EPIISL78855"	"EPIISL338623" "EPIISL342243" "EPIISL346055" "EPIISL346056" "EPIISL346057" "EPIISL346059" "EPIISL346220" "EPIISL346222"<br>"EPIISL347934" "EPIISL347940" "EPIISL347941" "EPIISL347944" "EPIISL347947" "EPIISL347950" "EPIISL347951" "EPIISL347952"<br>"EPIISL348141" "EPIISL348142" "EPIISL348146" "EPIISL348160" "EPIISL350071" "EPIISL351879" "EPIISL352057" "EPIISL352058"<br>"EPIISL353449" "EPIISL353513" "EPIISL353514" "EPIISL353543" "EPIISL355535" "EPIISL355536" "EPIISL355540" "EPIISL355541"<br>"EPIISL355602" "EPIISL355604" "EPIISL355607" "EPIISL355634" "EPIISL355637" "EPIISL355762" "EPIISL356223" "EPIISL356224"<br>"EPIISL356225" "EPIISL356292" "EPIISL356293" "EPIISL356294" "EPIISL356295" "EPIISL356296" "EPIISL356297" "EPIISL356299"<br>"EPIISL356300" "EPIISL356303" "EPIISL356309" "EPIISL356311" "EPIISL356312" "EPIISL362170" "EPIISL362172" "EPIISL362173"<br>"EPIISL362175" "EPIISL362177" "EPIISL362178" "EPIISL362179" "EPIISL362180" "EPIISL362181" "EPIISL362182" "EPIISL362188"<br>"EPIISL362213" "EPIISL362214" "EPIISL362217" "EPIISL362220" "EPIISL362221" "EPIISL362222" "EPIISL362224" "EPIISL362245"<br>"EPIISL362246" "EPIISL362250" "EPIISL362252" "EPIISL362254" "EPIISL362341" "EPIISL362389" "EPIISL362390" "EPIISL362391"<br>"EPIISL362393" "EPIISL362394" "EPIISL362396" "EPIISL363855" "EPIISL363867" "EPIISL363869" "EPIISL363870" "EPIISL363871"<br>"EPIISL363873" "EPIISL363875" "EPIISL363876" "EPIISL363877" "EPIISL363878" "EPIISL363880" "EPIISL363881" "EPIISL363884"<br>"EPIISL363888" "EPIISL363892" "EPIISL363893" "EPIISL363895" "EPIISL363896" "EPIISL363897" "EPIISL363898" "EPIISL363899"<br>"EPIISL363900" "EPIISL363901" "EPIISL363902" "EPIISL363904" "EPIISL363905" "EPIISL363906" "EPIISL363907" "EPIISL363957"<br>"EPIISL363958" "EPIISL363962" "EPIISL363963" "EPIISL363968" "EPIISL363969" "EPIISL363970" "EPIISL363975" "EPIISL363976"<br>"EPIISL364000" "EPIISL364001" "EPIISL364002" "EPIISL364003" "EPIISL364005" "EPIISL364010" "EPIISL364011" "EPIISL364540"<br>"EPIISL364541" "EPIISL365767" "EPIISL365768" "EPIISL365769" "EPIISL365770" "EPIISL365771" "EPIISL365772" "EPIISL365773"<br>"EPIISL365774" "EPIISL365775" "EPIISL365776" "EPIISL365778" "EPIISL365780" "EPIISL365781" "EPIISL365782" "EPIISL365783"<br>"EPIISL365784" "EPIISL365785" "EPIISL365790" "EPIISL365834" "EPIISL365837" "EPIISL365974" "EPIISL366006" "EPIISL368194"<br>"EPIISL368204" "EPIISL368206" "EPIISL377899" "EPIISL377900" "EPIISL377901" "EPIISL377902" "EPIISL377904" "EPIISL381578"<br>"EPIISL381580" "EPIISL381582" "EPIISL381583" "EPIISL381584" "EPIISL381586" "EPIISL381588" "EPIISL381590" "EPIISL381592"<br>"EPIISL381593" "EPIISL381598" "EPIISL381599" "EPIISL390122" "EPIISL390125" "EPIISL392520" "EPIISL392521" "EPIISL392523"<br>"EPIISL392524" "EPIISL392526" "EPIISL393567" "EPIISL393571" "EPIISL393572" "EPIISL393574" "EPIISL393575" "EPIISL393576"<br>"EPIISL393579" "EPIISL393581" "EPIISL393582" "EPIISL393583" "EPIISL393584" "EPIISL393588" "EPIISL393595" "EPIISL393596"<br>"EPIISL393682" "EPIISL393684" "EPIISL393685" "EPIISL393686" "EPIISL393687" "EPIISL393688" "EPIISL393689" "EPIISL393690"<br>"EPIISL393691" "EPIISL393692" "EPIISL393693" "EPIISL393694" "EPIISL393695" "EPIISL393696" "EPIISL393697" "EPIISL393698"<br>"EPIISL393699" "EPIISL393700" "EPIISL393701" "EPIISL393702" "EPIISL393703" "EPIISL393705" "EPIISL393706" "EPIISL393707"<br>"EPIISL393708" "EPIISL393709" "EPIISL393711" "EPIISL393712" "EPIISL393713" "EPIISL393714" "EPIISL393715" "EPIISL393717"<br>"EPIISL393718" "EPIISL393719" "EPIISL393721" "EPIISL393723" "EPIISL393725" "EPIISL393726" "EPIISL393729" "EPIISL393730"<br>"EPIISL393732" "EPIISL393733" "EPIISL393734" "EPIISL393736" "EPIISL393737" "EPIISL393738" "EPIISL393740" "EPIISL393742"<br>"EPIISL393745" "EPIISL393748" "EPIISL393751" "EPIISL393757" "EPIISL393758" "EPIISL393759" "EPIISL393781" "EPIISL393782"<br>"EPIISL393937" "EPIISL393938" "EPIISL393940" "EPIISL393941" "EPIISL393946" "EPIISL393955" "EPIISL393956" "EPIISL393960"<br>"EPIISL393963" "EPIISL393967" "EPIISL394051" "EPIISL394052" "EPIISL394106" "EPIISL394107" "EPIISL394108" "EPIISL394110"<br>"EPIISL394111" "EPIISL394112" "EPIISL394923" "EPIISL394924" "EPIISL394948" "EPIISL394949" "EPIISL394950" "EPIISL397182"<br>"EPIISL397199" "EPIISL398311" "EPIISL398313" "EPIISL398314" "EPIISL398315" "EPIISL398532" "EPIISL398534" "EPIISL398535"<br>"EPIISL398555" "EPIISL398790" "EPIISL398795" "EPIISL400779" "EPIISL400781" "EPIISL400782" "EPIISL400789" "EPIISL400792"<br>"EPIISL400887" "EPIISL402407" "EPIISL402502" "EPIISL402504" "EPIISL402506" "EPIISL402508" "EPIISL402765" "EPIISL402766"<br>"EPIISL402768" "EPIISL402769" "EPIISL402771" "EPIISL402772" "EPIISL402773" "EPIISL402774" "EPIISL402776" "EPIISL402779"<br>"EPIISL402780" "EPIISL409343" "EPIISL409346" "EPIISL409350" "EPIISL409353" "EPIISL409356" "EPIISL409357" "EPIISL409358"<br>"EPIISL409359" "EPIISL409361" "EPIISL409407" "EPIISL409410" "EPIISL409411" "EPIISL409412" "EPIISL409413" "EPIISL409414"<br>"EPIISL409437" "EPIISL77944" "EPIISL84053" "EPIISL89055" "EPIISL89056" "EPIISL89058" "EPIISL89059" "EPIISL89060"<br>"EPIISL89061" "EPIISL89063" "EPIISL89064" "EPIISL89065" "EPIISL89068" "EPIISL89069" "EPIISL89074" "EPIISL89077"<br>"EPIISL89078" "EPIISL89079" "EPIISL89081" "EPIISL89082" "EPIISL89084" "EPIISL89085" "EPIISL89086" "EPIISL89090"<br>"EPIISL89091" "EPIISL89092" "EPIISL89093" "EPIISL89097" "EPIISL89098" "EPIISL89100" "EPIISL89103" "EPIISL89105"<br>"EPIISL89107" "EPIISL89111" "EPIISL89112" "EPIISL93638"

Supplementary Table 9: Accession numbers for the Africa influenza, clade references, and vaccine viral sequences per subtype downloaded from GISAID database (accessed on 27th February 2020) and analysed in this study. We gratefully acknowledge the authors from the originating laboratories responsible for obtaining the specimens and the submitting laboratories where genetic sequence data were generated and shared via the GISAID EpiFlu Initiative, on which this research is based. All submitters of data may be contacted directly via www.gisaid.org[10].

References

1. Larsson, A. AliView: a fast and lightweight alignment viewer and editor for large datasets. *Bioinformatics* **30**, 3276–3278 (2014).
2. ECDC. Influenza Virus Characterisation Reports, summary Europe. *European Centre for Disease Prevention and Control* https://www.ecdc.europa.eu/en/seasonal-influenza/surveillance-and-disease-data/influenza-virus-characterisation (2010).
3. Lutwama, J. J. *et al.* Clinic- and hospital-based sentinel influenza surveillance, Uganda 2007-2010. *J. Infect. Dis.* **206 Suppl 1**, S87-93 (2012).
4. C.D.C. *Protocol of real time RTPCR for influenza A(H1N1). The WHO Collaborating Centre for influenza at CDC*. vol. 9 (USA, 2009).
5. Zhou, B. *et al.* Multiplex Reverse Transcription-PCR for Simultaneous Surveillance of Influenza A and B Viruses. *J. Clin. Microbiol.* **55**, 3492–3501 (2017).
6. Xu, H. *et al.* FastUniq: A Fast De Novo Duplicates Removal Tool for Paired Short Reads. *PLOS ONE* **7**, e52249 (2012).
7. Bolger, A. M., Lohse, M. & Usadel, B. Trimmomatic: a flexible trimmer for Illumina sequence data. *Bioinformatics* **30**, 2114–2120 (2014).
8. Shepard, S. S. *et al.* Viral deep sequencing needs an adaptive approach: IRMA, the iterative refinement meta-assembler. *BMC Genom* **17**, (2016).
9. Edgar, R. C. MUSCLE: multiple sequence alignment with high accuracy and high through put. *Nucleic Acid Res* **32**, (2004).
10. Shu, Y. & McCauley, J. GISAID: Global initiative on sharing all influenza data – from vision to reality. *Eurosurveillance* **22**, 30494 (2017).
